# Supplementary material for: Orthogonally-tunable and ER-targeting fluorophores detect avian influenza virus early infection
Source: Nat Commun. 2022 Oct 4;13:5841. doi: 10.1038/s41467-022-33586-1 (PMC9529605; doi:10.1038/s41467-022-33586-1)
Supplement: Supplementary file 1 — Supplementary Information [file 41467_2022_33586_MOESM1_ESM.pdf]

Supplementary Information

Orthogonally-Tunable and ER-Targeting Fluorophores Detect Avian Influenza Virus Early Infection

Taewon Kang,[1,†] Md Mamunul Haque,[2,†] Boran Lee,[1,†] Kyungtae Hong,[3] Seong Cheol Hong,[3] Younghun Kim,[1] Jesang Lee,[1] Jun-Seok Lee,[2,*] and Dongwhan Lee[1,*]

[1]*Department of Chemistry, Seoul National University, 1 Gwanak-ro, Gwanak-gu, Seoul 08826, Korea*
[2]*Department of Pharmacology, Korea University College of Medicine, 73 Goryeodae-ro, Seongbuk-gu, Seoul 02841, Korea*
[3]*Bio-Med Program, KIST-School UST, Hwarang-ro 14 gil 5, Seongbuk-gu, Seoul 02792, Korea*
[†]These authors contributed eqaually to this work.

Supplementary Methods

General Considerations. All reagents were purchased from commercial suppliers and used as received unless otherwise noted. Acetonitrile and Et_2O used for spectroscopic studies were saturated with nitrogen and purified by passage through activated Al_2O_3 columns under nitrogen (Innovative Technology SPS PureSolv MD4). Spectroscopic grade cyclohexane, $CHCl_3$, *i*PrOH, HPLC-grade EtOH, and DMSO were used for solvent-dependent photophysical measurements. All air-sensitive manipulations were carried out under nitrogen atmosphere by standard Schlenk-line techniques.

4,7-Bis(5-methylpyridin-2-yl)-2,1,3-benzothiadiazole (1). Method A. A literature procedure[1] was adapted by using 2-(tributylstannyl)-5-methylpyridine[2] instead of 2-(tributylstannyl)pyridine. An oven-dried 250-mL 3-neck round-bottom flask was charged with 4,7-dibromo-2,1,3-benzothiadiazole (6.02 g, 20.5 mmol), $Pd(PPh_3)_4$ (519 mg, 0.449 mmol), and dry toluene (50 mL) under Ar, and heated at reflux. A solution of freshly prepared stannyl compound (18.35 g, 48.01 mmol) in toluene (8 mL) was added to the mixture, and the resulting brown solution was heated at reflux for 19 h. After cooling to r.t., aq KF solution (2 M) was added to remove tin byproducts from the mixture. After stirring for 30 min, the solution was filtered. The filtered solid material was washed with CH_2Cl_2 until the filtrate was no longer yellow. The combined filtrates were washed with sat'd aq NH_4Cl solution. The organic layer was dried over anhyd $MgSO_4$, filtered, and concentrated under reduced pressure. The residual material was purified by flash column chromatography on SiO_2 (hexane:CH_2Cl_2:EtOAc = 8:1:1 to 1:1:1, v/v, with a few drops of Et_3N) to furnish **1** as a yellow solid (6.39 g, 20.1 mmol, yield = 98%).

Method B. A suspension of 4,7-bis(4,4,5,5-tetramethyl-1,3,2-dioxaborolan-2-yl)benzo[*c*][1,2,5]-thiadiazole[3] (1.484 g, 3.824 mmol), 2-bromo-5-methylpyridine (1.445 g, 8.397 mmol), $Pd(PPh_3)_4$ (226 mg, 0.196 mmol), and K_2CO_3 (3.226 g, 23.34 mmol) in a mixture of toluene (28 mL) and H_2O (6.5 mL) was stirred heated at 80 °C under Ar for 18 h. After the reaction was complete, the mixture was cooled to r.t., poured into water (40 mL), and extracted into CH_2Cl_2 (20 mL × 3). The combined extracts were dried over anhyd Na_2SO_4, filtered, and concentrated under reduced pressure. The residual material was purified by flash column chromatography on SiO_2 (hexane:EtOAc = 2:1

to 1:1, v/v) to furnish **1** as a yellow solid (0.829 g, 2.61 mmol, yield = 68%). ^{1}H NMR (300 MHz, $CDCl_3$, 298 K): δ 8.62 (m, 2H), 8.56 (d, J = 8.1 Hz, 2H), 8.53 (s, 2H), 7.67 (dd, J = 8.1, 2.9 Hz, 2H), 2.42 (s, 6H). ^{13}C NMR (75 MHz, $CDCl_3$, 298 K): δ 153.9, 151.6, 150.4, 137.1, 132.9, 132.2, 129.3, 124.6, 18.5. FT-IR (ATR, cm^{-1}): 3426, 2980, 2947, 2920, 2862, 1595, 1584, 1568, 1541, 1491, 1466, 1381, 1350, 1298, 1283, 1260, 1215, 1155, 1142, 1115, 1043, 1032, 984, 947, 924, 895, 858, 843, 829, 808, 752. HRMS (ESI) calcd for $C_{18}H_{15}N_4S$ $[M + H]^+$ 319.1012, found 319.1016.

3,6-Bis(5-methylpyridin-2-yl)benzene-1,2-diamine (2). A suspension of **1** (1.47 g, 4.62 mmol), $CoCl_2{\cdot}6\,H_2O$ (183 mg, 0.769 mmol) in EtOH (10 mL) was stirred at 0 °C. To the yellow mixture was added $NaBH_4$ (1.74 g, 46.1 mmol) over a period of 20 min. The formation of a black solid material was observed, and the mixture immediately turned dark. The reaction mixture was heated at reflux for 30 min. After cooling to r.t., water was added slowly to quench unreacted $NaBH_4$. The mixture was filtered through Celite 545, and the filtrate extracted with CH_2Cl_2 three times. The combined extracts were washed with brine, dried over anhyd $MgSO_4$, filtered, and concentrated under reduced pressure. The residual material was purified by flash column chromatography on SiO_2 (hexane:EtOAc:CH_2Cl_2 = 2:1:0.5 to 1:1:0.5, v/v, with a few drops of Et_3N) to furnish **2** as a yellow solid (1.21 g, 4.18 mmol, yield = 91%). ^{1}H NMR (500 MHz, $CDCl_3$, 298 K): δ 8.49 (s, 2H), 7.62 (d, J = 8.2 Hz, 2H), 7.58 (d, J = 8.2 Hz, 2H), 7.12 (s, 2H), 5.55 (s, 4H), 2.37 (s, 6H). ^{13}C NMR (125 MHz, $CDCl_3$, 298 K): δ 156.8, 148.3, 137.6, 136.4, 130.6, 123.1, 122.2, 118.9, 18.3. FT-IR (ATR, cm^{-1}): 3391, 3364, 3302, 3264, 3098, 3042, 2995, 2918, 2860, 1634, 1601, 1564, 1537, 1499, 1470, 1435, 1375, 1317, 1298, 1287, 1244, 1229, 1152, 1142, 1065, 1030, 991, 941, 916, 883, 835, 799, 758, 737. HRMS (ESI) calcd for $C_{18}H_{19}N_4$ $[M + H]^+$ 291.1604, found 291.1607.

EliF-1a. A mixture of **2** (105.7 mg, 0.3640 mmol) and 4-ethylbenzaldehyde (0.05 mL, 0.4 mmol) in EtOH (20 mL) was stirred at reflux for 15 min. After the solution became transparent, a solution of $Na_2S_2O_5$ (57.8 mg, 0.304 mmol) in H_2O (0.34 mL) was added, and the mixture was stirred at reflux for 12 h. After the reaction was complete, the mixture was concentrated under reduced pressure and suspended in H_2O (20 mL). The solid material was isolated by filtration, washed with H_2O (20 mL × 2), and dried under an air stream to furnish **EliF-1a** as a yellow solid (128.3 mg, 0.3172 mmol, yield = 87%). ^{1}H NMR (300 MHz, $CDCl_3$, 298 K): δ 8.61 (s, 4H), 8.11 (m, 4H), 7.67 (d, J = 8.0 Hz, 2H), 7.38 (d, J = 7.3 Hz, 2H), 2.75 (q, J = 7.5 Hz, 2H), 2.42 (s, 6H), 1.31 (t, J = 7.5 Hz, 3H). ^{13}C NMR (75 MHz, CD_2Cl_2, 298 K): δ 153.3, 152.1, 149.6, 146.7, 137.5, 131.7, 128.6, 127.8, 127.0, 120.7, 29.0, 18.5, 15.6. FT-IR (ATR, cm^{-1}): 3341, 3092, 3065, 3028, 2992, 2965, 2926, 2887, 2870, 1611, 1599, 1564, 1539, 1516, 1476, 1435, 1393, 1366, 1327, 1300, 1269, 1248, 1234, 1196, 1153, 1142, 1121, 1065, 1045, 1030, 1016, 970, 949, 932, 914, 851, 839, 812, 787, 752, 739. HRMS (ESI) calcd for $C_{27}H_{25}N_4$ $[M + H]^+$ 405.2074, found 405.2076.

EliF-1b. A mixture of **2** (99.8 mg, 0.344 mmol) and 4-cyanobenzaldehyde (45.0 mg, 0.343 mmol) in EtOH (18 mL) was stirred at reflux for 15 min. After the solution became transparent, a solution of $Na_2S_2O_5$ (55.1 mg, 0.290 mmol) in H_2O (0.3 mL) was added, and the mixture was stirred at reflux for 22 h. After the reaction was complete, the mixture was cooled to r.t., concentrated under reduced pressure, and suspended in H_2O (20 mL). The solid material was isolated by filtration and washed with H_2O (20 mL × 2) to furnish **EliF-1b** as a yellow solid (104.7 mg, 0.2608 mmol, yield = 76%). ^{1}H NMR (300 MHz, $CDCl_3$, 298 K): δ 12.62 (br, 1H), 9.02 (br, 1H), 8.61 (dd, J = 1.5, 0.7 Hz, 2H), 8.29 (m, 3H), 7.99 (br, 2H), 7.81 (m, 2H), 7.68 (dd, J = 8.1, 1.5 Hz, 2H), 2.43 (s, 6H). ^{13}C NMR (75 MHz, $CDCl_3$, 298 K): δ 152.9, 149.7, 149.4, 137.5, 134.4, 132.8, 132.0, 127.3, 121.5, 118.7, 113.2, 18.5. FT-IR (ATR, cm^{-1}): 3354, 2988, 2918, 2357, 2830, 2228, 1612, 1601, 1568, 1558, 1539, 1520, 1506, 1474, 1456, 1429, 1393, 1364, 1339, 1302, 1279, 1269, 1248, 1221, 1198, 1177, 1159,

1140, 1121, 1061, 1030, 1018, 970, 951, 934, 914, 845, 816, 789, 770, 750, 741. HRMS (ESI) calcd for $C_{26}H_{20}N_5$ $[M + H]^+$ 402.1713, found 402.1715.

EliF-1c. A mixture of **2** (87.4 mg, 0.301 mmol) and 4-(dimethylamino)benzaldehyde (44.9 mg, 0.301 mmol) in EtOH (12.5 mL) was heated at reflux until the solution became transparent. A solution of $Na_2S_2O_5$ (44.5 mg, 0.234 mmol) in H_2O (0.3 mL) was added, and the mixture was stirred at reflux for 6 h. After the reaction was complete, the mixture was cooled to r.t., concentrated under reduced pressure, and suspended in H_2O (20 mL). The solid material was isolated by filtration and washed with H_2O (20 mL × 2) to furnish **EliF-1c** as an orange solid (108.2 mg, 0.2579 mmol, yield = 86%). ^1H NMR (300 MHz, CD_2Cl_2, 298 K): δ 8.61 (d, J = 2.2 Hz, 4H), 8.08 (m, 4H), 7.68 (dd, J = 8.2, 2.2 Hz, 2H), 6.86 (d, J = 9.0 Hz, 2H), 3.07 (s, 6H), 2.42 (s, 6H). ^{13}C NMR (75 MHz, CD_2Cl_2, 298 K): δ 153.7, 153.2, 152.3, 150.0, 137.7, 132.1, 128.4, 122.6, 120.4, 118.0, 112.4, 40.6, 18.6. FT-IR (ATR, cm^{-1}): 3323, 3067, 3030, 2914, 2812, 1612, 1566, 1549, 1516, 1474, 1437, 1393, 1360, 1304, 1273, 1246, 1229, 1198, 1169, 1153, 1138, 1123, 1101, 1069, 1032, 1005, 989, 972, 945, 932, 851, 818, 800, 746, 714. HRMS (ESI) calcd for $C_{27}H_{26}N_5$ $[M + H]^+$ 420.2183, found 420.2187.

EliF-1d. A mixture of **2** (145 mg, 0.499 mmol) and picolylaldehyde (55 mg, 0.50 mmol) in DMF (4.5 mL) and H_2O (0.5 mL) was stirred at 80 °C for 18 h. The mixture was concentrated under reduced pressure and the residual material was purified by flash column chromatography on SiO_2 (CH_2Cl_2:MeOH = 30:1, v/v) to furnish **EliF-1d** as a yellow solid (167 mg, 0.442 mmol, 88%). ^1H NMR (300 MHz, $CDCl_3$, 298 K): δ 12.70 (br, 1H), 9.05 (br, 1H), 8.73 (m, 1H), 8.66 (s, 2H), 8.55 (m, 1H), 8.10 (br, 3H), 7.86 (td, J = 7.7, 1.7 Hz, 1H), 7.66 (d, J = 7.4 Hz, 2H), 7.36 (ddd, J = 7.5, 4.9, 1.1 Hz, 1H), 2.42 (s, 6H). ^{13}C NMR (75 MHz, $CDCl_3$, 298 K): δ 153.2, 151.4, 149.9, 149.5, 148.9, 137.4, 136.9, 131.7, 124.4, 122.1, 121.3, 18.5. FT-IR (ATR, cm^{-1}): 3397, 3065, 2997, 2957, 2922, 2855, 1732, 1595, 1566, 1531, 1516, 1476, 1458, 1449, 1408, 1391, 1364, 1325, 1300, 1271, 1248, 1240, 1221, 1204, 1159, 1152, 1140, 1123, 1090, 1030, 999, 974, 964, 949, 930, 918, 893, 851, 812, 797, 748. HRMS (ESI) calcd for $C_{24}H_{20}N_5$ $[M + H]^+$ 378.1713, found 378.1715.

EliF-2a. Under a nitrogen atmosphere, a 25-mL Schlenk round-bottom flask was charged with a solution of **EliF-1a** (45.9 mg, 0.114 mmol) in dichloroethane (1.9 mL). A portion of $^iPr_2NEt$ (0.08 mL, 0.5 mmol) and $BF_3{\cdot}OEt_2$ (0.07 mL, 0.6 mmol) were added subsequently, and the mixture stirred at r.t. for 1 h. The reaction was quenched by adding H_2O (20 mL) and extracted into CH_2Cl_2 (20 mL × 3). The combined extracts were dried over Na_2SO_4, filtered, and concentrated under reduced pressure. Flash column chromatography on SiO_2 (hexane:EtOAc = 1:1 → CH_2Cl_2:MeOH = 20:1, v/v) furnished **EliF-2a** as a yellow solid (34.1 mg, 0.0754 mmol, yield = 66%). ^1H NMR (300 MHz, $CDCl_3$, 298 K): δ 9.26 (d, J = 8.2 Hz, 1H), 8.68 (s, 1H), 8.60 (s, 1H), 8.36 (m, 3H), 8.20 (d, J = 8.6 Hz, 1H), 7.94 (d, J = 7.3 Hz, 1H), 7.86 (d, J = 8.3 Hz, 1H), 7.71 (d, J = 6.5 Hz, 1H), 7.39 (d, J = 8.1 Hz, 2H), 2.76 (q, J = 7.6 Hz, 2H), 2.49 (s, 3H), 2.43 (s, 3H), 1.31 (t, J = 7.6 Hz, 3H). ^{13}C NMR (75 MHz, $CDCl_3$, 298 K): δ 158.3, 152.0, 150.0, 147.3, 146.4, 143.5, 143.0, 142.3, 137.4, 136.6, 133.7, 133.4, 132.6, 129.6, 129.3, 128.1, 125.8, 121.3, 120.0, 118.2, 112.5, 29.0, 18.65, 18.60, 15.6. FT-IR (ATR, cm^{-1}): 3065, 3030, 2963, 2926, 2855, 1746, 1682, 1634, 1620, 1599, 1566, 1524, 1479, 1466, 1408, 1398, 1362, 1356, 1314, 1290, 1263, 1246, 1231, 1219, 1192, 1173, 1155, 1138, 1115, 1086, 1049, 1032, 1020, 993, 978, 959, 908, 880, 847, 843, 808, 795, 781, 752, 719. HRMS (ESI) calcd for $C_{27}H_{24}BF_2N_4$ $[M + H]^+$ 453.2061, found 453.2066.

EliF-2b. Under a nitrogen atmosphere, a 25-mL Schlenk round-bottom flask was charged with a solution of **EliF-1b** (46.4 mg, 0.116 mmol) in dichloroethane (1.9 mL). A portion of $^iPr_2NEt$ (0.08 mL, 0.5 mmol) and $BF_3{\cdot}OEt_2$ (0.07 mL, 0.6 mmol) were added subsequently, and the mixture stirred

at r.t. for 75 min. The reaction was quenched by adding H_2O (20 mL) and extracted into CH_2Cl_2 (20 mL × 3). The combined extracts were dried over Na_2SO_4, filtered, and concentrated under reduced pressure. Flash column chromatography on SiO_2 (hexane:EtOAc = 1:1 → CH_2Cl_2:MeOH = 20:1, v/v) furnished **EliF-2b** as a yellow solid (46.2 mg, 0.103 mmol, yield = 89%). ^{1}H NMR (300 MHz, $CDCl_3$, 298 K): δ 9.18 (d, J = 7.9 Hz, 1H), 8.73 (s, 1H), 8.60 (m, 3H), 8.43 (d, J = 8.2 Hz, 1H), 8.31 (m, 1H), 8.08 (dd, J = 8.6, 1.7 Hz, 1H), 7.99 (d, J = 8.3 Hz, 1H), 7.83 (d, J = 8.7 Hz, 2H), 7.74 (dd, J = 8.5, 2.5 Hz, 1H), 2.58 (s, 3H), 2.45 (s, 3H). ^{13}C NMR (75 MHz, CD_2Cl_2, 298 K): δ 151.8, 150.4, 147.2, 144.6, 143.5, 142.4, 137.7, 136.9, 136.5, 135.2, 133.9, 133.6, 132.7, 130.4, 125.8, 122.1, 121.7, 120.7, 119.8, 119.3, 113.7, 113.6, 18.9, 18.7. FT-IR (ATR, cm^{-1}): 2922, 2851, 2226, 1734, 1634, 1611, 1595, 1566, 1564, 1522, 1481, 1466, 1423, 1398, 1381, 1362, 1325, 1315, 1283, 1261, 1244, 1225, 1221, 1204, 1180, 1159, 1136, 1119, 1111, 1086, 1040, 1020, 995, 988, 970, 959, 922, 887, 851, 835, 816, 795, 779, 754, 723, 712. HRMS (ESI) calcd for $C_{26}H_{19}BF_2N_5$ $[M + H]^+$ 450.1701, found 450.1704.

EliF-2c. Under a nitrogen atmosphere, a 25-mL Schlenk round-bottom flask was charged with a solution of **EliF-1c** (48.1 mg, 0.115 mmol) in dichloroethane (1.8 mL). A portion of ^{i}Pr$_2$NEt (0.08 mL, 0.5 mmol) and $BF_3{\cdot}OEt_2$ (0.07 mL, 0.6 mmol) were added subsequently, and the mixture stirred at r.t. for 1 h. The reaction was quenched by adding H_2O (20 mL) and extracted into CH_2Cl_2 (20 mL × 3). The combined extracts were dried over Na_2SO_4, filtered, and concentrated under reduced pressure. Flash column chromatography on SiO_2 (CH_2Cl_2 → CH_2Cl_2:MeOH = 20:1, v/v) furnished **EliF-2c** as a red solid (41.3 mg, 0.0884 mmol, yield = 77%). ^{1}H NMR (300 MHz, $CDCl_3$, 298 K): δ 9.29 (d, J = 8.1 Hz, 1H), 8.69 (s, 1H), 8.60 (s, 1H), 8.40 (d, J = 8.8 Hz, 2H), 8.32 (d, J = 8.2 Hz, 1H), 8.19 (d, J = 8.5 Hz, 1H), 7.94 (d, J = 7.6 Hz, 1H), 7.81 (d, J = 8.2 Hz, 1H), 7.71 (d, J = 8.4 Hz, 1H), 6.86 (d, J = 8.9 Hz, 2H). ^{13}C NMR (75 MHz, $CDCl_3$, 298 K): δ 158.9, 152.2, 151.7, 149.9, 147.5, 143.4, 143.0, 142.6, 137.4, 136.9, 133.5, 132.5, 132.4, 130.7, 125.8, 121.0, 119.9, 119.4, 117.5, 112.0, 111.9, 40.4, 18.64, 18.60. FT-IR (ATR, cm^{-1}): 2924, 2853, 1738, 1682, 1634, 1611, 1568, 1524, 1479, 1462, 1435, 1412, 1396, 1356, 1312, 1292, 1267, 1246, 1233, 1219, 1192, 1157, 1130, 1113, 1088, 1016, 976, 955, 885, 831, 814, 789, 748. HRMS (ESI) calcd for $C_{27}H_{25}BF_2N_5$ $[M + H]^+$ 468.2170, found 468.2172. Anal. Calcd for $C_{30}H_{30}BF_2N_5O$ (**EliF-2c**·CH_3COCH_3): C, 68.58; H, 5.76; N, 13.33. Found: C, 68.34; H, 5.67; N, 13.68.

EliF-2d. An oven-dried 50-mL 3-neck round-bottom flask was charged with **EliF-1d** (133 mg, 0.352 mmol) under argon. A portion of toluene (14 mL) and ^{i}Pr$_2$NEt (0.3 mL, 2 mmol) were added, and the mixture heated at reflux. To the mixture was slowly added $BF_3{\cdot}OEt_2$ (0.1 mL, 0.8 mmol) over a period of 30 min. The resulting mixture was heated at reflux for 12 h. After cooling to r.t., the mixture was concentrated under reduced pressure. Flash column chromatography on SiO_2 (hexane:acetone = 3:1 to 1:2, v/v) and recrystallization from acetone/pentane furnished **EliF-2d** (122 mg, 0.287 mmol, yield =82%). ^{1}H NMR (300 MHz, $CDCl_3$, 298 K): δ 9.21 (d, J = 8.1 Hz, 1H), 8.91 (m, 1H), 8.72 (s, 1H), 8.58 (m, 2H), 8.34 (d, J = 8.2 Hz, 1H), 8.11 (d, J = 8.5 Hz, 1H), 7.86 (m, 3H), 7.70 (dd, J = 8.1, 1.6 Hz, 1H), 7.39 (ddd, J = 7.5, 4.8, 1.1 Hz, 1H), 2.42 (s, 6H). ^{13}C NMR (75 MHz, $CDCl_3$, 298 K): δ 156.5, 151.8, 150.5, 150.1, 149.8, 146.8, 143.4, 143.2, 142.1, 137.3, 136.4, 136.3, 134.0, 133.9, 132.7, 125.8, 124.7, 124.2, 121.7, 119.8, 119.1, 113.1, 18.6. FT-IR (ATR, cm^{-1}): 2955, 2924, 2853, 1740, 1634, 1597, 1585, 1568, 1526, 1477, 1456, 1435, 1395, 1377, 1366, 1315, 1287, 1271, 1244, 1233, 1219, 1198, 1171, 1159, 1138, 1126, 1115, 1101, 1084, 1043, 1036, 997, 976, 957, 872, 851, 839, 814, 797, 791, 777, 734, 718. HRMS (ESI) calcd for $C_{24}H_{19}BF_2N_5$ $[M + H]^+$ 426.1700, found 426.1703.

EliF-3a. A 25-mL Schlenk tube was charged with **EliF-1a** (46.2 mg, 0.114 mmol) under nitrogen

atmosphere. A portion of dichloroethane (1.9 mL) and ^{i}Pr$_2$NEt (0.08 mL, 0.5 mmol) were added, and the mixture heated at reflux. To the mixture was slowly added $BF_3 \cdot OEt_2$ (0.07 mL, 0.6 mmol). The resulting mixture was stirred at reflux for 15 h. After cooling to r.t., a pale yellow solid material precipitated out, which was isolated by filtration and washed with CH_2Cl_2, MeOH, hexanes, and Et_2O to furnish **EliF-3a** (22.8 mg, 0.039 mmol, yield = 34%). ^{1}H NMR (300 MHz, DMSO, 298 K): δ 8.99 (m, 4H), 8.73 (s, 2H), 8.61 (d, J = 8.3 Hz, 2H), 8.01 (d, J = 8.1 Hz, 2H), 7.60 (d, J = 8.1 Hz, 2H), 2.81 (q, J = 7.6 Hz, 2H), 2.59 (s, 6H), 1.31 (t, J = 7.6 Hz, 3H). ^{13}C NMR (75 MHz, DMSO, 298 K): δ 156.5, 149.5, 146.4, 144.3, 143.0, 138.4, 130.3, 129.7, 128.2, 123.1, 122.8, 121.5, 118.0, 28.2, 18.0, 15.1. ^{19}F NMR (376 MHz, DMSO, 298 K): δ –129.74, –148.32. FT-IR (ATR, cm^{-1}): 3073, 2963, 1624, 1612, 1576, 1541, 1508, 1491, 1472, 1445, 1435, 1406, 1379, 1337, 1308, 1281, 1269, 1240, 1217, 1175, 1155, 1132, 1097, 1055, 1022, 997, 986, 959, 895, 854, 843, 831, 793, 773, 752, 708. HRMS (ESI) calcd for $C_{27}H_{23}B_2F_4N_4$ (cation of **EliF-3a**) 501.2049, found 501.2070. Anal. Calcd for $C_{54}H_{52}B_6F_{16}N_8O_3$ (2**EliF-3a**·3H_2O): C, 52.74; H, 4.26; N, 9.11. Found: C, 52.71; H, 4.02; N, 9.06.

EliF-3b. A 10-mL Schlenk tube was charged with **EliF-1b** (49.2 mg, 0.122 mmol) under nitrogen atmosphere. A portion of dichloroethane (2 mL) and ^{i}Pr$_2$NEt (0.1 mL, 0.5 mmol) were added, and the mixture heated at reflux. To the mixture was slowly added $BF_3 \cdot OEt_2$ (0.1 mL, 0.6 mmol). The resulting mixture was stirred at reflux for 17 h. The mixture was cooled to r.t. and a pale yellow solid material precipitated out, which was isolated by filtration and washed with CH_2Cl_2, $CHCl_3$, and THF to furnish **EliF-3b** (43.5 mg, 0.0744 mmol, yield = 61%). ^{1}H NMR (400 MHz, DMSO, 298 K): δ 9.00 (s, 4H), 8.77 (s, 2H), 8.61 (d, J = 8.3 Hz, 2H), 8.27 (dd, J = 19.6, 8.3 Hz, 4H), 2.59 (s, 6H). ^{13}C NMR (75 MHz, DMSO, 298 K): δ 153.9, 146.5, 144.5, 142.8, 138.6, 132.7, 131.2, 129.7, 128.4, 123.6, 122.9, 118.2, 117.9, 115.5, 18.0. FT-IR (ATR, cm^{-1}): 3073, 2224, 1624, 1568, 1539, 1491, 1474, 1437, 1398, 1379, 1339, 1310, 1288, 1269, 1242, 1217, 1173, 1155, 1132, 1096, 1053, 1043, 1018, 999, 988, 959, 905, 899, 864, 854, 835, 791, 760, 727. HRMS (ESI) calcd for $C_{26}H_{18}B_2F_4N_5$ (cation of **EliF-3b**) 498.1688, found 498.1676. Anal. Calcd for $C_{26}H_{22}B_3F_8N_5O_2$ (**EliF-3b**·2H_2O): C, 50.29; H, 3.57; N, 11.28. Found: C, 49.98; H, 3.45; N, 10.95.

EliF-3c. A 10-mL Schlenk tube was charged with **EliF-1c** (54.1 mg, 0.122 mmol) under nitrogen atmosphere. A portion of dichloroethane (2.2 mL) and ^{i}Pr$_2$NEt (0.09 mL, 0.5 mmol) were added, and the mixture heated at reflux for 10 min. To the mixture was slowly added $BF_3 \cdot OEt_2$ (0.08 mL, 0.6 mmol). The resulting mixture was stirred at reflux for 24 h. The mixture was cooled to r.t., diluted with CH_2Cl_2 (10 mL) and precipitates were isolated by filtration and washed with CH_2Cl_2 (10 mL × 3) to furnish **EliF-3c** as an orange solid (33.4 mg, 0.0554 mmol, yield = 45%). ^{1}H NMR (400 MHz, DMSO, 298 K): δ 9.01 (s, 2H), 8.93 (d, J = 8.4 Hz, 2H), 8.58 (m, 4H), 8.07 (d, J = 9.0 Hz, 2H), 6.95 (d, J = 9.0 Hz, 2H), 3.12 (s, 6H), 2.59 (s, 6H). ^{13}C NMR (75 MHz, DMSO, 298 K): δ 153.0, 146.2, 144.1, 143.2, 138.1, 131.9, 130.1, 122.5, 122.2, 117.3, 111.3, 109.5, 18.0. FT-IR (ATR, cm^{-1}): 3100, 3075, 1624, 1607, 1580, 1541, 1491, 1472, 1435, 1406, 1375, 1339, 1269, 1242, 1229, 1207, 1175, 1159, 1132, 1092, 1049, 1009, 993, 957, 891, 860, 837, 829, 797, 785, 781, 760, 746, 723. HRMS (ESI) calcd for $C_{27}H_{24}B_2F_4N_5$ (cation **EliF-3c**) 516.2158, found 516.2143. Anal. Calcd for $C_{27}H_{26}B_3F_8N_5O$ (**EliF-3c**·H_2O): C, 52.23; H, 4.22; N, 11.28. Found: C, 52.59; H, 4.21; N, 10.98.

EliF-3d. A 50-mL 3-neck round-bottom flask was charged with **EliF-1d** (129.8 mg, 0.3439 mmol) under nitrogen atmosphere. A portion of dichloroethane (6 mL) and ^{i}Pr$_2$NEt (0.25 mL, 1.4 mmol) were added, and the mixture heated at reflux for 10 min. To the mixture was slowly added $BF_3 \cdot OEt_2$ (0.24 mL, 1.9 mmol). The resulting mixture was stirred at reflux for 12 h. The mixture was cooled to r.t. and a brown solid material precipitated out, which was isolated by filtration and washed

with CH_2Cl_2, $CHCl_3$, and THF to furnish **EliF-3b** as a yellow solid (108.6 mg, 0.1936 mmol, yield = 57%). ^{1}H NMR (400 MHz, DMSO, 298 K): δ 9.00 (m, 5H), 8.78 (s, 2H), 8.61 (d, J = 8.2 Hz, 2H), 8.29 (m, 2H), 7.85 (m, 1H), 2.60 (s, 6H). ^{13}C NMR (100 MHz, DMSO, 298 K): δ 153.7, 150.32, 146.31, 144.43, 143.19, 142.92, 138.46, 137.61, 129.59, 127.36, 126.34, 123.62, 122.82, 118.52, 18.03. ^{19}F NMR (376 MHz, DMSO, 298 K): δ –130.79, –148.25, –148.31. FT-IR (ATR, cm^{-1}): 3094, 3069, 1623, 1591, 1572, 1543, 1514, 1493, 1449, 1431, 1342, 1333, 1317, 1290, 1265, 1240, 1205, 1161, 1111, 1092, 1047, 1007, 993, 964, 897, 854, 835, 775, 743. HRMS (ESI) calcd for $C_{24}H_{18}B_2F_4N_5$ (M^+ of **EliF-3d** = $M^+BF_4^-$) 474.1687, found 474.1663. Anal. Calcd for $C_{24}H_{20}B_3F_8N_5O$ (**EliF-3d**·H_2O): C, 49.80; H, 3.48; N, 12.10. Found: C, 49.88; H, 3.53; N, 11.91.

X-ray Crystallographic Studies on EliF-1a. Single crystals of **EliF-1a** were prepared by slow diffusion of pentane into a DCE solution of this material. A yellow crystal (approximate dimensions 0.86 × 0.097 × 0.063 mm^3) was placed onto a nylon loop with Paratone-N oil, and mounted on an XtaLAB AFC12 (RINC): Kappa dual home/near diffractometer. The data collection was carried out using Cu Kα radiation and the crystal was kept at 93 K. A total of 13138 reflections were measured (6.326° $\leq 2\theta \leq$ 158.382°). The structure was solved with SHELXT[4] using direct methods, and refined with SHELXL[5] refinement package of OLEX2.[6] A total of 4320 unique reflections were used in all calculations. The final $R1$ was 0.0721 ($I \geq 2\sigma(I)$) and $wR2$ was 0.2157 (all data).

X-ray Crystallographic Studies on EliF-1c. Single crystals of **EliF-1c** were prepared by slow diffusion of pentane into a DCE solution of this material. A dark orange crystal (approximate dimensions 0.506 × 0.361 × 0.22 mm^3) was placed onto a nylon loop with Paratone-N oil, and mounted on an XtaLAB AFC12 (RINC): Kappa dual home/near diffractometer. The data collection was carried out using Cu Kα radiation and the crystal was kept at 93 K. A total of 25286 reflections were measured (8.51° $\leq 2\theta \leq$ 158.032°). The structure was solved with SHELXT[4] using direct methods, and refined with SHELXL[5] refinement package of OLEX2.[6] A total of 4941 unique reflections were used in all calculations. The final $R1$ was 0.0594 ($I \geq 2\sigma(I)$) and $wR2$ was 0.1652 (all data).

X-ray Crystallographic Studies on EliF-1d. Single crystals of **EliF-1d** were prepared by slow diffusion of pentane into a MeCN solution of this material. A yellow crystal (approximate dimensions 0.203 × 0.073 × 0.055 mm^3) was placed onto a nylon loop with Paratone-N oil, and mounted on a SuperNova, Dual, Cu at zero, AtlasS2 diffractometer. The data collection was carried out using Cu Kα radiation and the crystal was kept at 99.9 K. A total of 10241 reflections were measured (8.256° $\leq 2\theta \leq$ 153.136°). The structure was solved with SHELXT[4] using direct methods, and refined with SHELXL[5] refinement package of OLEX2.[6] A total of 3771 unique reflections were used in all calculations. The final $R1$ was 0.0418 ($I \geq 2\sigma(I)$) and $wR2$ was 0.1161 (all data).

X-ray Crystallographic Studies on EliF-2a. Single crystals of **EliF-2a** were prepared by slow diffusion of pentane into a DCE solution of this material. A yellow crystal (approximate dimensions 0.394 × 0.223 × 0.2 mm^3) was placed onto a nylon loop with Paratone-N oil, and mounted on an XtaLAB AFC12 (RINC): Kappa dual home/near diffractometer. The data collection was carried out using Cu Kα radiation and the crystal was kept at 93 K. A total of 12150 reflections were measured (9.152° $\leq 2\theta \leq$ 158.692°). The structure was solved with SHELXT[4] using direct methods, and refined with SHELXL[5] refinement package of OLEX2.[6] A total of 2955 unique reflections were used in all calculations. The final $R1$ was 0.0978 ($I \geq 2\sigma(I)$) and $wR2$ was 0.2671 (all data).

X-ray Crystallographic Studies on EliF-2b. Single crystals of **EliF-2b** were prepared by slow diffusion of pentane into a DCE solution of this material. A yellow crystal (approximate dimensions

$0.23 \times 0.22 \times 0.108$ mm^3) was placed onto a nylon loop with Paratone-N oil, and mounted on an XtaLAB AFC12 (RINC): Kappa dual home/near diffractometer. The data collection was carried out using Cu Kα radiation and the crystal was kept at 93 K. A total of 17289 reflections were measured ($7.88° \leq 2\theta \leq 157.978°$). The structure was solved with SHELXT[4] using direct methods, and refined with SHELXL[5] refinement package of OLEX2.[6] A total of 4356 unique reflections were used in all calculations. The final $R1$ was 0.0474 ($I \geq 2\sigma(I)$) and $wR2$ was 0.1418 (all data).

X-ray Crystallographic Studies on EliF-2d. Single crystals of **EliF-2d** were prepared by slow diffusion of pentane into a DCE solution of this material. A yellow crystal (approximate dimensions $0.236 \times 0.132 \times 0.127$ mm^3) was placed onto a nylon loop with Paratone-N oil, and mounted on an XtaLAB AFC12 (RINC): Kappa dual home/near diffractometer. The data collection was carried out using Cu Kα radiation and the crystal was kept at 93 K. A total of 25000 reflections were measured ($6.448° \leq 2\theta \leq 158.512°$). The structure was solved with SHELXT[4] using direct methods, and refined with SHELXL[5] refinement package of OLEX2.[6] A total of 8150 unique reflections were used in all calculations. The final $R1$ was 0.0631 ($I \geq 2\sigma(I)$) and $wR2$ was 0.1857 (all data).

X-ray Crystallographic Studies on EliF-3d. Single crystals of **EliF-3d** were precipitated out from the boiling reaction mixture. A yellow crystal (approximate dimensions $0.309 \times 0.074 \times 0.055$ mm^3) was placed onto a nylon loop with Paratone-N oil, and mounted on a SuperNova, Dual, Cu at zero, AtlasS2 diffractometer. The data collection was carried out using Cu Kα radiation and the crystal was kept at 100 K. A total of 20182 reflections were measured ($7.868° \leq 2\theta \leq 152.988°$). The structure was solved with SHELXT[4] using direct methods, and refined with SHELXL[5] refinement package of OLEX2.[6] A total of 4991 unique reflections were used in all calculations. The final $R1$ was 0.0482 ($I \geq 2\sigma(I)$) and $wR2$ was 0.1388 (all data).

Cytotoxicity Analysis of the EliF Probes. The cytotoxicity was evaluated by chemiluminescence assay on HeLa cells. The cells were seeded in a 96-well opaque-walled clear bottom plate at a density of 1×10^4 cells/well and incubated for 24 h. Various concentrations of the EliF compounds were dissolved in DMSO (final concentration = 0.3%); cells were treated with these solutions for 2 h. In another set, cells were treated with serially diluted ER stressors (thapsigargin (TG; Sigma Aldrich-T9033) and tunicamycin (TN; Sigma Aldrich-T7765)) for 12 h.[7–10] After incubation, the bright field images were taken in Leica microscope, and the cytotoxicity assay was carried out using CellTiter-Glo 2.0 assay (Promega, G9241) according to the manufacturer's protocol. The plate was incubated at r.t. for 30 min to ensure cell lysis. The luminescence of each well was recorded subsequently by using SpectraMax microplate reader (Molecular Devices, Sunnyvale, CA, USA) and SoftMax Pro software (version 5.4). The mean luminescence values of three wells were analyzed with the non-treated cells as control. To analyze the cytotoxicity of the EliF compounds and the ER stressors, a two-tailed paired t-test was used; p-value < 0.01 was considered as statistically significant.

Photo-cytotoxicity Analysis of the EliF Probes. The photo-cytotoxicity was evaluated by chemiluminescence assay on HeLa cells. The cells were seeded in a 96-well opaque-walled clear bottom plate at a density of 1×10^4 cells/well and incubated for 24 h. Various concentrations of the EliF compounds were dissolved in DMSO (final concentration = 0.3%); cells were treated with these solutions for 30 min. Subsequently, the cells were irradiated with 365 nm UV light (6 W) for 10 sec. After irradiation, the bright field images were taken in Nikon Eclipse Ti microscope (Nikon, Japan) with NIS-Elements BR software (version 4.50) and the photo-cytotoxicity assay was carried out using CellTiter-Glo 2.0 assay (Promega, G9241) according to the manufacturer's protocol. The plate was incubated at r.t. for 30 min to ensure cell lysis. The luminescence of

each well was recorded subsequently by using SpectraMax microplate reader (Molecular Devices, Sunnyvale, CA, USA) and SoftMax Pro software (version 5.4). The mean luminescence values of three wells were analyzed with the non-treated cells as control.

Photostability Evaluation. HeLa cells were seeded in a 96-well clear bottom plate at a density of 1 × 10^4 cells/well, and cultured for 24 h. The cells were treated separately with identical concentrations (10 μM) of the EliF probes (**EliF-1b** and **EliF-2c**), and commercial ER-Green (ER-Tracker^{TM} Green, BODIPY^{TM} FL Glibenclamide; Thermo Fisher, E34251). After 30 min of incubation, the cells were washed twice with fresh DMEM media, and the fluorescence images of the probes and ER-tracker were captured by using Leica DMi8 microscope with ×20 dry lens. Photostability was studied by capturing the fluorescence images with varying exposure time (0–5 min) to laser irradiation of fixed light intensity (25%).

Fluorescence Responses of EliF Probes under ER-Stress Conditions. To investigate the fluorescence changes of EliF compounds under ER-stress conditions, TG and TN were used. HeLa cells were treated with various concentrations (0–30 μM) of TG and TN for 12 h to induce ER stress.[7–10] The cells were subsequently treated with the probes (10 μM) for 30 min. As controls, untreated cells (autofluorescence group), commercial ER-trackers (ER-Tracker^{TM} Blue-white DPX; Thermo Fisher, E12353 and ER-Tracker^{TM} Green BODIPY^{TM} FL Glibenclamide; Thermo Fisher, E34251), and mitochondria-specific ionophore (carbonyl cyanide *m*-chlorophenylhydrazone, CCCP; Sigma Aldrich-857815) to induce mitochondrial stress were used. The fluorescence responses of the EliF probes were analyzed by flow cytometry assay. For this purpose, the cells were trypsinized and centrifuged for 5 min at 136 g. The supernatant was discarded, and the remaining cell pellet was resuspended in PBS for analysis. Flow cytometry was performed on a Gallios Flow Cytometer (Beckman Coulter, USA) by counting 10,000 events for each sample with Kaluza software (version 2.1). The fluorescence signals were recorded by three different settings: FL1 (λ_{ex} = 488 nm; λ_{em} = 505–545 nm); FL9 (λ_{ex} = 405 nm; λ_{em} = 430–470 nm); FL10 (λ_{ex} = 405 nm; λ_{em} = 530–570 nm). The data was processed with FlowJo Single Cell Analysis Software v10.7.2 (FlowJo, LLC, Ashland, OR, USA). Flow cytometry data was analyzed in histograms offset plots; geometric means of the fluorescence intensity of the samples were obtained from FlowJo. The fold changes were calculated using the mean values between the highest and lowest dose treatments.

Fluorescence Responses of EliF-2c in the Monitoring of Cells Infected with Avian Influenza. To monitor AI infection at the early stage, Human Tracheal Epithelial Cells (HTEpC) (PromoCell, cat. no. C-12644), Human Umbilical Vein Endothelial Cells (HUVEC) (Cell Engineering For Origin, cat. no. CEFOgro-HUVEC), and RPMI 2650 (Korean Cell Line Bank, KCLB No. 10030) were used. Different cells (HTEpC, HUVEC, and RPMI 2650) were infected with the H1N1 virus (EID_{50}/mL; $10^{6.3}$) for 12 and 24 h, and treated subsequently with **EliF-2c** (10 μM) for 30 min. The cells were trypsinized, harvested, and centrifuged for 5 min at 136 g. The supernatants were discarded, and the remaining cell pellets were resuspended in PBS. The resulting samples were subjected to flow cytometry analysis on a Gallios Flow Cytometer (Beckman Coulter, USA) by counting 10,000 events for each sample with Kaluza software (version 2.1). The fluorescence signals were recorded by FL10 (λ_{ex} = 405 nm; λ_{em} = 530–570 nm). The data was processed with FlowJo Single Cell Analysis Software v10.7.2 (FlowJo, LLC, Ashland, OR, USA) by using histogram offset plots.

RNA Extraction and Reverse Transcription Polymerase Chain Reaction. HeLa cells were either treated with ER stressors (TG or TN; 10 μM) for 12 hr or infected with H1N1 (EID_{50}: $10^{4.567}$) for 24 h. After harvesting the cells, RNA was isolated with the

RNeasy® mini kit (Qiagen#74104) and reverse transcribed using oligo (dT) primers to synthesize the cDNA with QuantiTect® Reverse Transcription Kit (Qiagen#205311). PCR reactions were carried out at 94 °C denaturation and 60 °C annealing temperatures for 25 cycles using Phusion Hot Start II DNA polymerase (Invitrogen#F549). Primers used to amplify the cDNA were, NCAPG (F-TTCAAGGCTGGTTACGGTTC, R-AGGGCACACCAATACAAAGC), PDXDC1 (F-TAGGAACTCGGGGAGAGGAT, R-CCTTCGGGATCTGATTTCAA), PDXDC2P (F-AGATGGATGTTGCCTTCCTG, R-CCAGCACTGATGAGGAGACA), PSPH (F-GACAGCACGGTCATCAGAGA, R-CCTGAACATTTCGCTCCTGT) and ACTB (F-TGACGGGGTCACCCACACTG, R-CTAGAAGCATTTGCGGTGGA); here, ACTB was used as a control. The RT-PCR amplicons were visualized by running on a 2% agarose gel.

Supplementary Table 1. Photophysical properties of EliF molecules in $CHCl_3$ solution (T = 293 K).

	$\lambda_{abs,max}$ (nm)	$\lambda_{em,max}$ (nm)	ε ($cm^{-1}\cdot M^{-1}$)^a	Φ_F(%)	τ (ns)^b	k_r (10^8 s^{-1})	k_{nr} (10^8 s^{-1})
**EliF-1a**	345	–	3.9×10^4	< 1	–	–	–
**EliF-1b**	350	440<br>530	4.3×10^4	13	0.50<br>0.22 (-0.110), 1.47 (0.080)	–	–
**EliF-1c**	350	–	3.9×10^4	< 1	–	–	–
**EliF-1d**	345	410<br>520	3.7×10^4	18	0.34<br>0.27 (-0.097), 1.83 (0.074)	–	–
**EliF-2a**	385	480	1.6×10^4	35	2.11 (0.012), 4.80 (0.037)	0.73^c	1.4^c
**EliF-2b**	385	450	1.6×10^4	69	2.41	2.9	1.3
**EliF-2c**	385	480	1.5×10^4	34	0.61 (0.010), 5.29 (0.042)	0.64^c	1.2^c
**EliF-2d**	385	445	1.6×10^4	68	2.21	3.1	1.4
**EliF-3a**	390	445	3.5×10^4	17	1.03 (0.060), 3.14 (0.004)	1.7^c	8.1^c
**EliF-3b**	390	400<br>425	3.2×10^4	20	0.46<br>0.43 (0.086), 2.47 (0.004)	4.7^c	19^c
**EliF-3c**	390, 470 (broad)	–	3.1×10^4	< 1	–	–	
**EliF-3d**	370	400<br>425	2.8×10^4	22	0.51<br>0.49 (0.086), 2.12 (0.002)	4.5^c	16^c

^{a}Measured at the excitation wavelength of 350 nm.
^{b}Numbers in the parenthesis are pre-exponential factors of the double exponential fitting.
^{c}Determined by treating the fluorescence decay as a single-exponential of the major lifetime component.

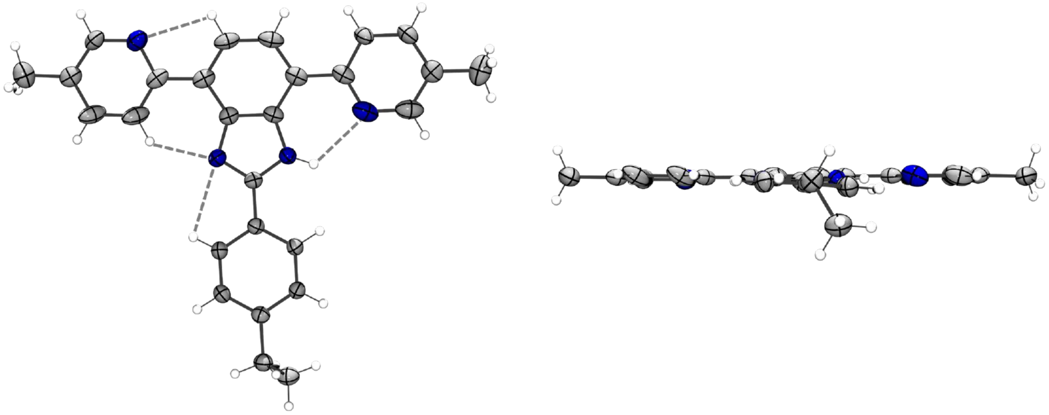

Supplementary Fig. 1. ORTEP diagrams of **EliF-1a** (left, face-on view; right, edge-on view) with thermal ellipsoids at the 50% probability level, and hydrogen bonding ($d_{\text{N–H}\cdots\text{N}}$ = 2.816 Å; $d_{\text{C–H}\cdots\text{N}}$ = 2.863–2.940 Å) indicated by dotted lines. N is blue.

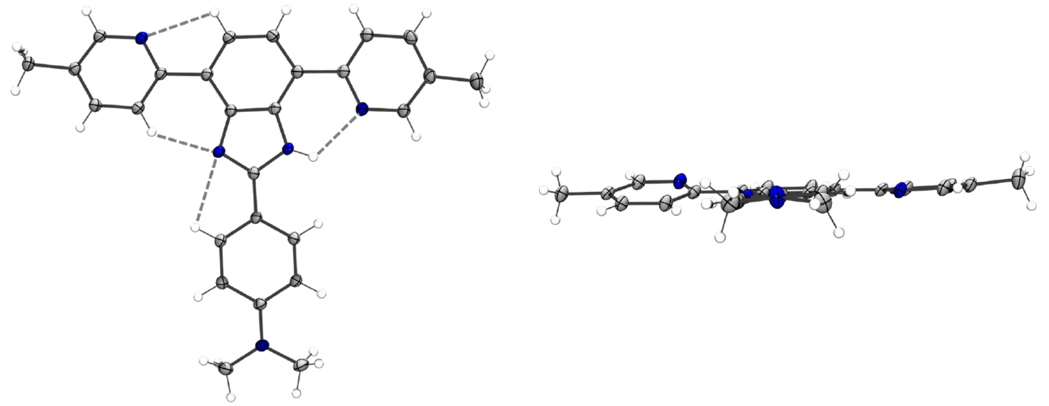

Supplementary Fig. 2. ORTEP diagrams of **EliF-1c** (left, face-on view; right, edge-on view) with thermal ellipsoids at the 50% probability level, and hydrogen bonding ($d_{\text{N–H}\cdots\text{N}}$ = 2.714 Å; $d_{\text{C–H}\cdots\text{N}}$ = 2.810–2.978 Å) indicated by dotted lines. N is blue.

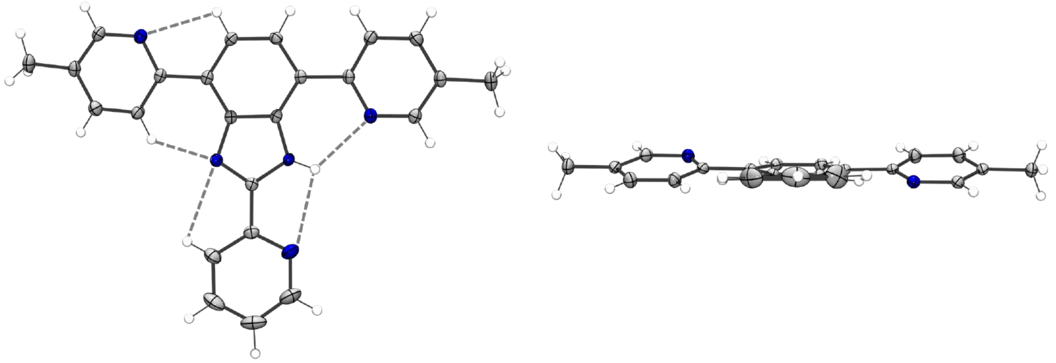

Supplementary Fig. 3. ORTEP diagrams of **EliF-1d** (left, face-on view; right, edge-on view) with thermal ellipsoids at the 50% probability level, and hydrogen bonding ($d_{\mathrm{N–H}\cdots\mathrm{N}}$ = 2.785–2.804 Å; $d_{\mathrm{C–H}\cdots\mathrm{N}}$ = 2.807–2.975 Å) indicated by dotted lines. N is blue.

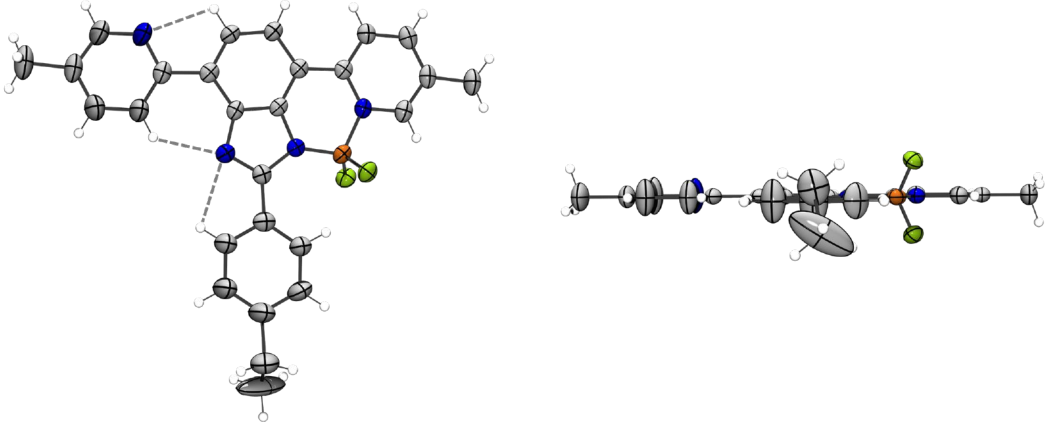

Supplementary Fig. 4. ORTEP diagrams of **EliF-2a** (left, face-on view; right, edge-on view) with thermal ellipsoids at the 50% probability level, and hydrogen bonding ($d_{\mathrm{C–H}\cdots\mathrm{N}}$ = 2.784–3.003 Å) indicated by dotted lines. N is blue, B is orange, and F is green.

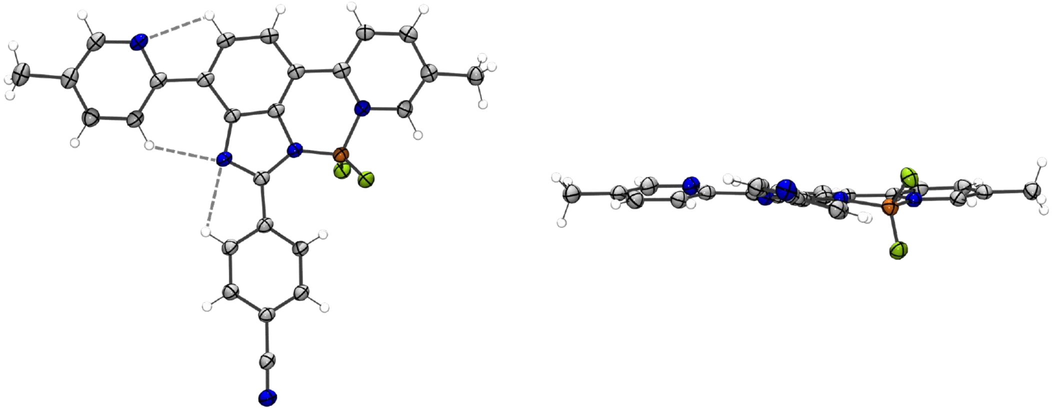

Supplementary Fig. 5. ORTEP diagrams of **EliF-2b** (left, face-on view; right, edge-on view) with thermal ellipsoids at the 50% probability level, and hydrogen bonding ($d_{\mathrm{C-H}\cdots\mathrm{N}}$ = 2.771–3.042 Å) indicated by dotted lines. N is blue, B is orange, and F is green.

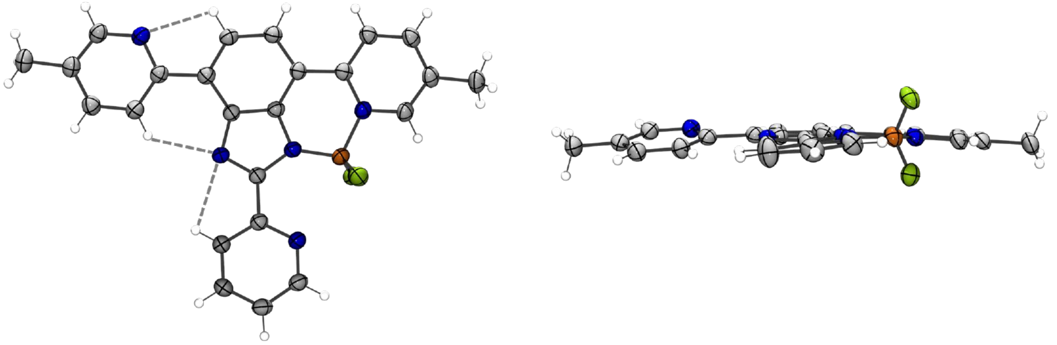

Supplementary Fig. 6. ORTEP diagrams of **EliF-2d** (left, face-on view; right, edge-on view) with thermal ellipsoids at the 50% probability level, and hydrogen bonding ($d_{\mathrm{C-H}\cdots\mathrm{N}}$ = 2.764–3.071 Å) indicated by dotted lines. N is blue, B is orange, and F is green. The asymmetric unit is comprised of two chemically equivalent yet crystallographically independent molecules, for which only one is shown here.

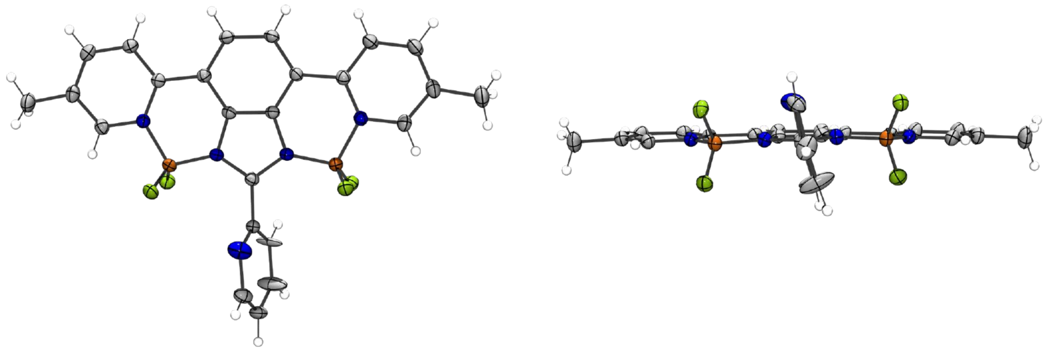

Supplementary Fig. 7. ORTEP diagrams of **EliF-3d** with thermal ellipsoids at the 50% probability level. N is blue, B is orange, and F is green. The axial pyridyl ring is disordered over two positions, for which only one conformer is shown here; the BF_4^- counteranions was omitted for clarity.

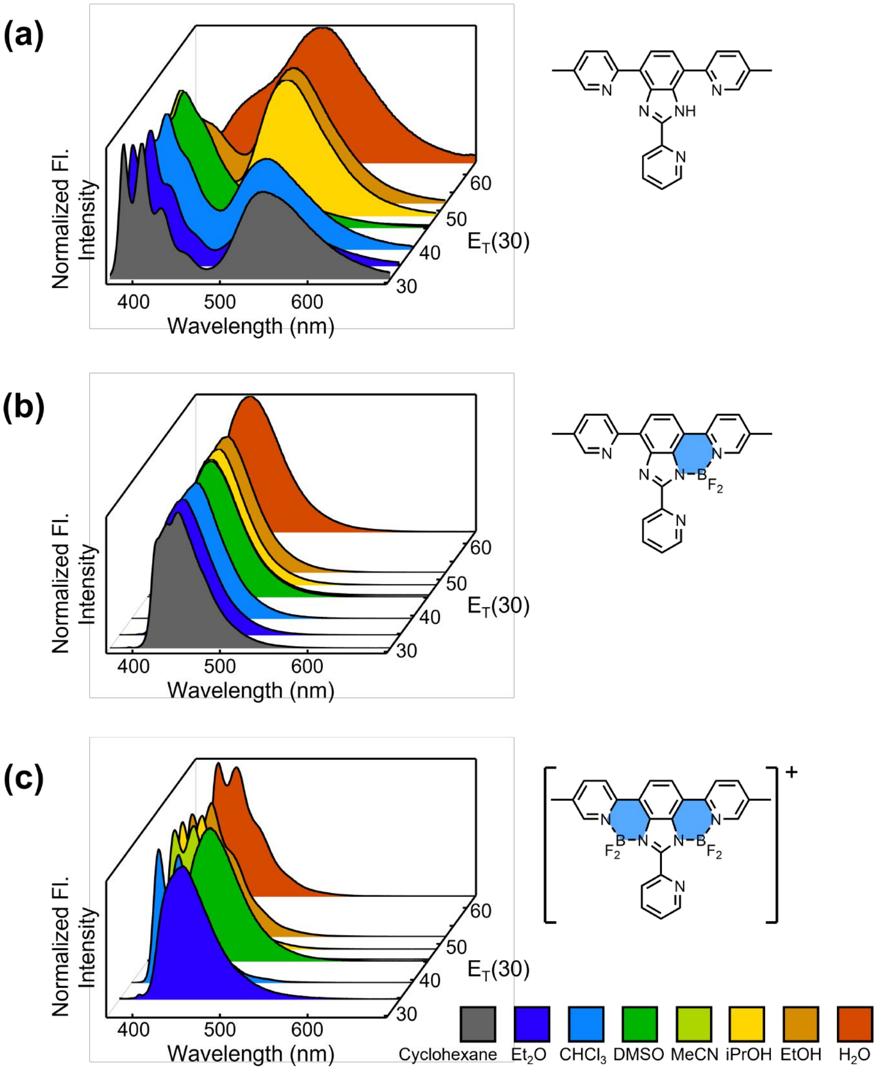

Supplementary Fig. 8. Solvent polarity-dependent changes in the normalized fluorescence spectra of (a) **EliF-1d**, (b) **EliF-2d**, and (c) **EliF-3d** in cyclohexane, Et_2O, $CHCl_3$, DMSO, MeCN, *i*PrOH, EtOH, and water (from front to back; corresponding to increasing solvent polarity as denoted by the $E_T(30)$ values in the y-axis; T = 293 K; λ_{exc} = 350 nm).

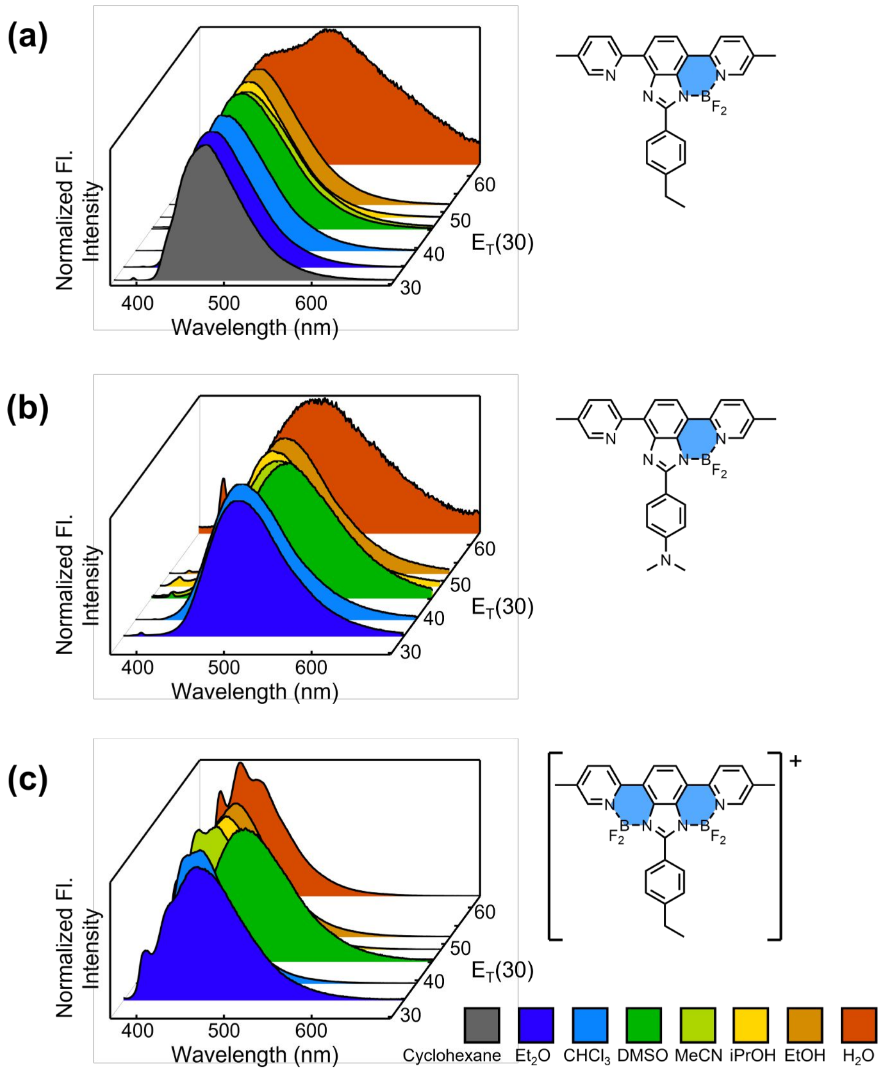

Supplementary Fig. 9. Solvent polarity-dependent changes in the normalized fluorescence spectra of (a) **EliF-2a**, (b) **EliF-2c**, and (c) **EliF-3a** in cyclohexane, Et_2O, $CHCl_3$, DMSO, MeCN, *i*PrOH, EtOH, and water (from front to back; corresponding to increasing solvent polarity as denoted by the $E_T(30)$ values in the y-axis; $T = 293$ K; $\lambda_{exc} = 350$ nm).

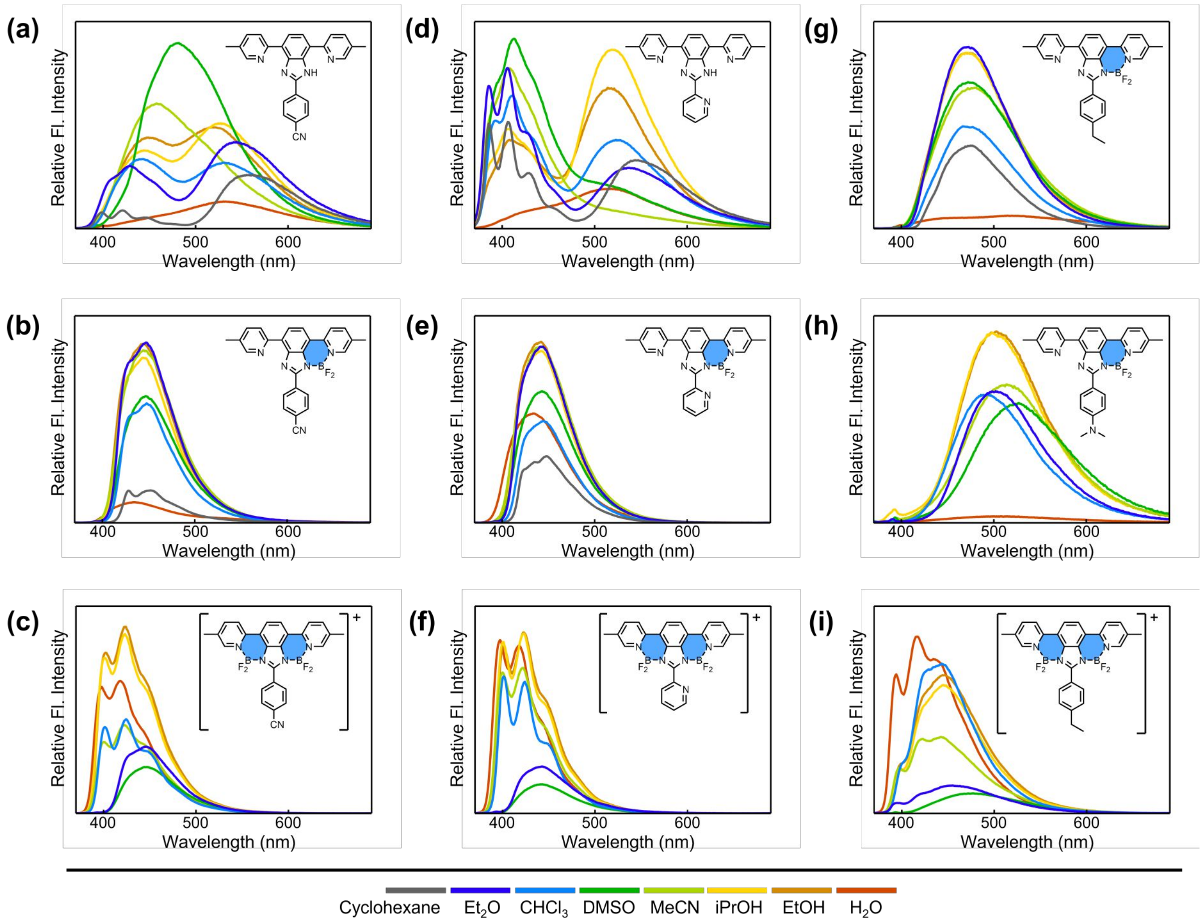

Supplementary Fig. 10. Solvent polarity-dependent changes in the relative fluorescence intensities of (a) **EliF-1b**, (b) **EliF-2b**, (c) **EliF-3b**, (d) **EliF-1d**, (e) **EliF-2d**, (f) **EliF-3d**, (g) **EliF-2a**, (h) **EliF-2c**, and (i) **EliF-3a** in cyclohexane, Et_2O, $CHCl_3$, DMSO, MeCN, *i*PrOH, EtOH, and H_2O (T = 293 K; λ_{exc} = 350 nm).

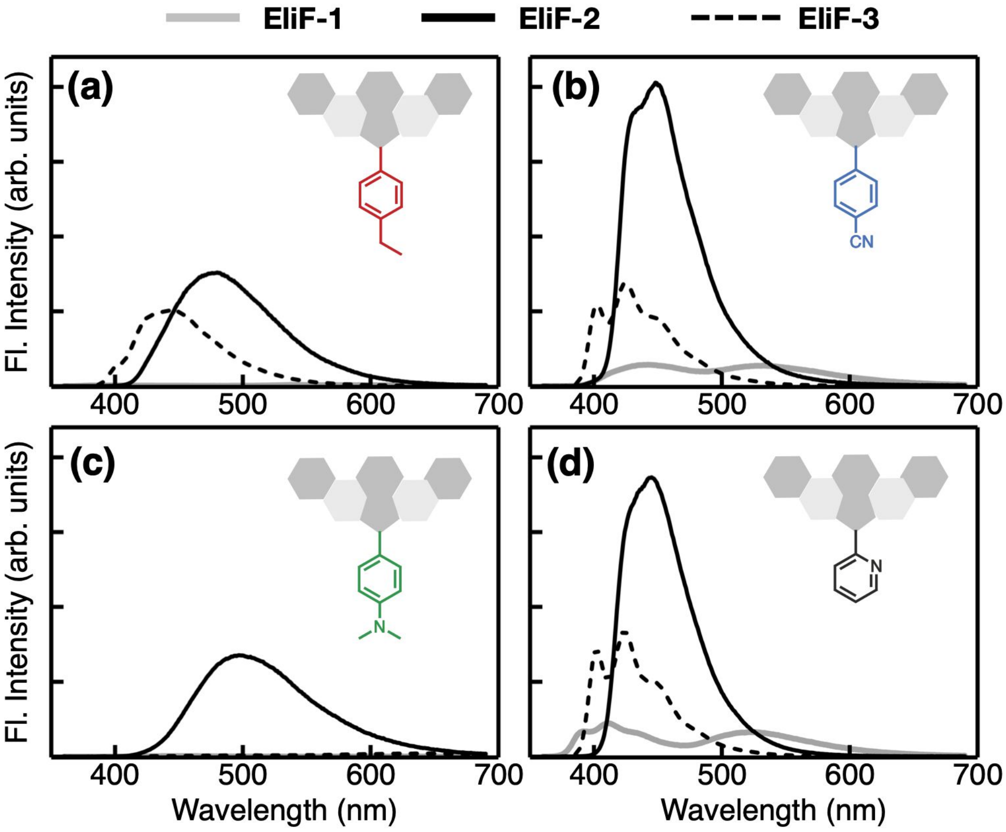

Supplementary Fig. 11. Fluorescence spectra of (a) **EliF-a**, (b) **EliF-b**, (c) **EliF-c**, and (d) **EliF-d** series (**EliF-1**, gray solid lines; **EliF-2**, black solid lines; **EliF-3**, black dashed lines) measured in $CHCl_3$ (T = 293 K; λ_{exc} = 350 nm). The emission intensities are scaled by dividing with the absorbance at λ = 350 nm, so that they are proportional to quantum yield.

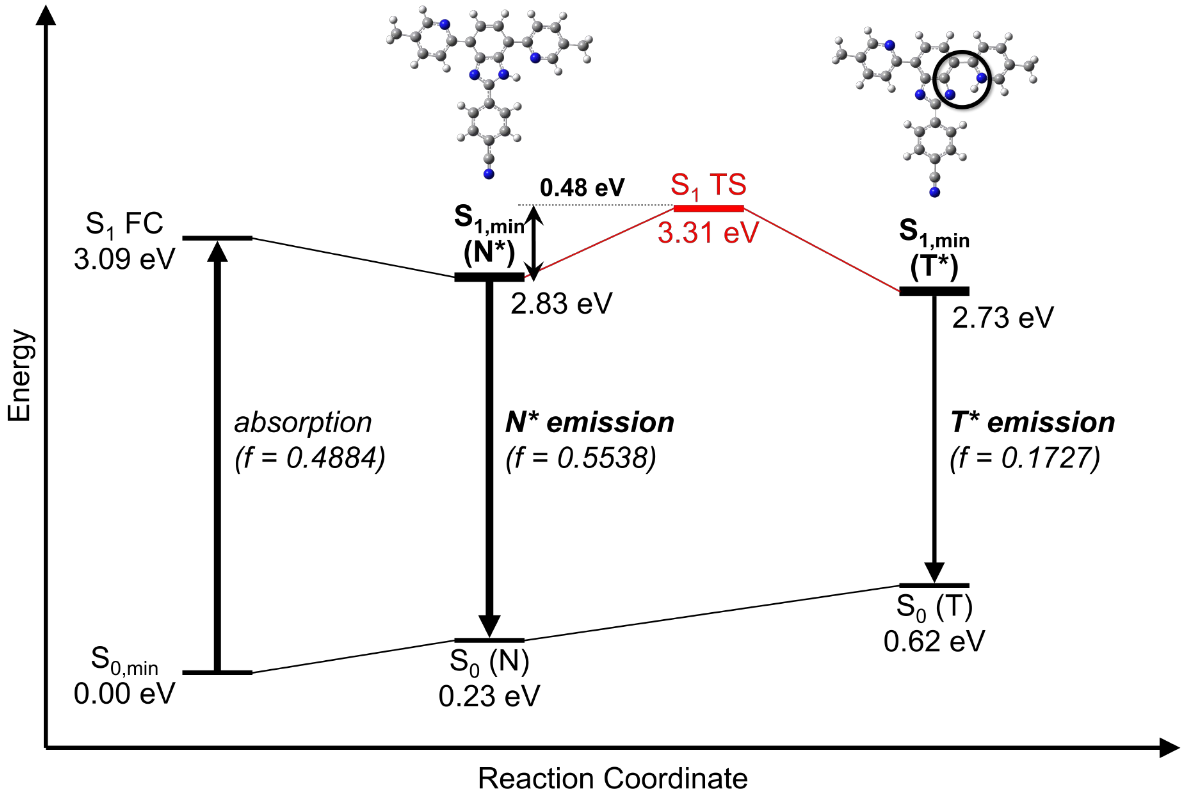

Supplementary Fig. 12. Energy diagram of **EliF-1b** in the S_1 excited-state and S_0 ground state calculated by TD-DFT (CPCM/B3LYP/6-31G(d) level of theory). Cartesian coordinates of geometry optimized structures are provided as Source Data file.

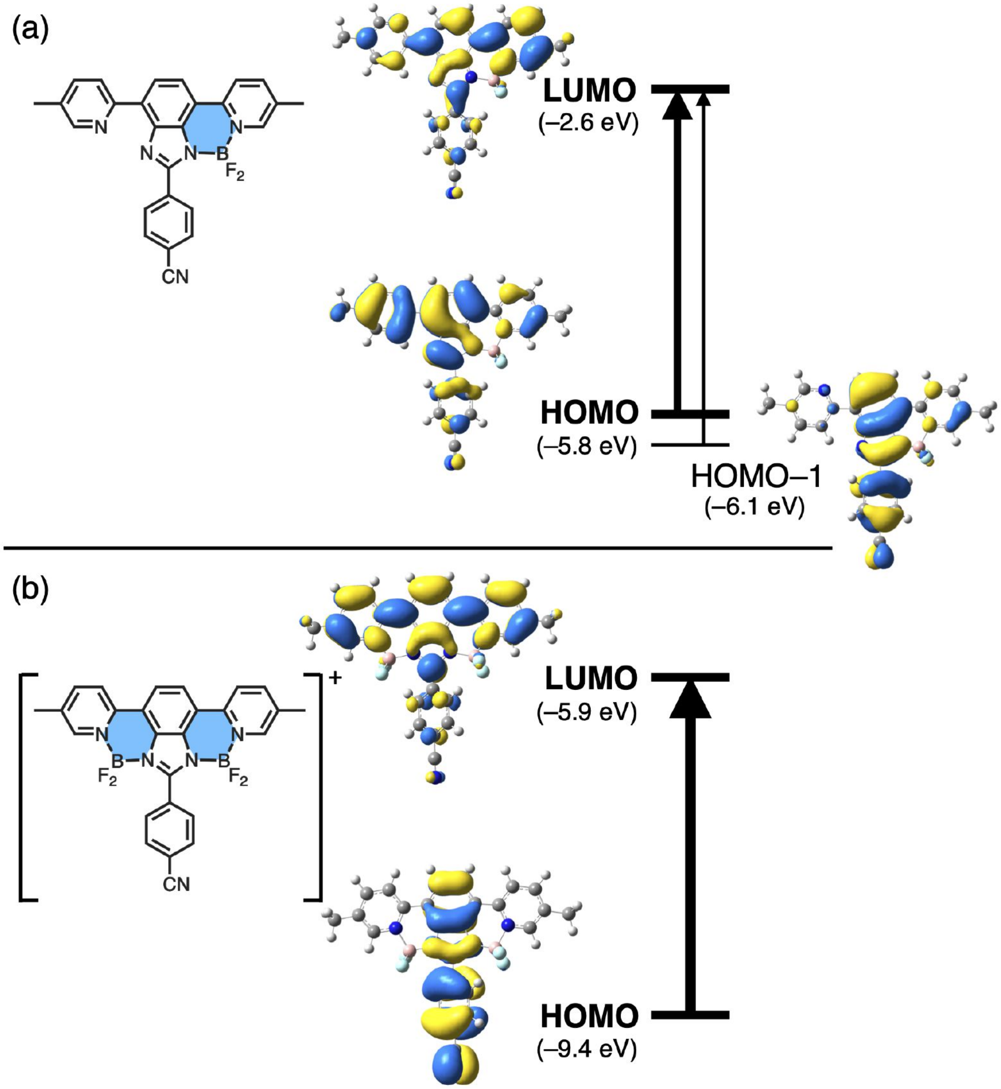

Supplementary Fig. 13. Frontier molecular orbitals (FMOs) involved in the transitions to the S_1 excited states of (a) **EliF-2b** and (b) **EliF-3b** examined by TD-DFT computational studies (B3LYP/6-31G(d) level). The thickness of the arrows corresponds to the relative contribution of individual FMO transitions leading to the S_1 states.

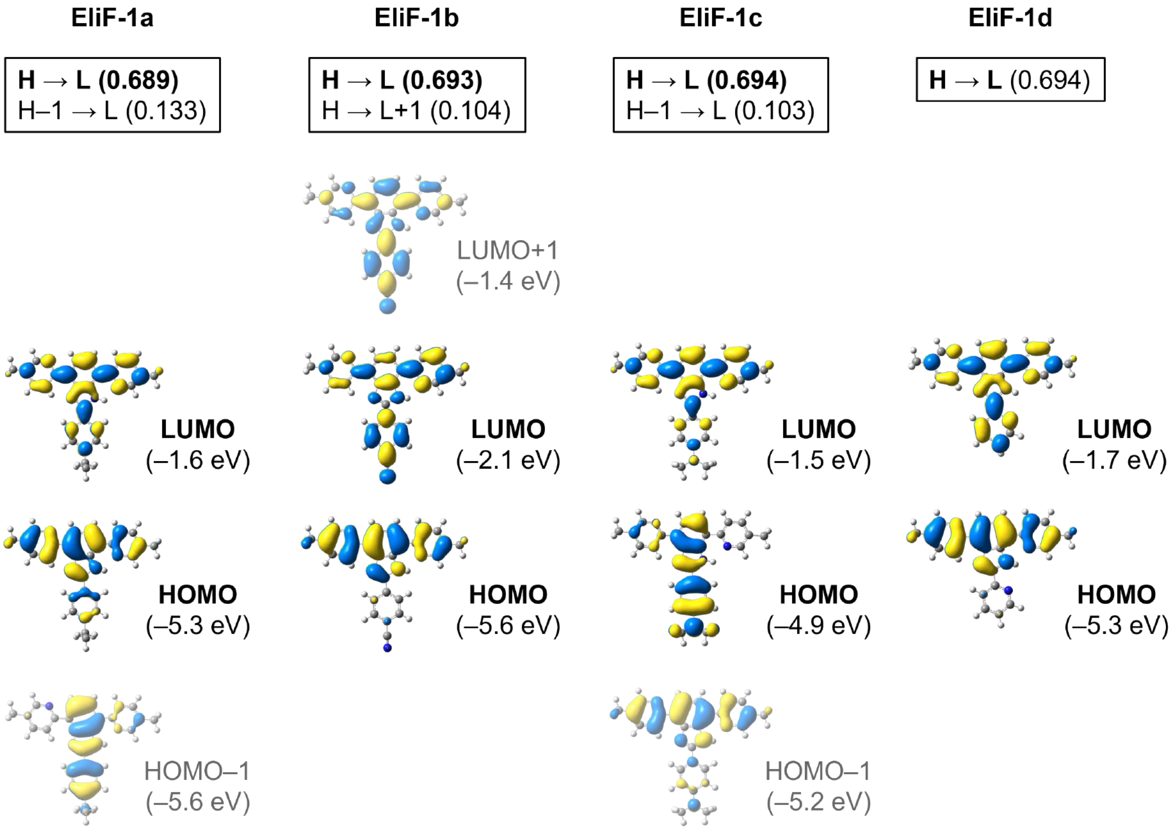

Supplementary Fig. 14. Frontier molecular orbitals (FMOs) involved in the transitions to the S_1 excited states of **EliF-1** series examined by TD-DFT computational studies (B3LYP/6-31G(d) level of theory). Numbers in parenthesis are CI (configuration interaction) expansion coefficients, the squared values of which correspond to the contribution of each FMO to the $S_0 \rightarrow S_1$ transition. Cartesian coordinates of geometry optimized structures are provided as Source Data file.

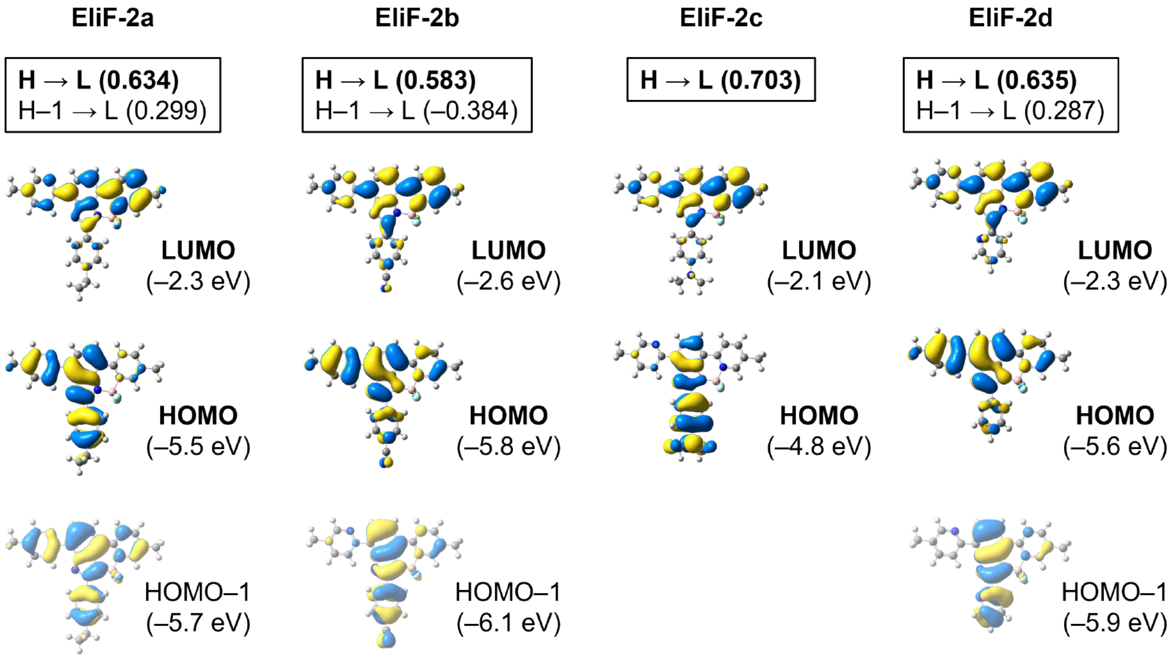

Supplementary Fig. 15. Frontier molecular orbitals (FMOs) involved in the transitions to the S_1 excited states of **EliF-2** series examined by TD-DFT computational studies (B3LYP/6-31G(d) level of theory). Numbers in parenthesis are CI (configuration interaction) expansion coefficients, the squared values of which correspond to the contribution of each FMO to the $S_0 \rightarrow S_1$ transition. Cartesian coordinates of geometry optimized structures are provided as Source Data file.

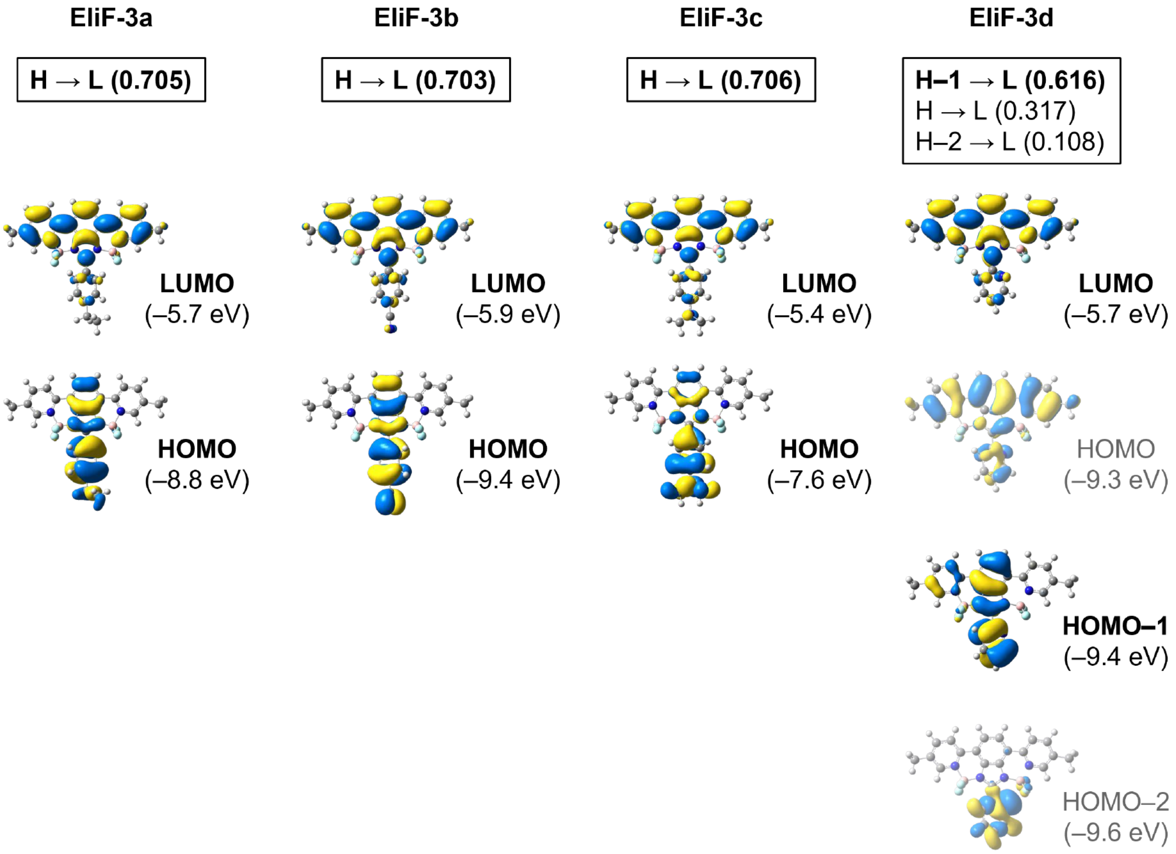

Supplementary Fig. 16. Frontier molecular orbitals (FMOs) involved in the transitions to the S_1 excited states of **EliF-3** series examined by TD-DFT computational studies (B3LYP/6-31G(d) level of theory). Numbers in parenthesis are CI (configuration interaction) expansion coefficients, the squared values of which correspond to the contribution of each FMO to the $S_0 \rightarrow S_1$ transition. Cartesian coordinates of geometry optimized structures are provided as Source Data file.

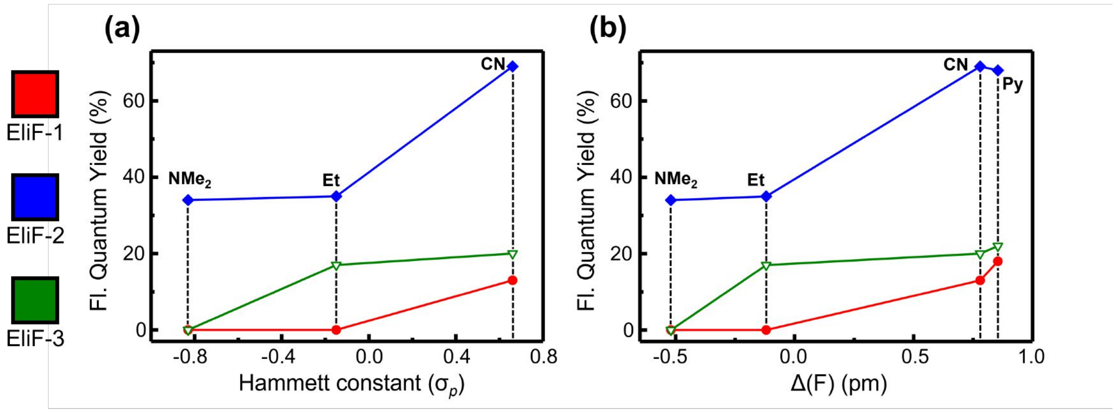

Supplementary Fig. 17. Plots of fluorescence quantum yield vs (a) Hammett constant,[11] and (b) Δ(F) (= $d_{\text{C–F}}$(Ph–F)–$d_{\text{C–F}}$(Ar–F)) value.[12,13]

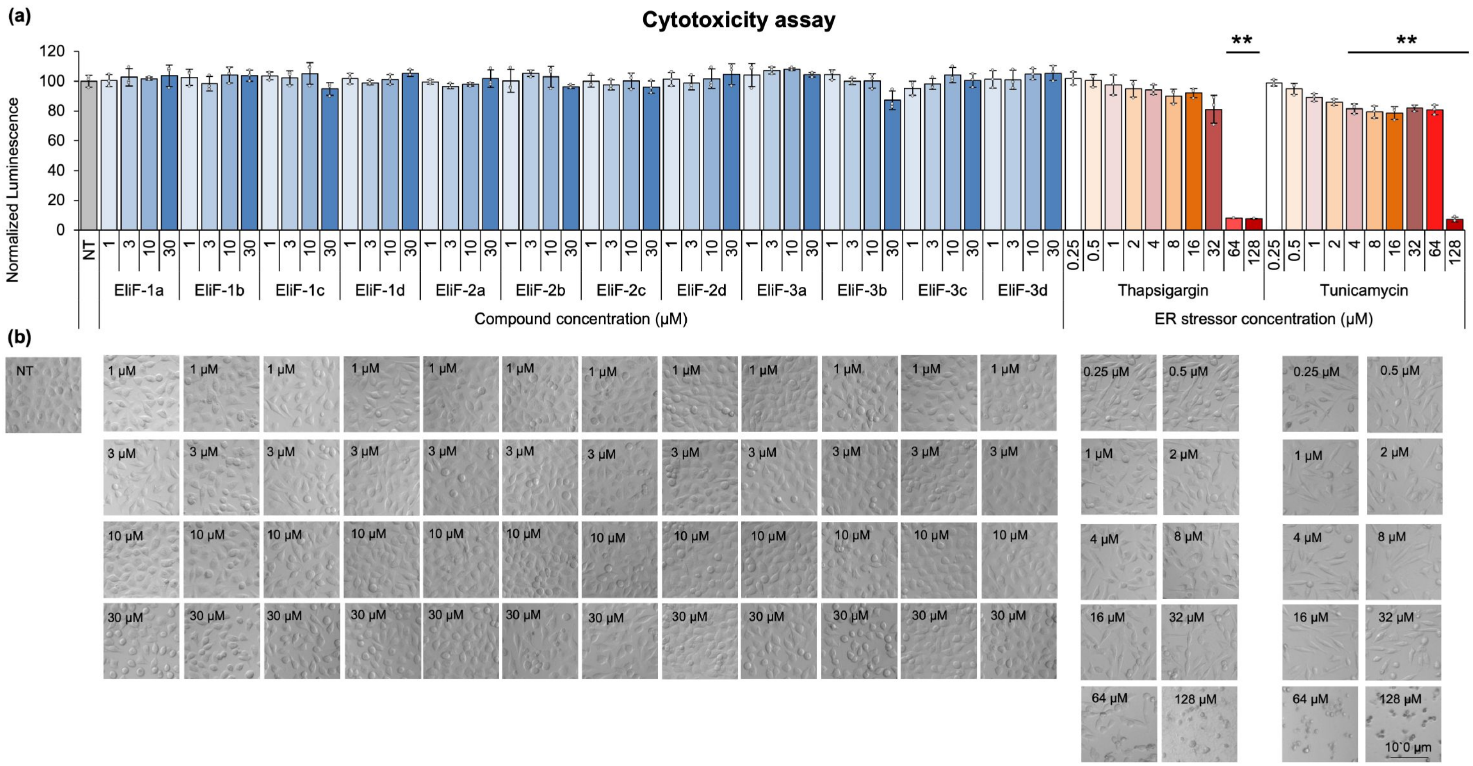

Supplementary Fig. 18. (a) Cellular toxicity effect of EliF probes measured by CellTiter-Glo assay. Cellular viability of HeLa cells was determined after 2 h of incubation with each EliF probe, and 12 h of incubation with ER stressors (thapsigargin and tunicamycin) according to the manufacturer's protocol; $n = 3$ biologically independent cells were examined, and data are presented as mean values ± SD (** $p < 0.01$ for Thapsigargin 64 μM ($p = 0.006$), 128 μM ($p = 0.006$); Tunicamycin 4 μM ($p = 0.0035$), 8 μM ($p = 0.0031$), 16 μM ($p = 0.0030$), 32 μM ($p = 0.0052$), 64 μM ($p = 0.0030$), 128 μM ($p = 0.0001$); two-tailed paired t-test). (b) Shown below are representative bright field images of the cells.

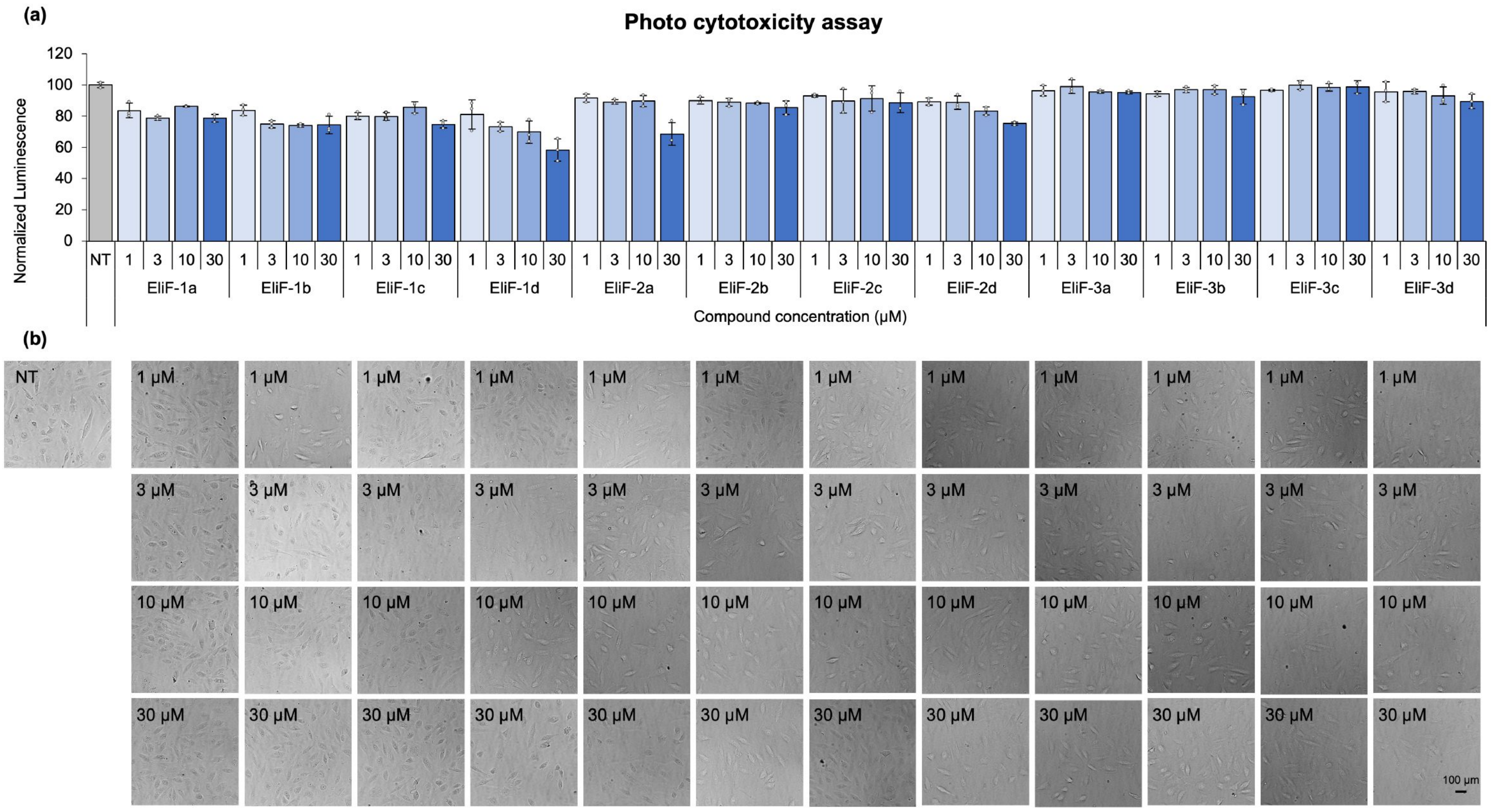

Supplementary Fig. 19. (a) Cellular toxicity effect of EliF probes measured by CellTiter-Glo assay. Cellular viability of HeLa cells was determined by incubation with each EliF probe for 30 min followed by 350 nm UV irradiation for 10 s according to the manufacturer's protocol; $n = 3$ biologically independent cells were examined, and data are presented as mean values ± SD. (b) Shown below are representative bright field images of the cells.

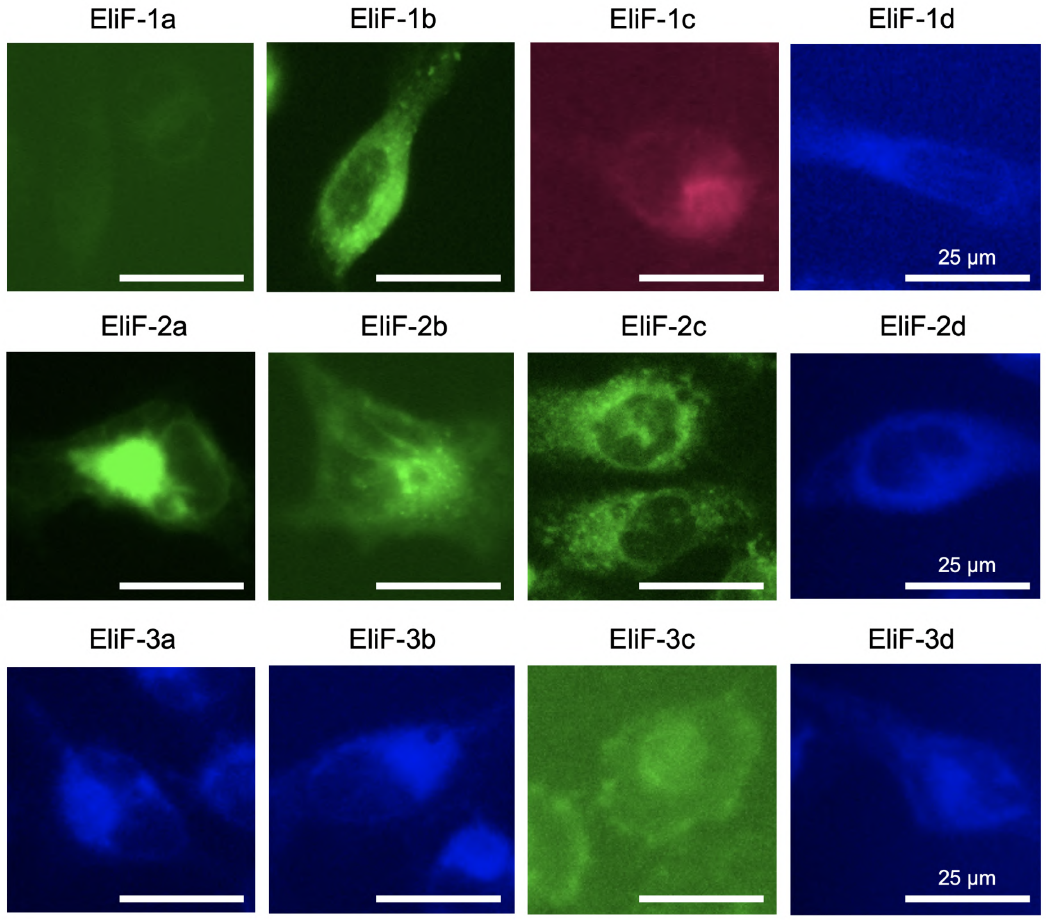

Supplementary Fig. 20. Representative fluorescence images of live cells treated with EliF probes. Blue, green, and red colored images were obtained using DAPI (λ_{exc} = 350/50 nm; λ_{em} = 460/50 nm), F1 (λ_{exc} = 350/50 nm; λ_{em} = 527/30 nm), and F2 (λ_{exc} = 350/50 nm; λ_{em} = 585/40 nm) filter-set, respectively. Imaging experiments were conducted on at least three independent biological replicates; all replicates produced similar results.

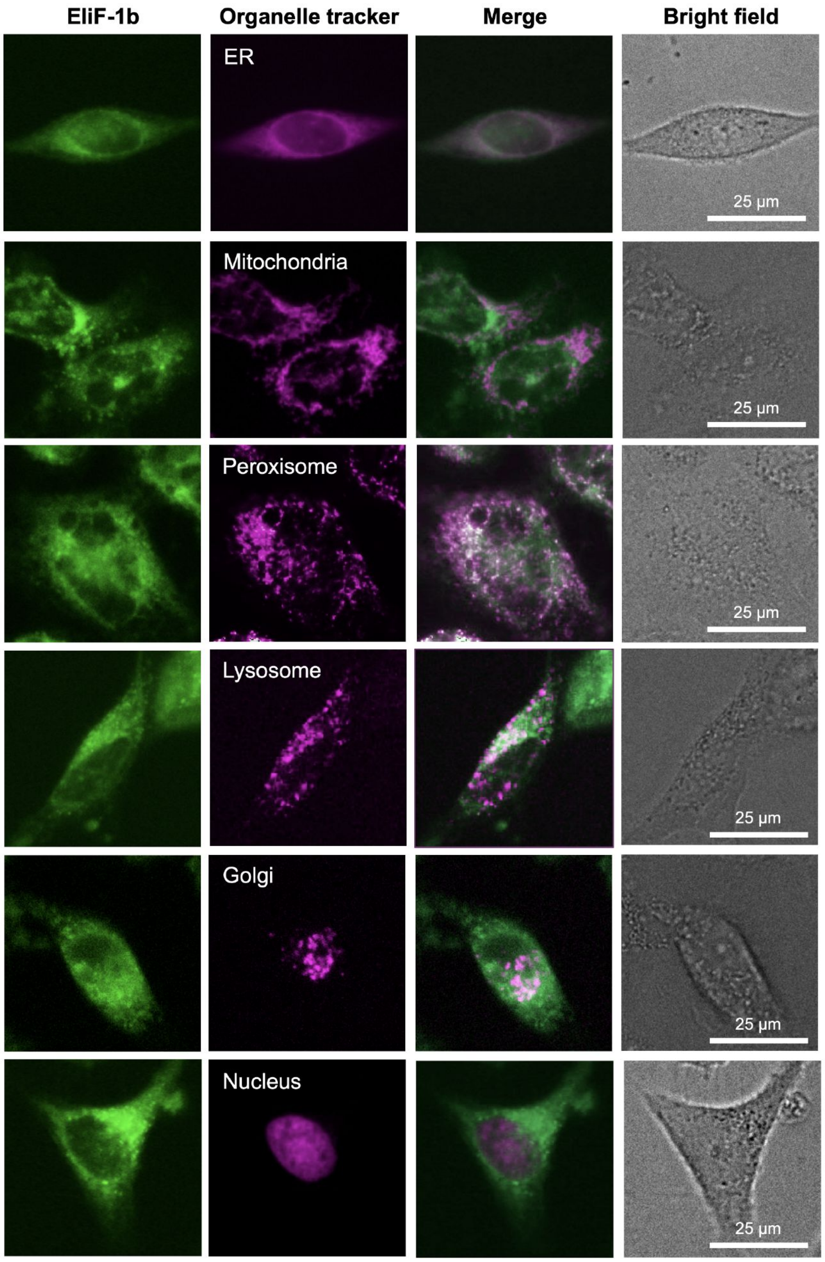

Supplementary Fig. 21. Fluorescence colocalization images of **EliF-1b** and commercial organelle trackers (CellLight^{TM} ER-RFP, CellLight^{TM} Mitochondria-RFP, CellLight^{TM} Peroxisome-GFP, CellLight^{TM} Lysosome-RFP, CellLight^{TM} Golgi-RFP, and NUCLEAR-ID Red). Fluorescence images were obtained using F1 (λ_{exc} = 350/50 nm; λ_{em} = 527/30 nm) and RHOD (λ_{exc} = 546/10 nm; λ_{em} = 585/40 nm) filter-set for **EliF-1b** and organelle trackers, respectively, except for peroxisome-GFP (FITC fiter-set: λ_{exc} = 480/40 nm; λ_{em} = 527/30 nm) and NUCLEAR-ID Red (Y5 filter-set: λ_{exc} = 620/60 nm; λ_{em} = 700/75 nm). Peroxisome-GFP signal was presented as pseudo color red to generate a clear merged image. Imaging experiments were conducted on at least three independent biological replicates; all replicates produced similar results.

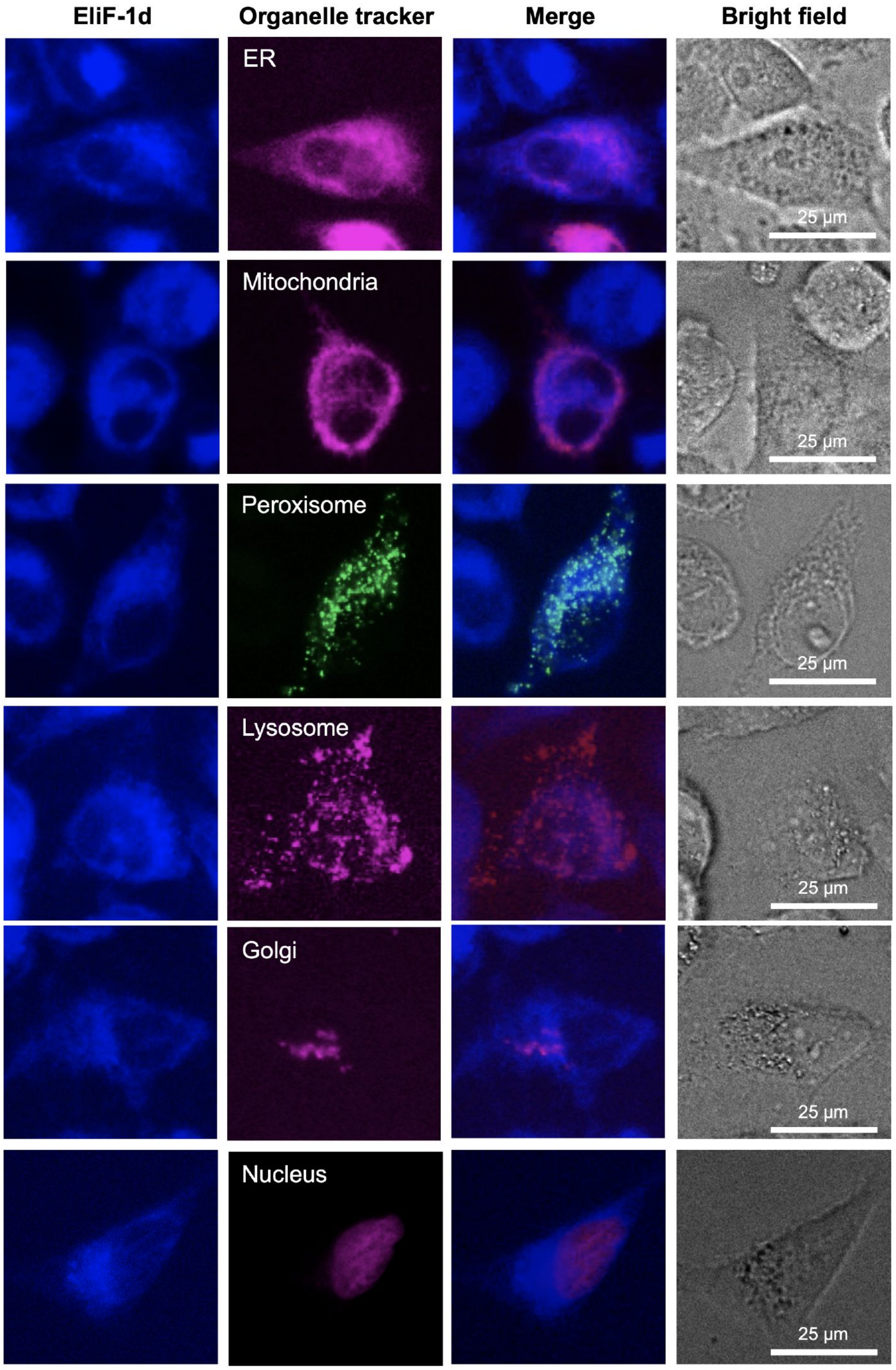

Supplementary Fig. 22. Fluorescence colocalization images of **EliF-1d** and commercial organelle trackers (CellLight^{TM} ER-RFP, CellLight^{TM} Mitochondria-RFP, CellLight^{TM} Peroxisome-GFP, CellLight^{TM} Lysosome-RFP, CellLight^{TM} Golgi-RFP, and NUCLEAR-ID Red). Fluorescence images were obtained using DAPI (λ_{exc} = 350/50 nm; λ_{em} = 460/50 nm) and RHOD (λ_{exc} = 546/10 nm; λ_{em} = 585/40 nm) filter-set for **EliF-1d** and organelle trackers, respectively, except for peroxisome-GFP (FITC fiter-set: λ_{exc} = 480/40 nm; λ_{em} = 527/30 nm) and NUCLEAR-ID Red (Y5 filter-set: λ_{exc} = 620/60 nm; λ_{em} = 700/75 nm). Imaging experiments were conducted on at least three independent biological replicates; all replicates produced similar results.

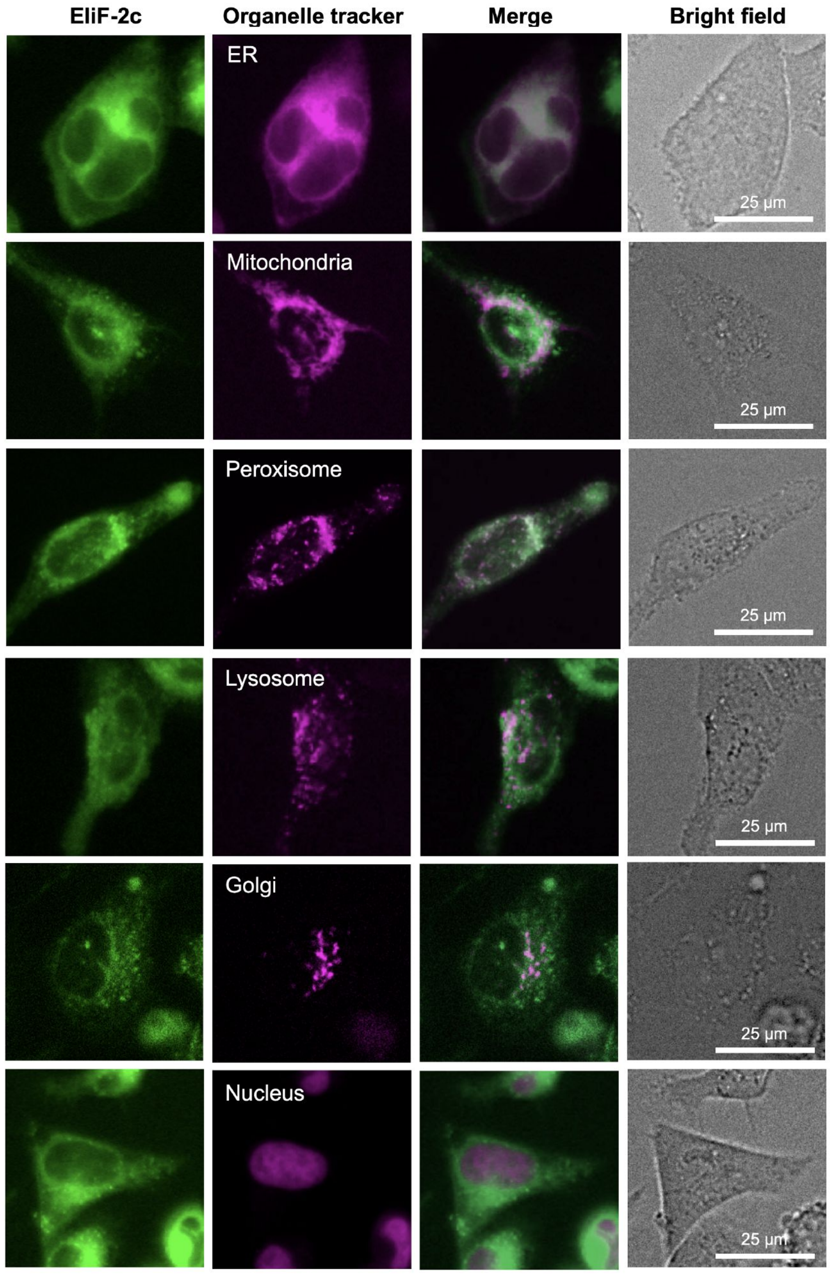

Supplementary Fig. 23. Fluorescence colocalization images of **EliF-2c** and commercial organelle trackers (CellLight^{TM} ER-RFP, CellLight^{TM} Mitochondria-RFP, CellLight^{TM} Peroxisome-GFP, CellLight^{TM} Lysosome-RFP, CellLight^{TM} Golgi-RFP, and NUCLEAR-ID Red). Fluorescence images were obtained using F1 (λ_{exc} = 350/50 nm; λ_{em} = 527/30 nm) and RHOD (λ_{exc} = 546/10 nm; λ_{em} = 585/40 nm) filter-set for **EliF-2c** and organelle trackers, respectively, except for peroxisome-GFP (FITC fiter-set: λ_{exc} = 480/40 nm; λ_{em} = 527/30 nm) and NUCLEAR-ID Red (Y5 filter-set: λ_{exc} = 620/60 nm; λ_{em} = 700/75 nm). Peroxisome-GFP signal was presented as pseudo color red to generate a clear merged image. Imaging experiments were conducted on at least three independent biological replicates; all replicates produced similar results.

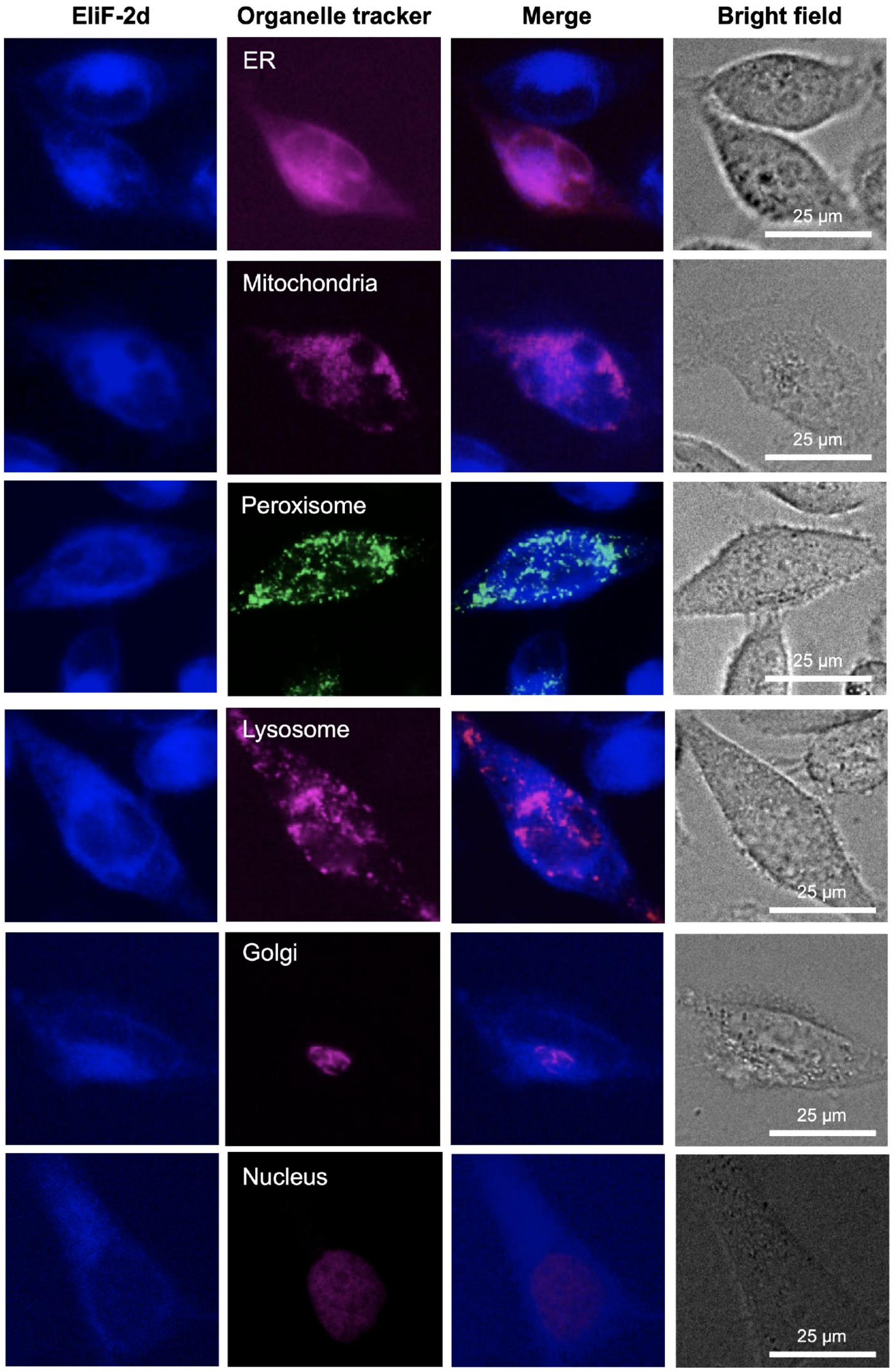

Supplementary Fig. 24. Fluorescence colocalization images of **EliF-2d** and commercial organelle trackers (CellLight^{TM} ER-RFP, CellLight^{TM} Mitochondria-RFP, CellLight^{TM} Peroxisome-GFP, CellLight^{TM} Lysosome-RFP, CellLight^{TM} Golgi-RFP, and NUCLEAR-ID Red). Fluorescence images were obtained using DAPI (λ_{exc} = 350/50 nm; λ_{em} = 460/50 nm) and RHOD (λ_{exc} = 546/10 nm; λ_{em} = 585/40 nm) filter-set for **EliF-2d** and organelle trackers, respectively, except for peroxisome-GFP (FITC fiter-set: λ_{exc} = 480/40 nm; λ_{em} = 527/30 nm) and NUCLEAR-ID Red (Y5 filter-set: λ_{exc} = 620/60 nm; λ_{em} = 700/75 nm). Imaging experiments were conducted on at least three independent biological replicates; all replicates produced similar results.

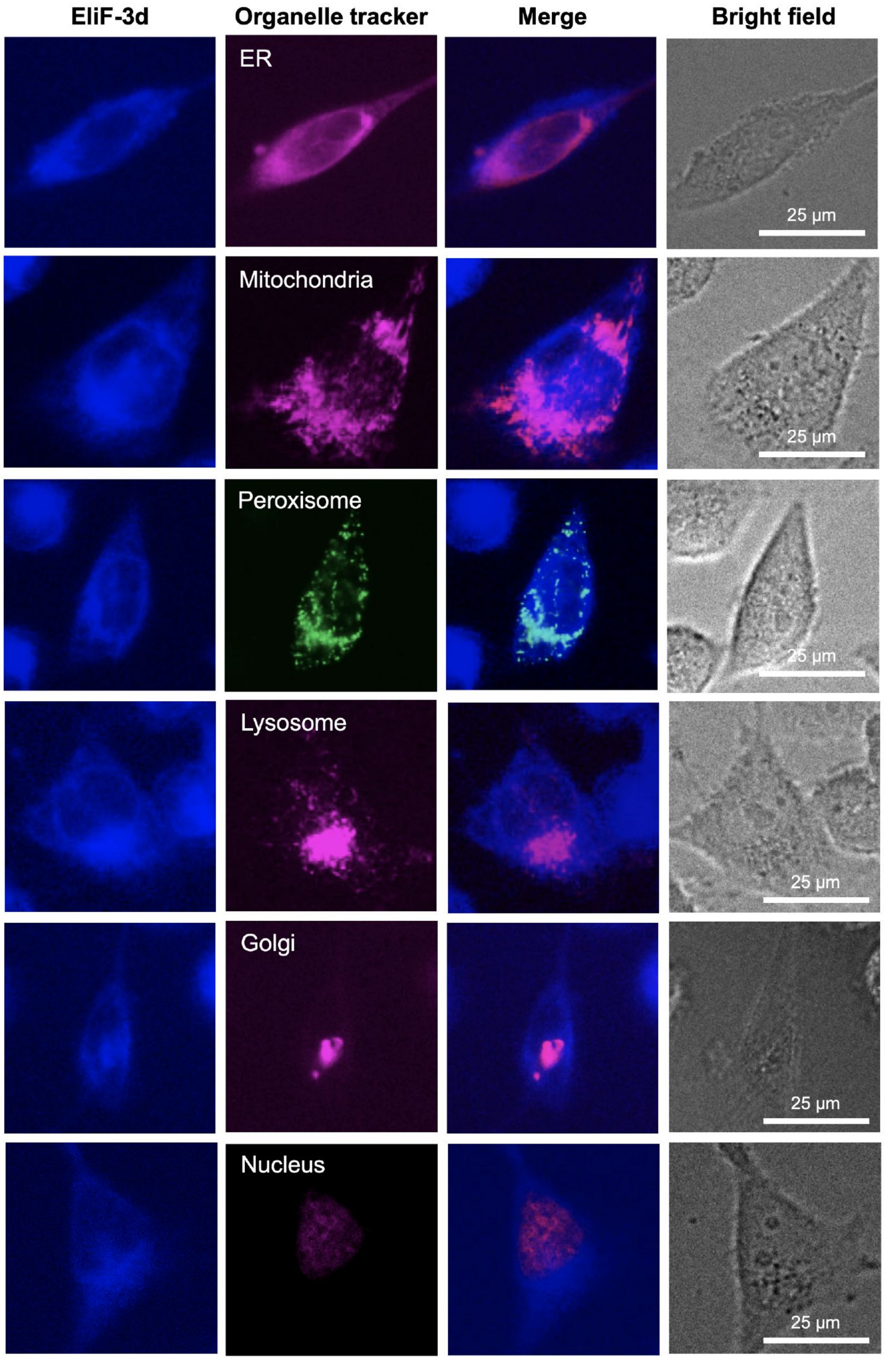

Supplementary Fig. 25. Fluorescence colocalization images of **EliF-3d** and commercial organelle trackers (CellLight^{TM} ER-RFP, CellLight^{TM} Mitochondria-RFP, CellLight^{TM} Peroxisome-GFP, CellLight^{TM} Lysosome-RFP, CellLight^{TM} Golgi-RFP, and NUCLEAR-ID Red). Fluorescence images were obtained using DAPI (λ_{exc} = 350/50 nm; λ_{em} = 460/50 nm) and RHOD (λ_{exc} = 546/10 nm; λ_{em} = 585/40 nm) filter-set for **EliF-3d** and organelle trackers, respectively, except for peroxisome-GFP (FITC fiter-set: λ_{exc} = 480/40 nm; λ_{em} = 527/30 nm) and NUCLEAR-ID Red (Y5 filter-set: λ_{exc} = 620/60 nm; λ_{em} = 700/75 nm). Imaging experiments were conducted on at least three independent biological replicates; all replicates produced similar results.

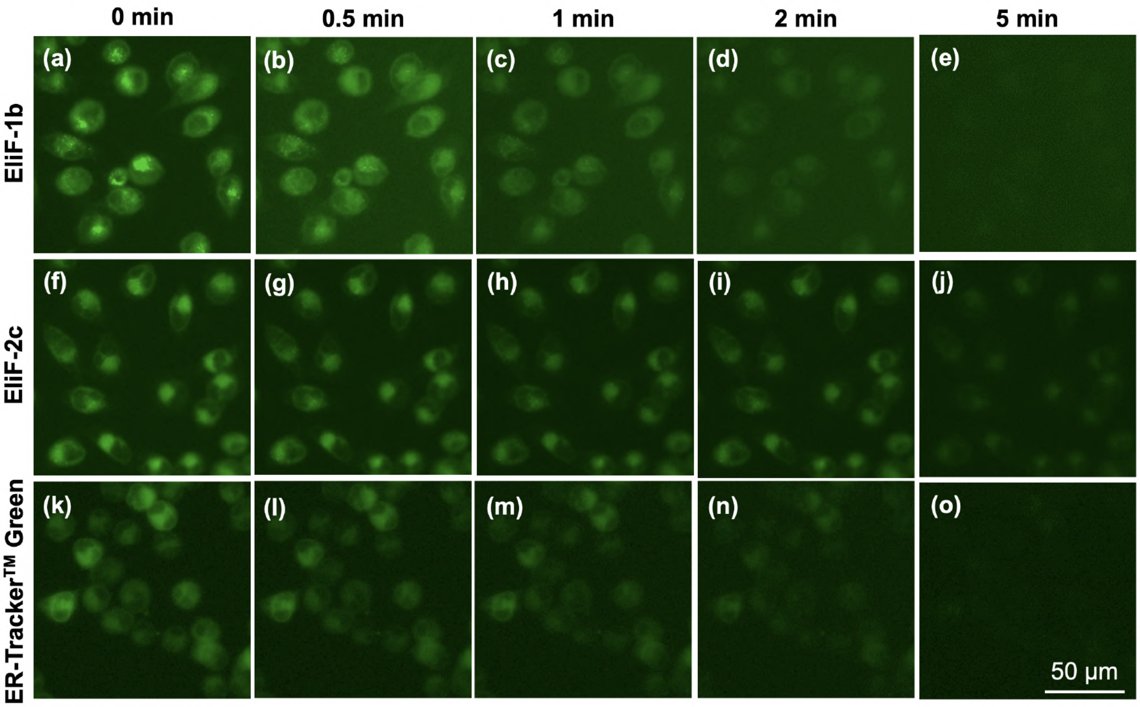

Supplementary Fig. 26. Comparison of the photostability of EliF probes and commercial ER-Tracker™ Green. Fluorescence live cell images of HeLa cells stained with (a–e) **EliF-1b**, (f–j) **EliF-2c**, and (k–o) ER-Tracker™ Green after photo-irradiation for 0 to 5 min. Fluorescence images were obtained using F1 (λ_{exc} = 350/50 nm; λ_{em} = 527/30 nm) and FITC (λ_{exc} = 480/40 nm; λ_{em} = 527/30 nm) filter-set for EliF probes and ER-Tracker™ Green, respectively. Imaging experiments were conducted on at least three independent biological replicates; all replicates produced similar results.

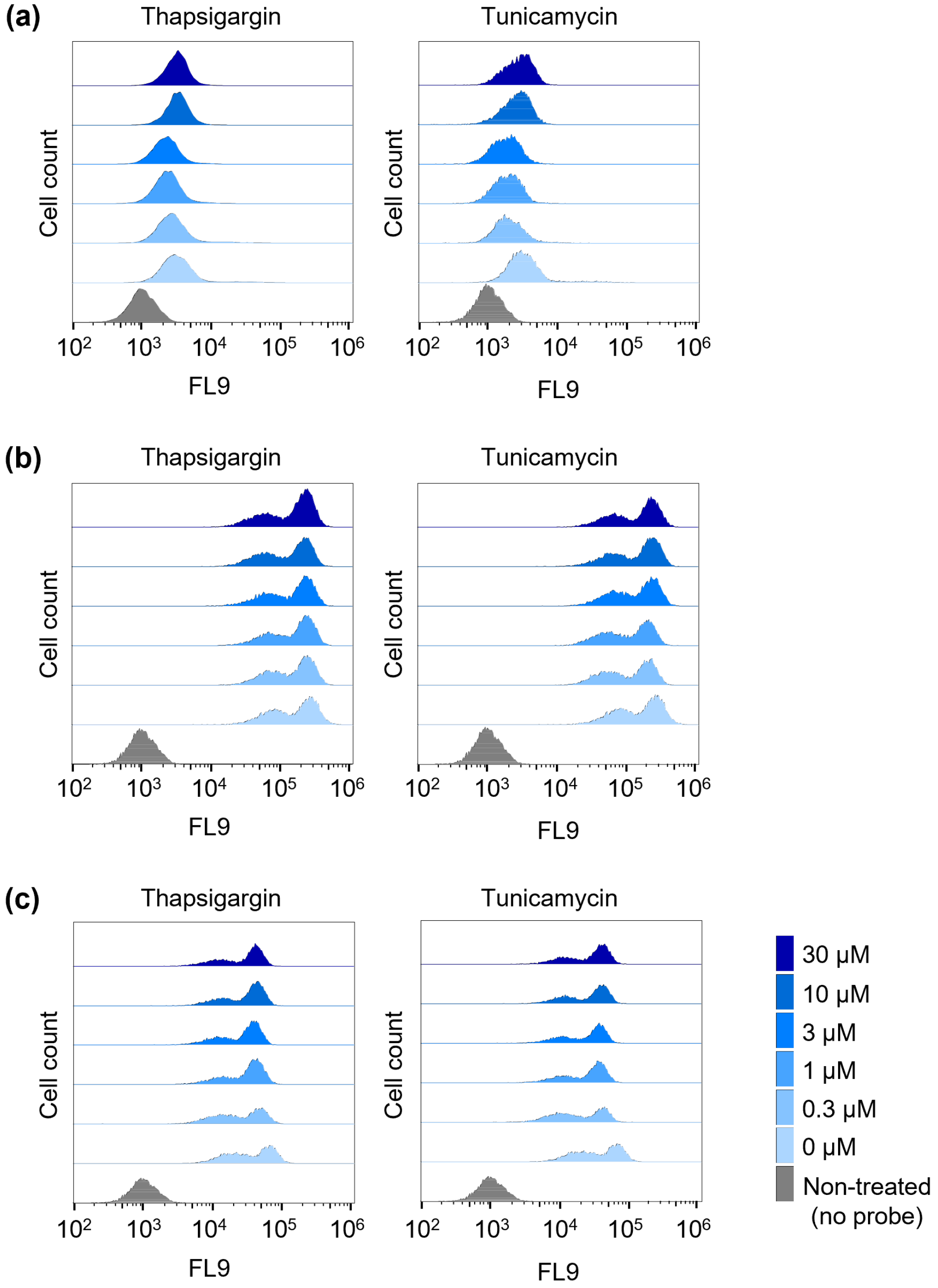

Supplementary Fig. 27. ER stress responses of **EliF-1d**, **EliF-2d** and **EliF-3d**. Flow cytometry analysis of (a) **EliF-1d** (10 μM), (b) **EliF-2d** (10 μM), and (c) **EliF-3d** (10 μM) in ER stress induced HeLa cells. ER stress was chemically induced by increasing concentrations (0–30 μM) of either thapsigargin or tunicamycin preincubation for 12 h, and monitored by cell population shifts in flow cytometry using FL9 filter (λ_{exc} = 405 nm; λ_{em} = 450/40 nm). Based on the mean fluorescence intensity, **EliF-1d** showed 0.91- and 0.75-fold increment with TG and TN, respectively (concentration = 30 μM). **EliF-2d** showed 0.75- and 0.89-fold increment with TG and TN, respectively (concentration = 30 μM). **EliF-3d** showed 0.80- and 1.10-fold increment with TG and TN, respectively (concentration = 30 μM).

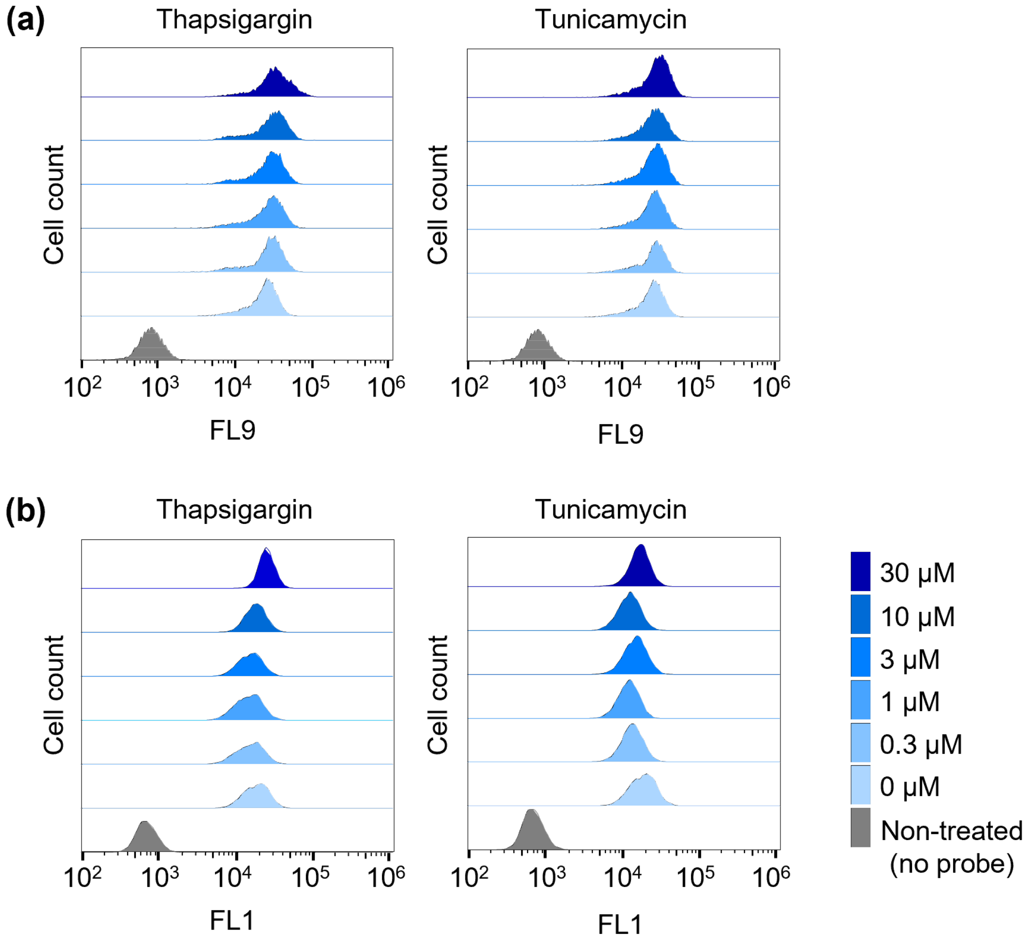

Supplementary Fig. 28. ER stress responses of ER-Tracker^{TM} Blue-White and ER-Tracker^{TM} Green. Flow cytometry analysis of (a) ER-Tracker^{TM} Blue-White (10 μM) and (b) ER-Tracker^{TM} Green (10 μM) in ER stress induced HeLa cells. ER stress was chemically induced by increasing concentrations (0–30 μM) of either thapsigargin or tunicamycin preincubation for 12 h, and monitored by cell population shifts in flow cytometry using FL9 filter (λ_{exc} = 405 nm; λ_{em} = 450/40 nm) for ER-Tracker^{TM} Blue-White, and FL1 filter (λ_{exc} = 488 nm; λ_{em} = 525/40 nm) for ER-Tracker^{TM} Green. Based on the mean fluorescence intensity, ER-Tracker^{TM} Blue-White showed 1.21- and 1.11-fold increment with TG and TN, respectively (concentration = 30 μM). ER-Tracker^{TM} Green showed 1.37- and 1.34-fold increment with TG and TN, respectively (concentration = 30 μM).

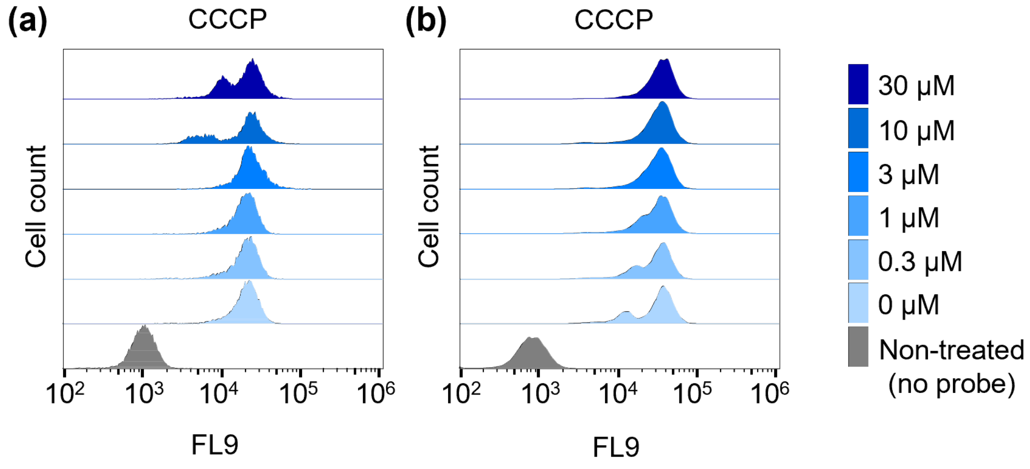

Supplementary Fig. 29. Mitochondrial membrane depolarization responses of **EliF-1b** and **EliF-2c**. Flow cytometry analysis of (a) **EliF-1b** (10 μM) and (b) **EliF-2c** (10 μM) in mitochondrial membrane depolarization induced HeLa cells. Mitochondrial membrane depolarization was chemically induced by increasing concentrations (0–30 μM) of CCCP preincubation for 4 h, and monitored by cell population shifts in flow cytometry using FL10 filter (λ_{exc} = 405 nm; λ_{em} = 550/40 nm). Based on the mean fluorescence intensity, **EliF-1b** showed 0.90- and **EliF-2c** showed 1.18-fold increament with CCCP, respectively (concentration = 30 μM).

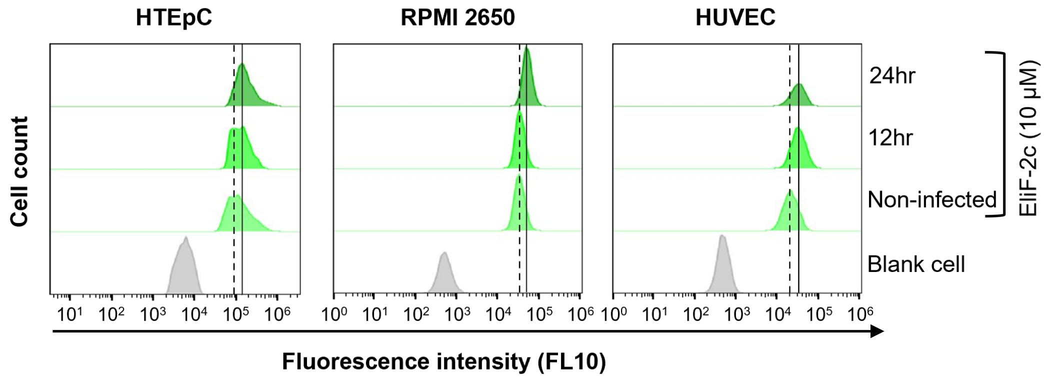

Supplementary Fig. 30. Flow cytometry analysis of **EliF-2c** (10 μM) in H1N1 virus infected HTEpC, RPMI 2650, and HUVEC cells using FL10 filter (λ_{exc} = 405 nm; λ_{em} = 550/40 nm).

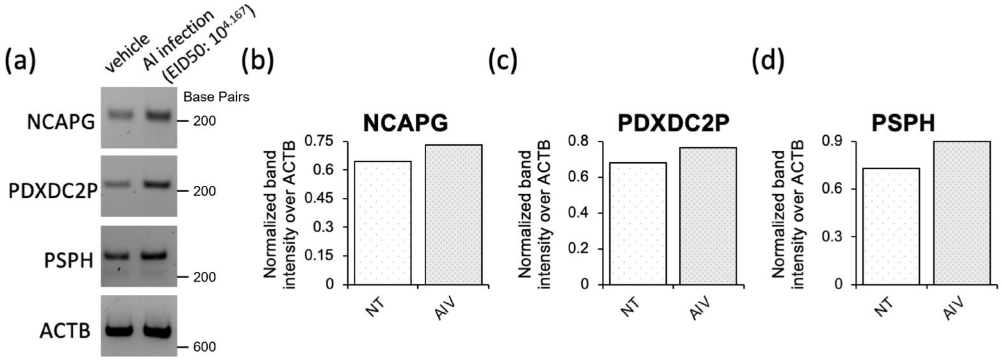

Supplementary Fig. 31. (a) RT-PCR analysis of major target proteins (NCAPG, PDXDC2P, PSPH) upon H1N1 virus infection. Increased band intensities of (b) NCAPG, (c) PDXDC2P, and (d) PSPH upon AI infection. The band intensities are normalized against ACTB, which was used as control. Source data are provided as a Source Data file.

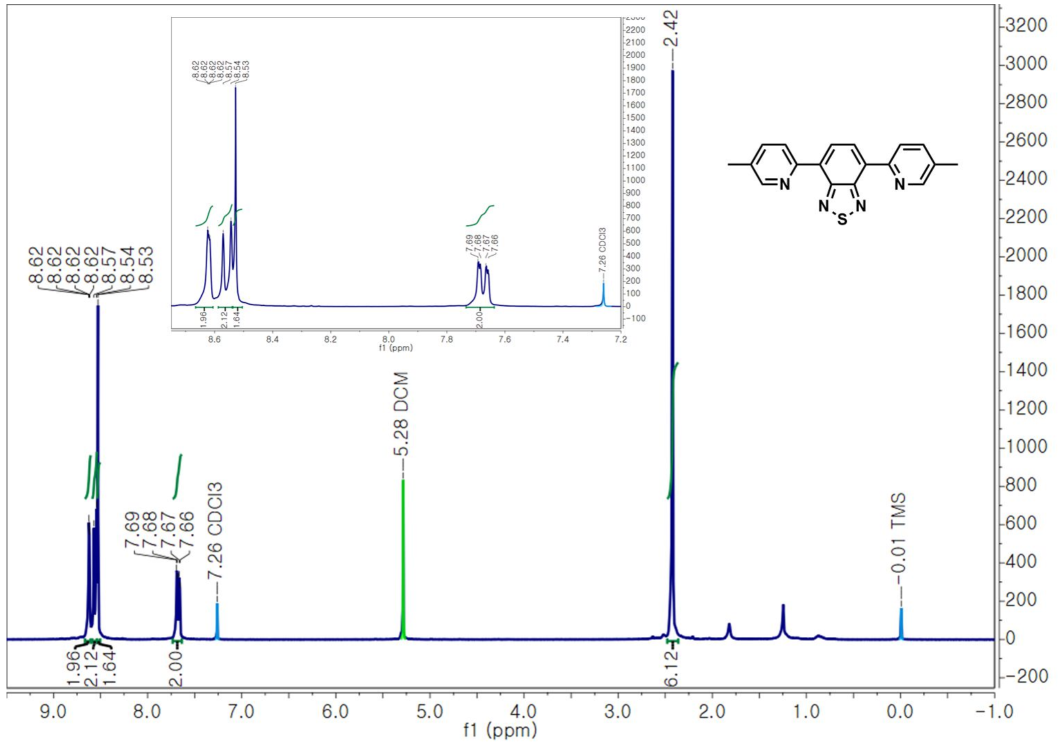

Supplementary Fig. 32. ^{1}H NMR (300 MHz) spectrum of **1** in $CDCl_3$ (T = 298 K).

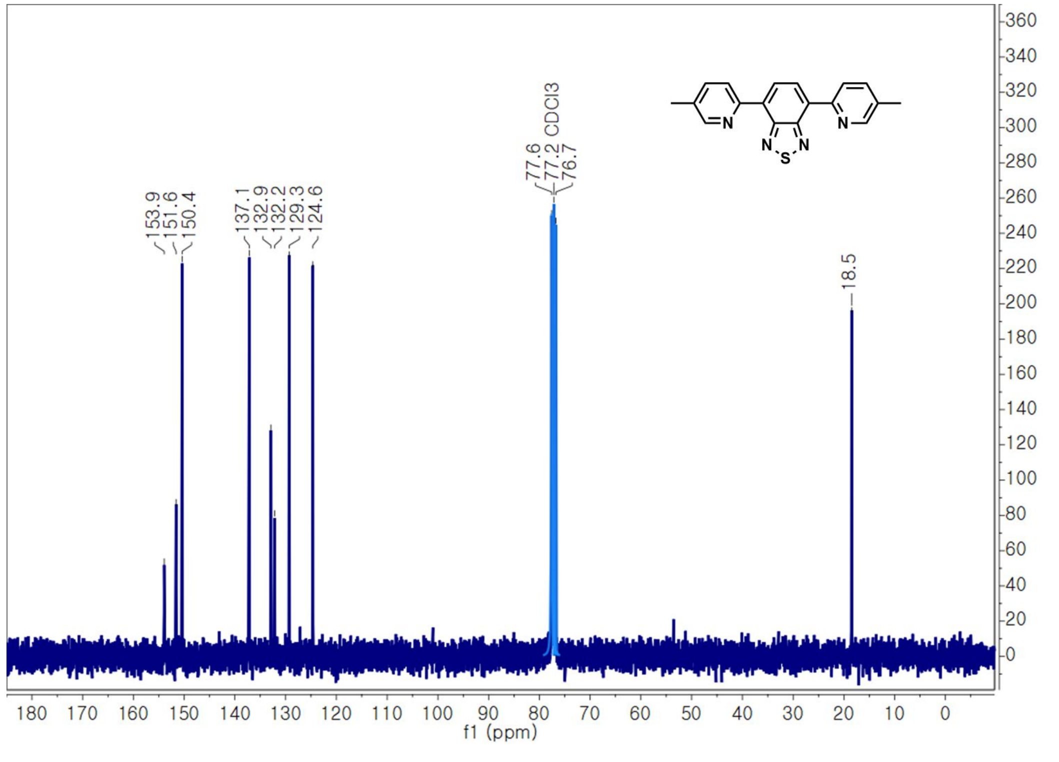

Supplementary Fig. 33. ^{13}C NMR (75 MHz) spectrum of **1** in $CDCl_3$ (T = 298 K).

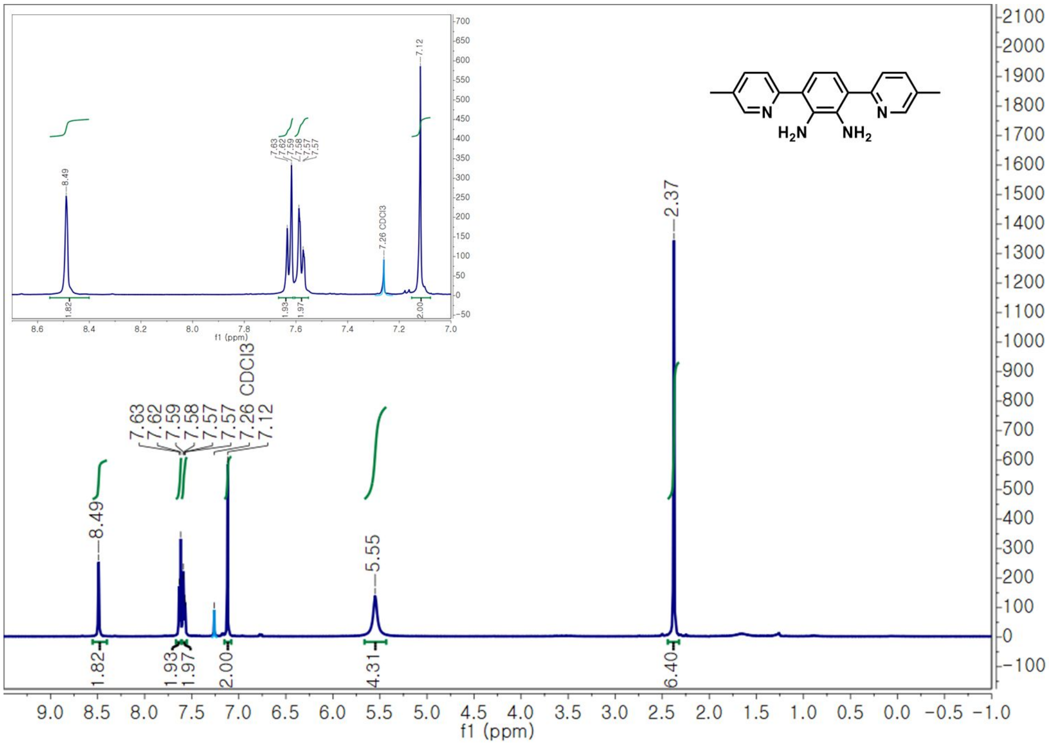

Supplementary Fig. 34. ^{1}H NMR (500 MHz) spectrum of **2** in $CDCl_3$ (T = 298 K).

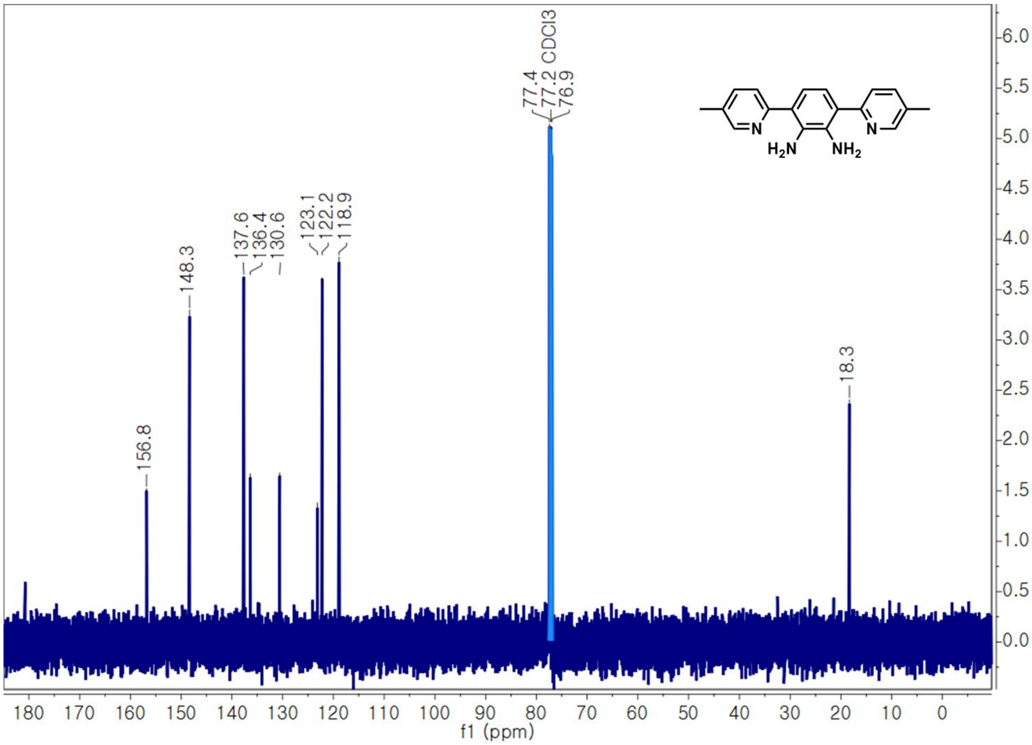

Supplementary Fig. 35. ^{13}C NMR (125 MHz) spectrum of **2** in $CDCl_3$ (T = 298 K).

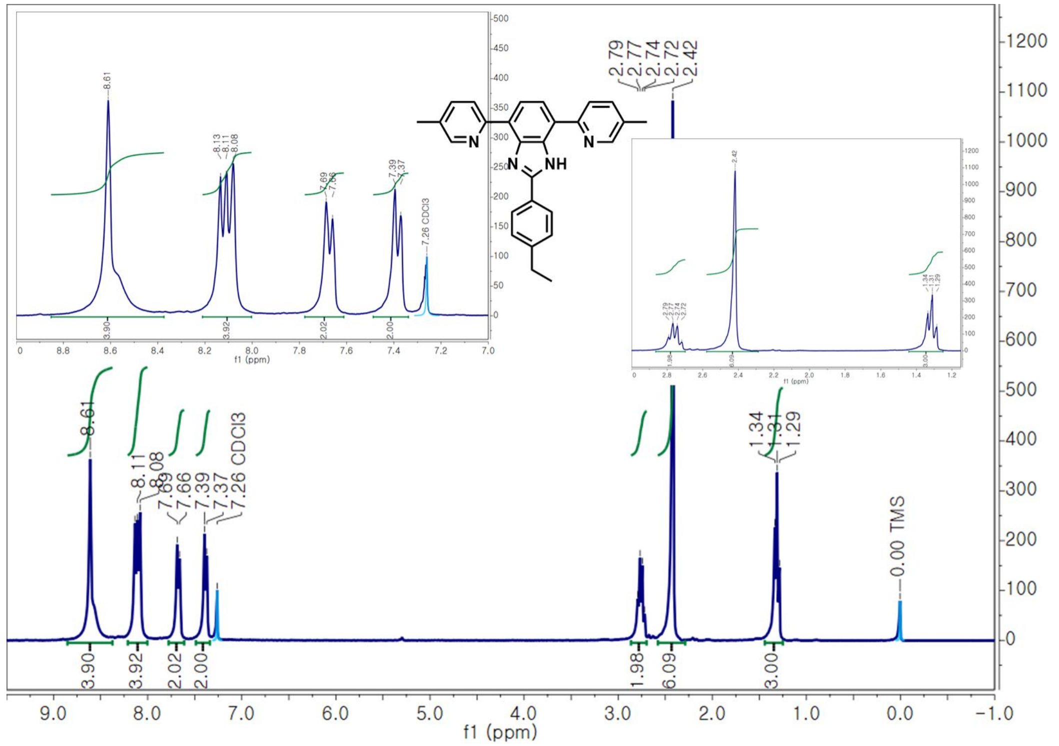

Supplementary Fig. 36. ^{1}H NMR (300 MHz) spectrum of **EliF-1a** in $CDCl_3$ (T = 298 K).

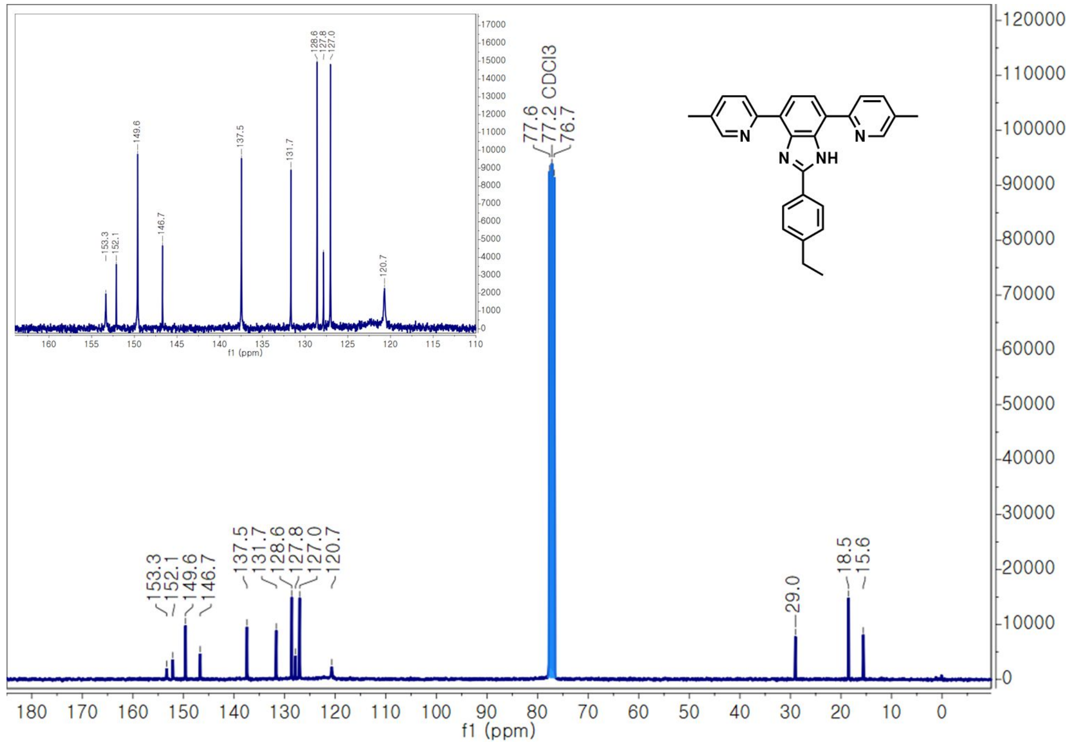

Supplementary Fig. 37. ^{13}C NMR (75 MHz) spectrum of **EliF-1a** in $CDCl_3$ (T = 298 K).

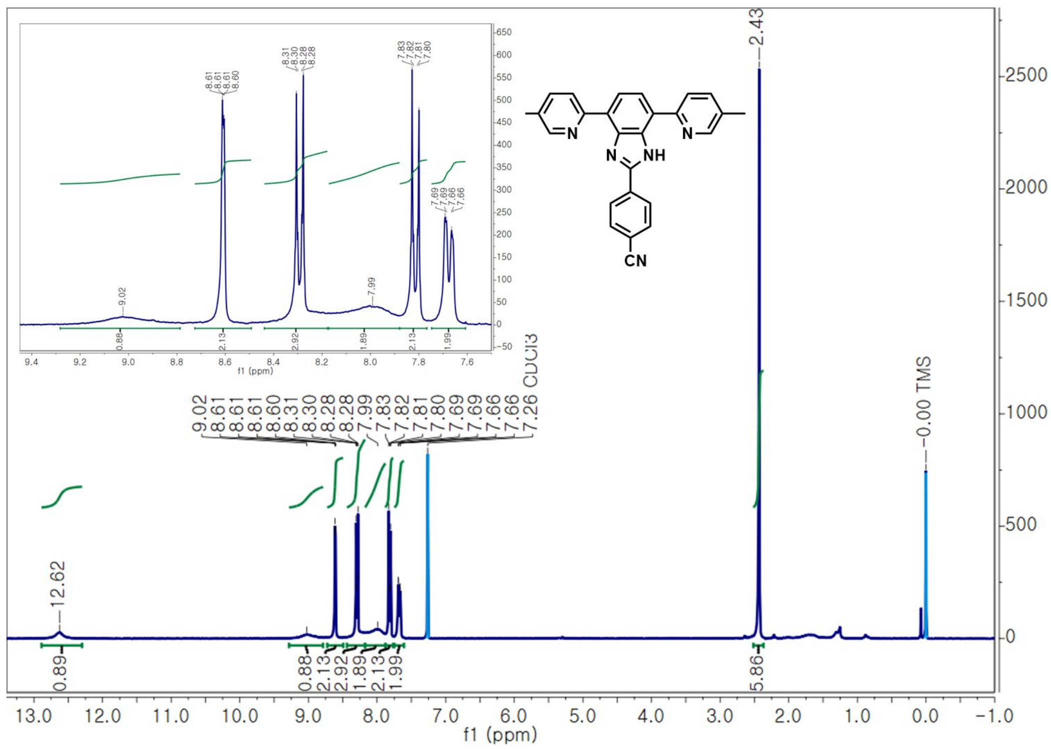

Supplementary Fig. 38. ^{1}H NMR (300 MHz) spectrum of **EliF-1b** in $CDCl_3$ (T = 298 K).

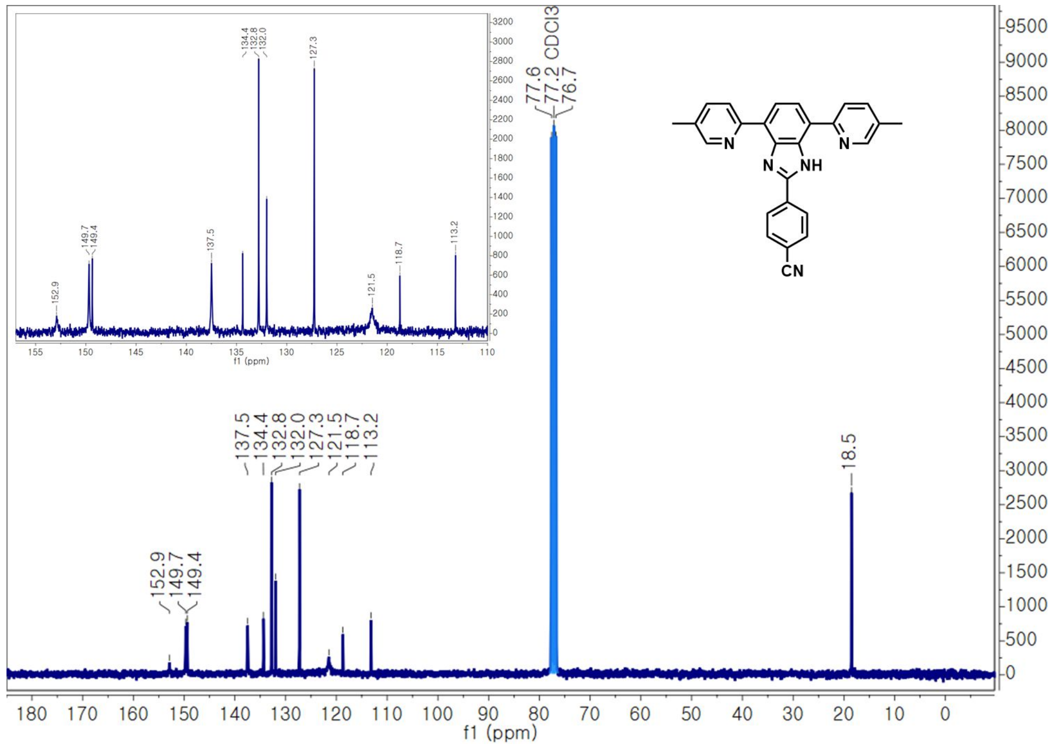

Supplementary Fig. 39. ^{13}C NMR (75 MHz) spectrum of **EliF-1b** in $CDCl_3$ (T = 298 K).

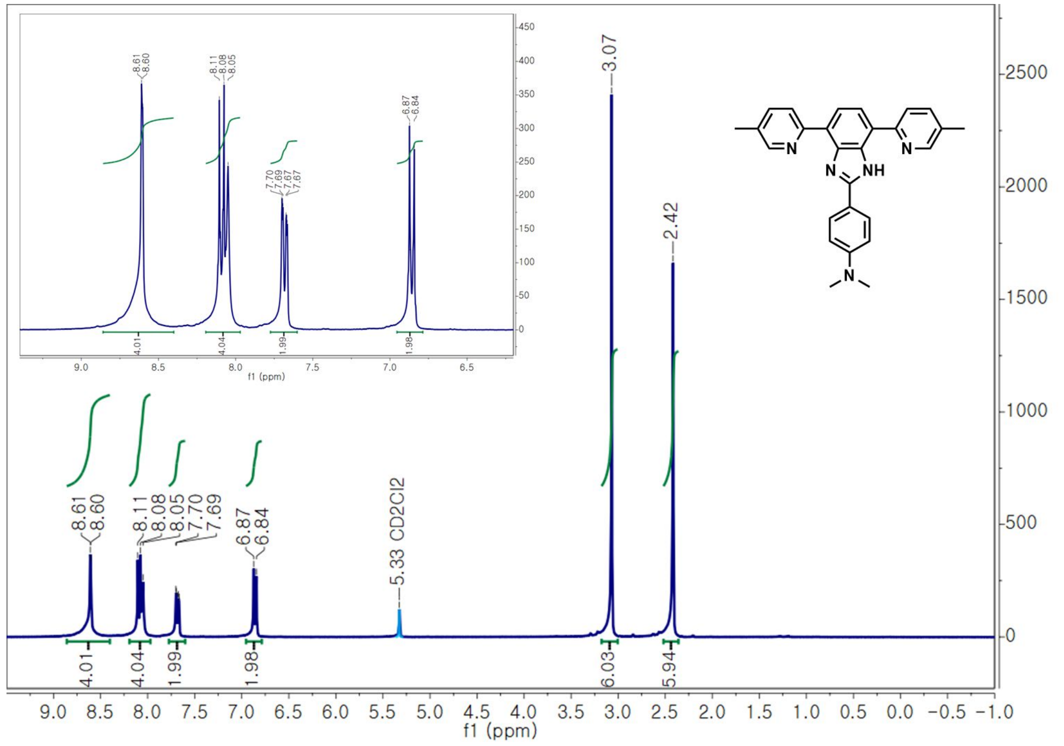

Supplementary Fig. 40. ^{1}H NMR (300 MHz) spectrum of **EliF-1c** in CD_2Cl_2 (T = 298 K).

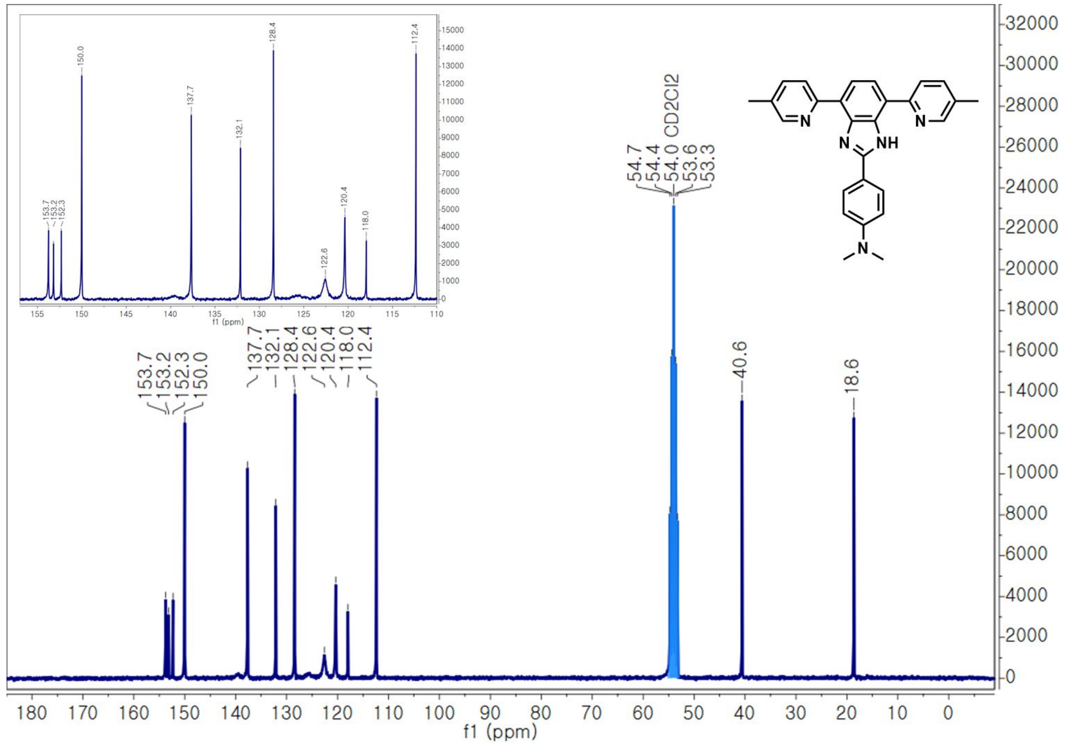

Supplementary Fig. 41. ^{13}C NMR (75 MHz) spectrum of **EliF-1c** in CD_2Cl_2 (T = 298 K).

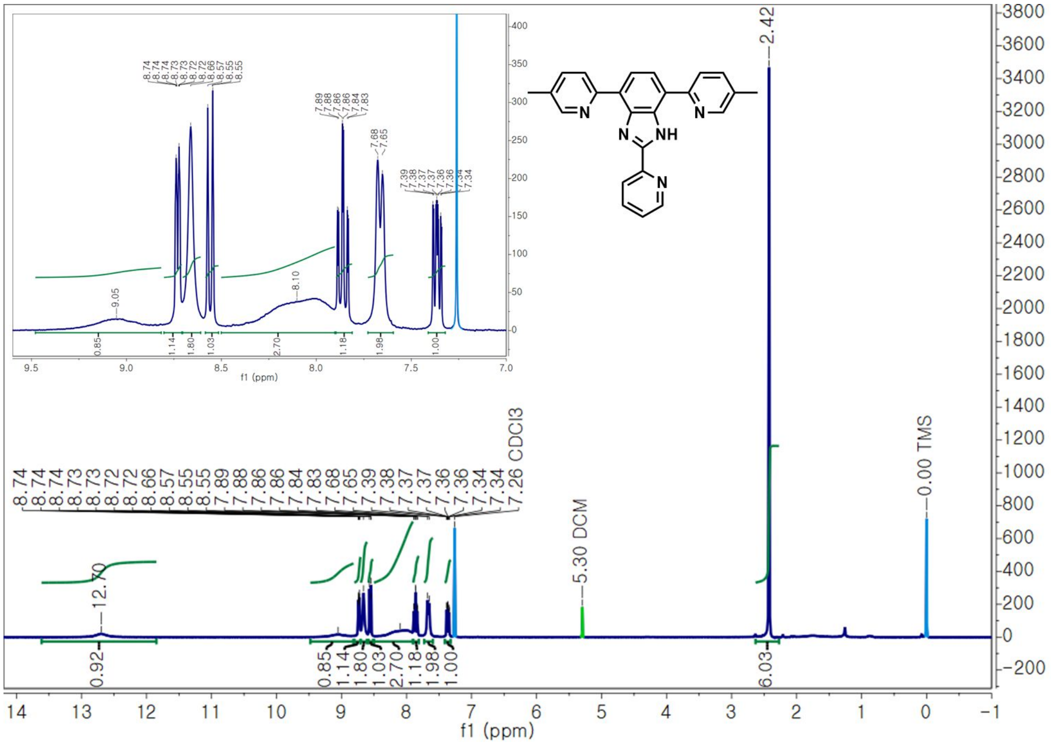

Supplementary Fig. 42. ^{1}H NMR (300 MHz) spectrum of **EliF-1d** in $CDCl_3$ (T = 298 K).

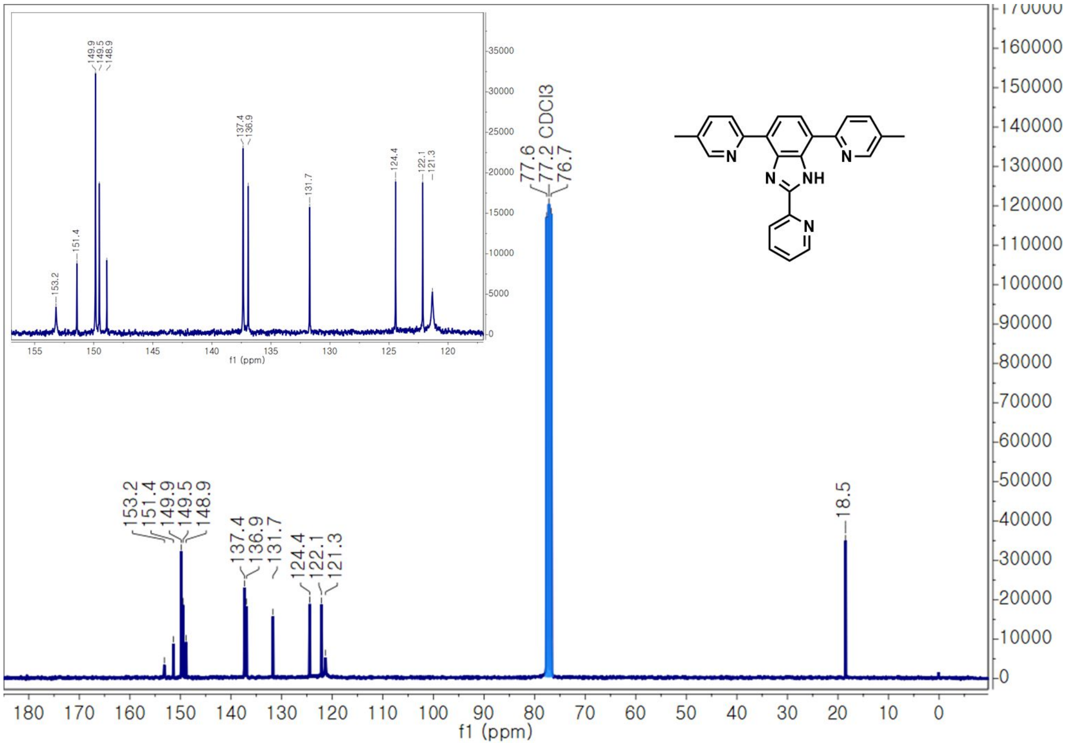

Supplementary Fig. 43. ^{13}C NMR (75 MHz) spectrum of **EliF-1d** in $CDCl_3$ (T = 298 K).

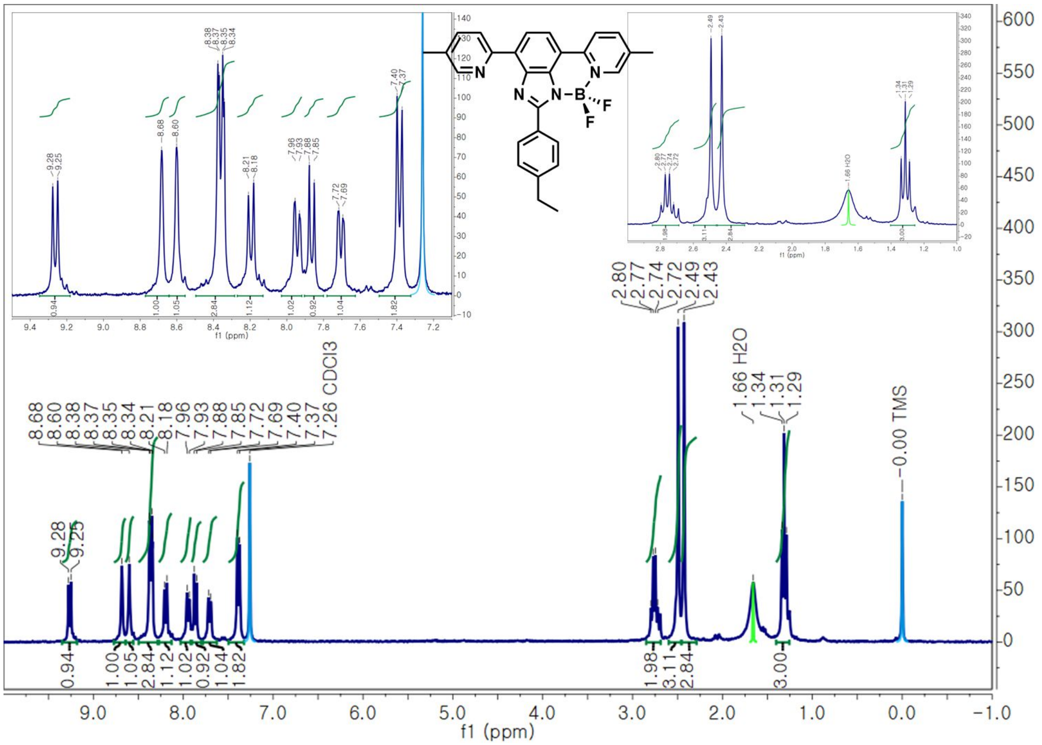

Supplementary Fig. 44. ^{1}H NMR (300 MHz) spectrum of **EliF-2a** in $CDCl_3$ (T = 298 K).

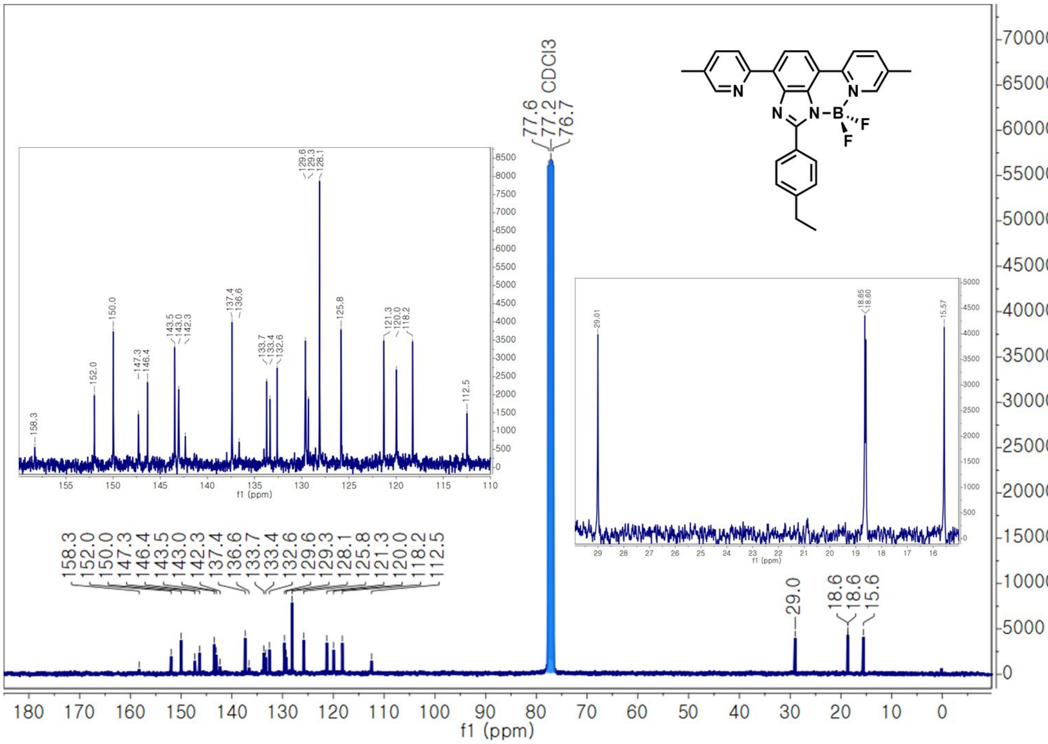

Supplementary Fig. 45. ^{13}C NMR (75 MHz) spectrum of **EliF-2a** in $CDCl_3$ (T = 298 K).

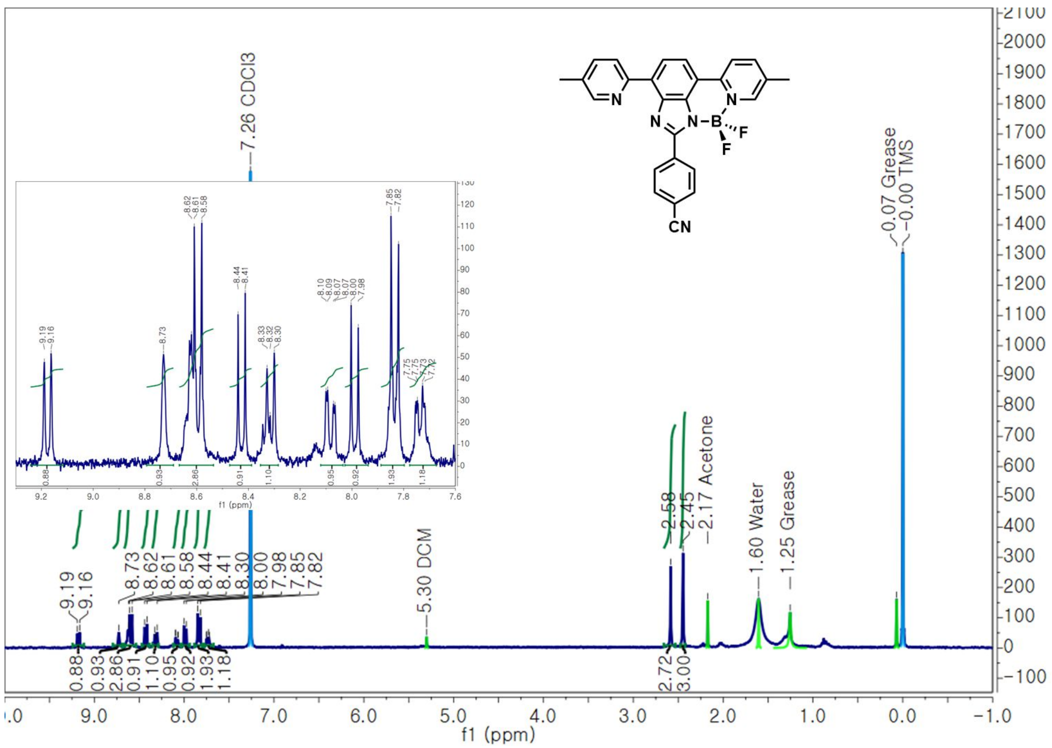

Supplementary Fig. 46. ^{1}H NMR (300 MHz) spectrum of **EliF-2b** in $CDCl_3$ (T = 298 K).

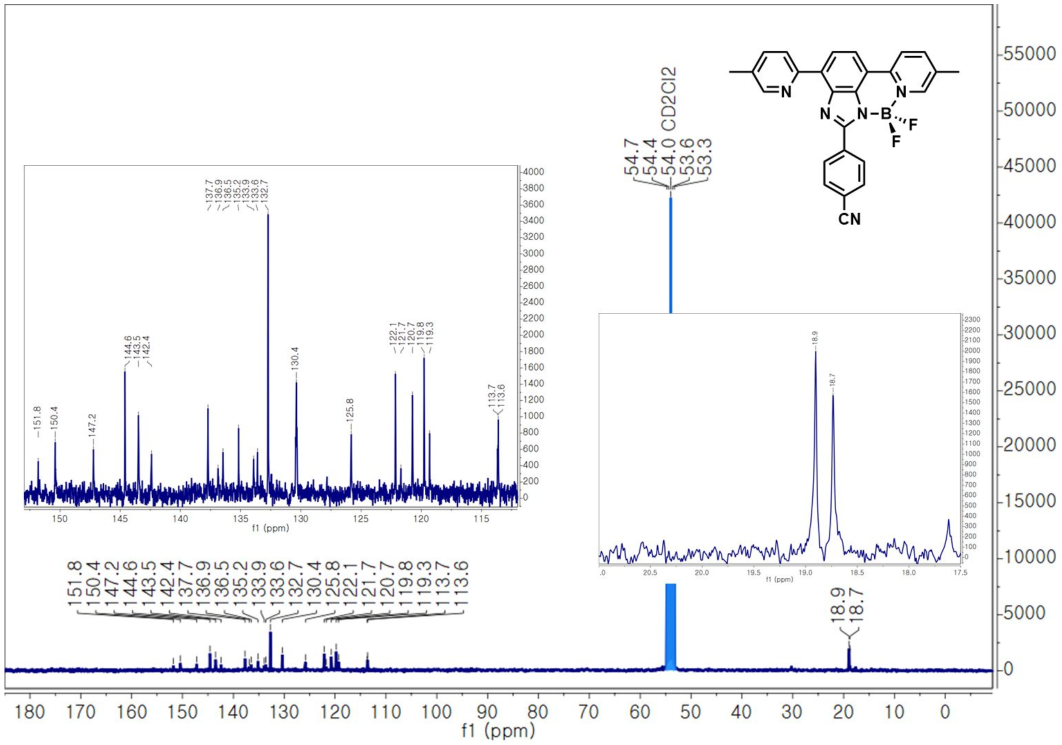

Supplementary Fig. 47. ^{13}C NMR (75 MHz) spectrum of **EliF-2b** in CD_2Cl_2 (T = 298 K).

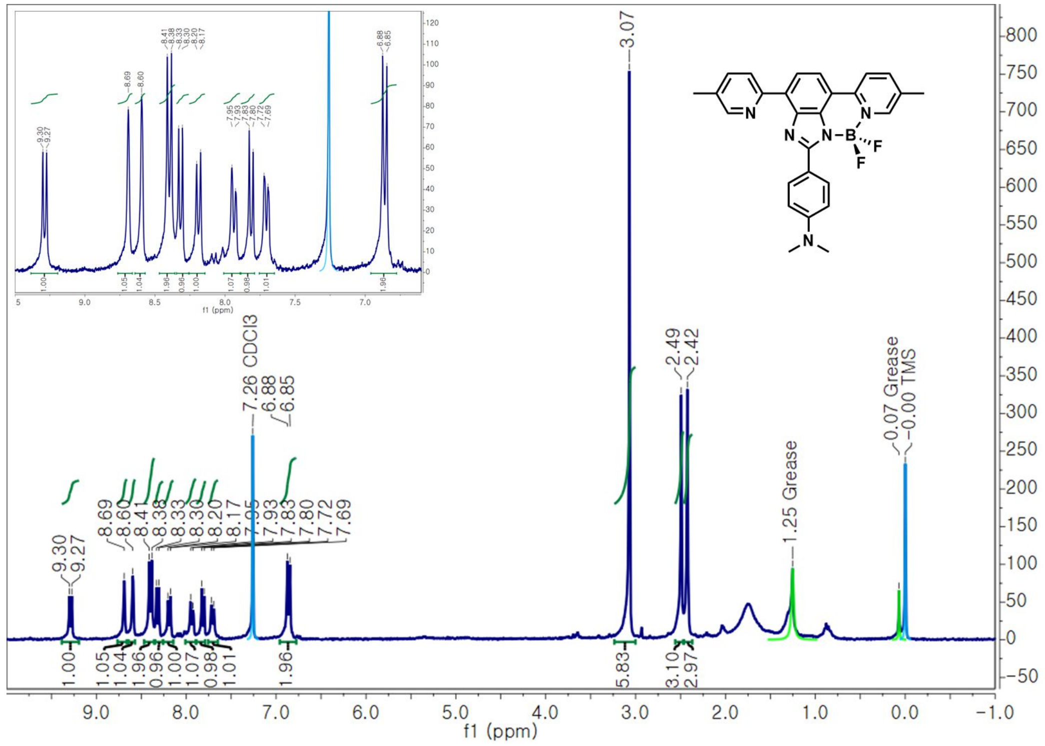

Supplementary Fig. 48. ^{1}H NMR (300 MHz) spectrum of **EliF-2c** in $CDCl_3$ (T = 298 K).

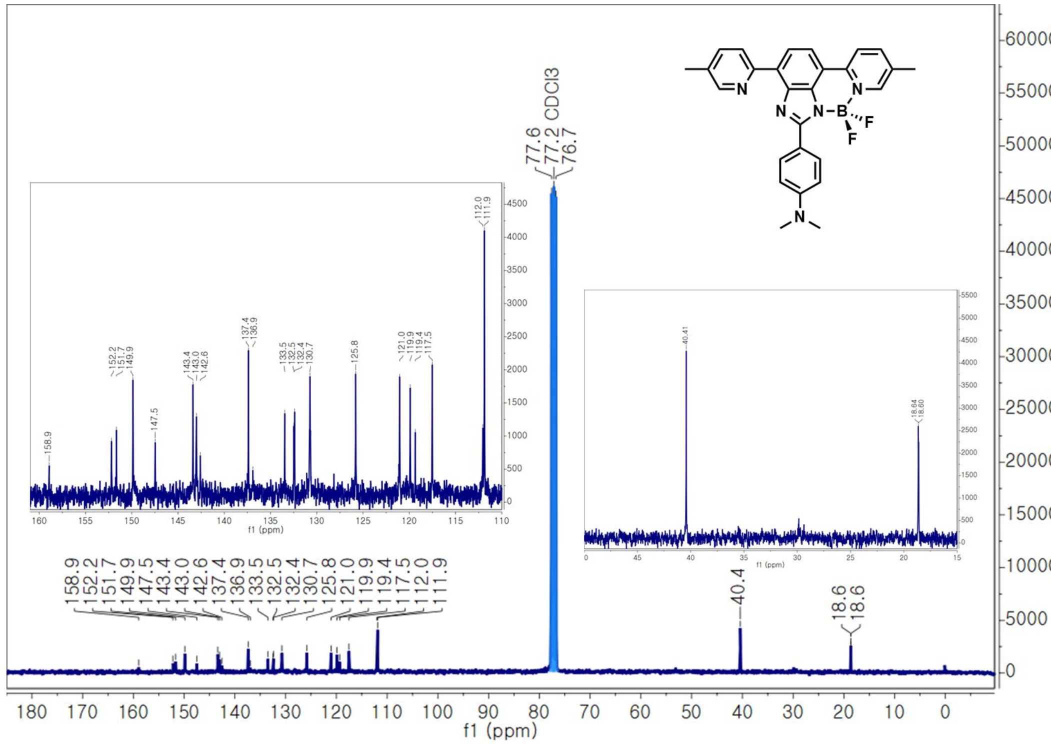

Supplementary Fig. 49. ^{13}C NMR (75 MHz) spectrum of **EliF-2c** in $CDCl_3$ (T = 298 K).

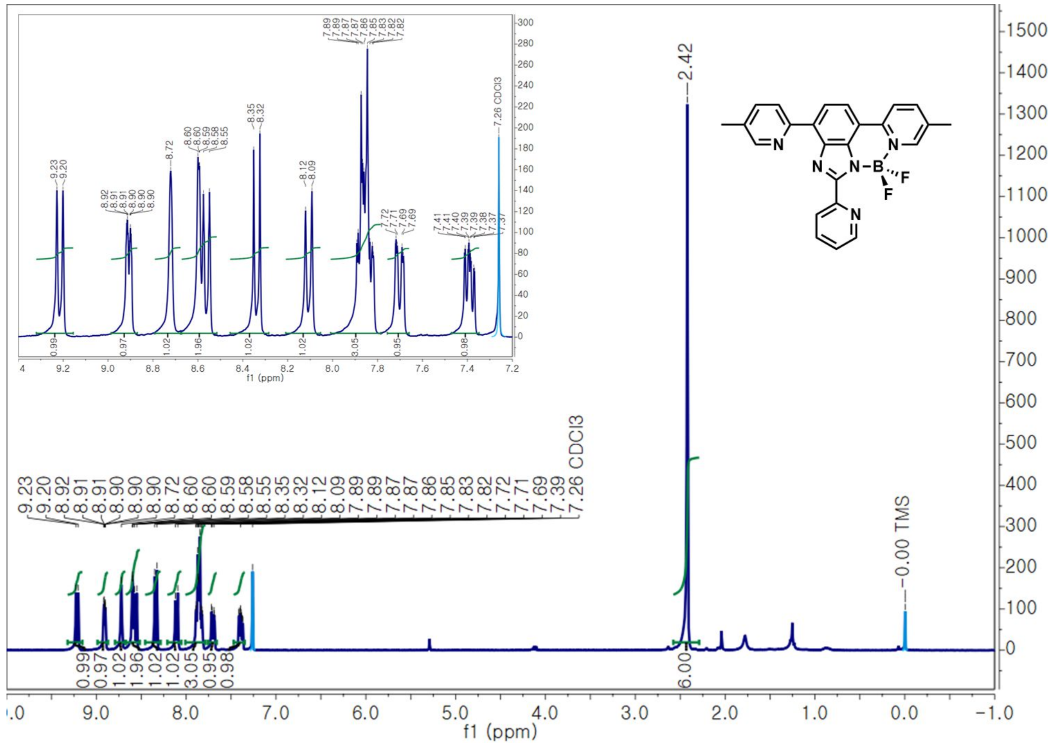

Supplementary Fig. 50. ^{1}H NMR (300 MHz) spectrum of **EliF-2d** in $CDCl_3$ (T = 298 K).

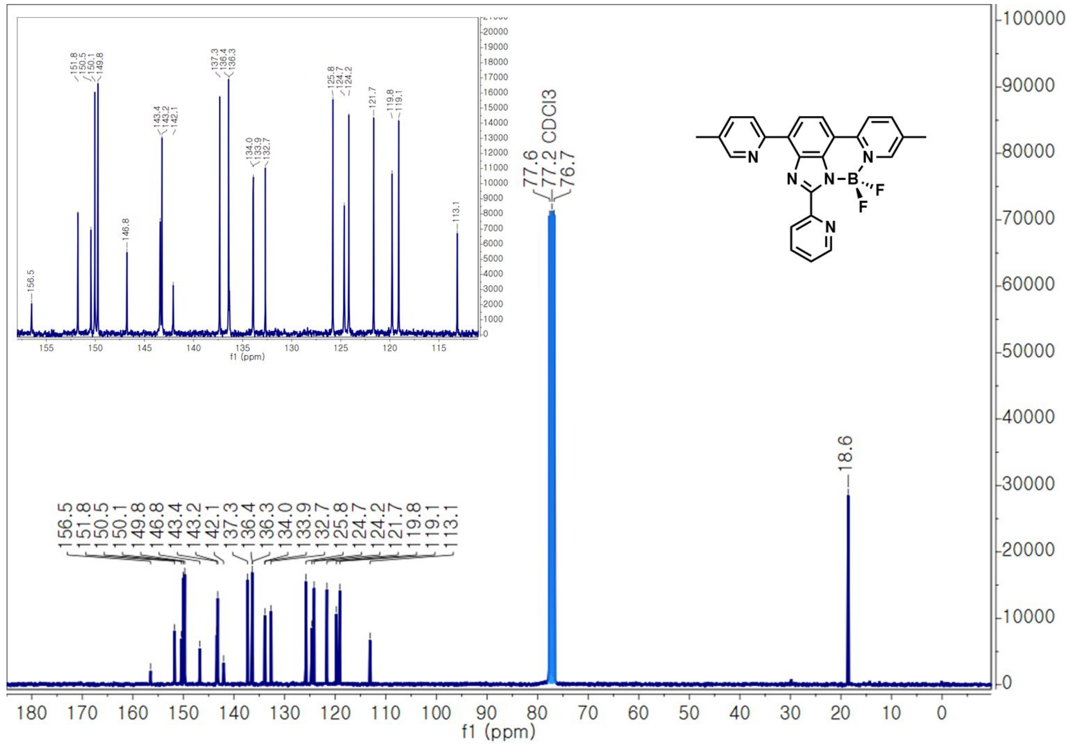

Supplementary Fig. 51. ^{13}C NMR (75 MHz) spectrum of **EliF-2d** in $CDCl_3$ (T = 298 K).

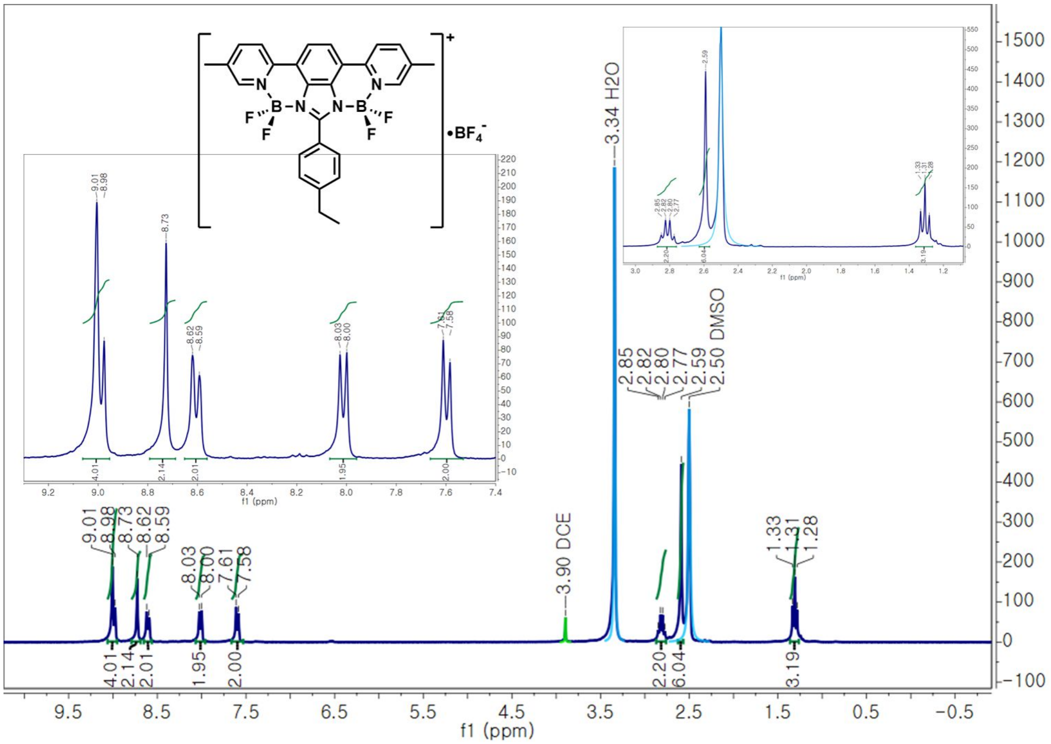

Supplementary Fig. 52. ^{1}H NMR (300 MHz) spectrum of **EliF-3a** in DMSO-d_6 (T = 298 K).

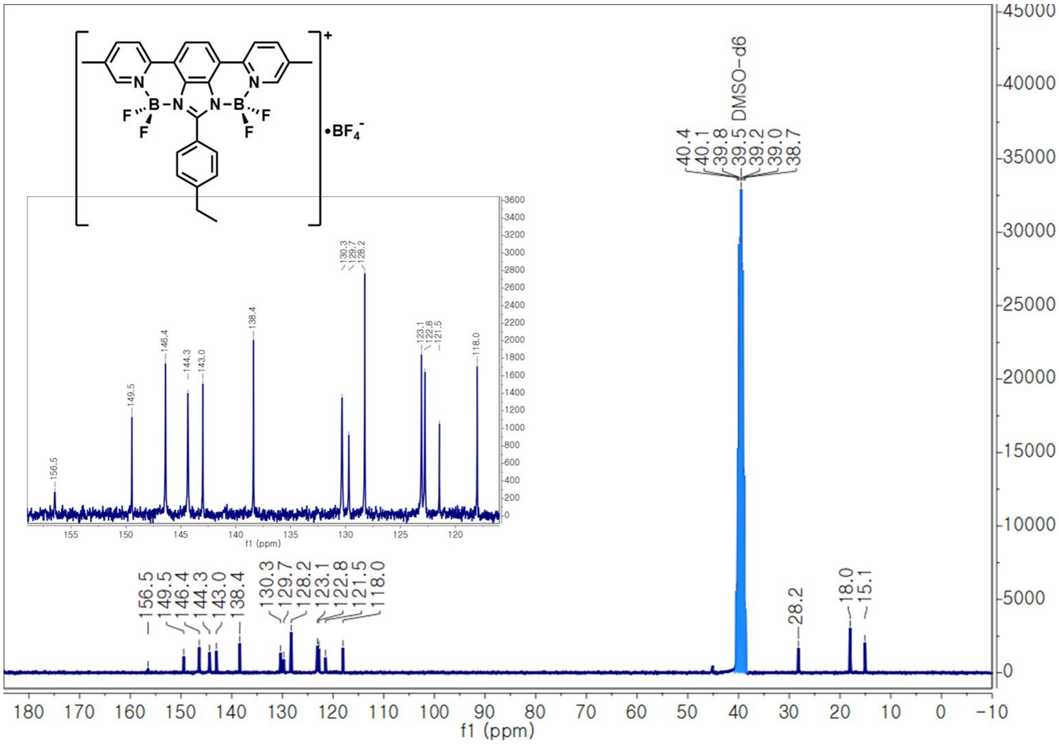

Supplementary Fig. 53. ^{13}C NMR (75 MHz) spectrum of **EliF-3a** in DMSO-d_6 (T = 298 K).

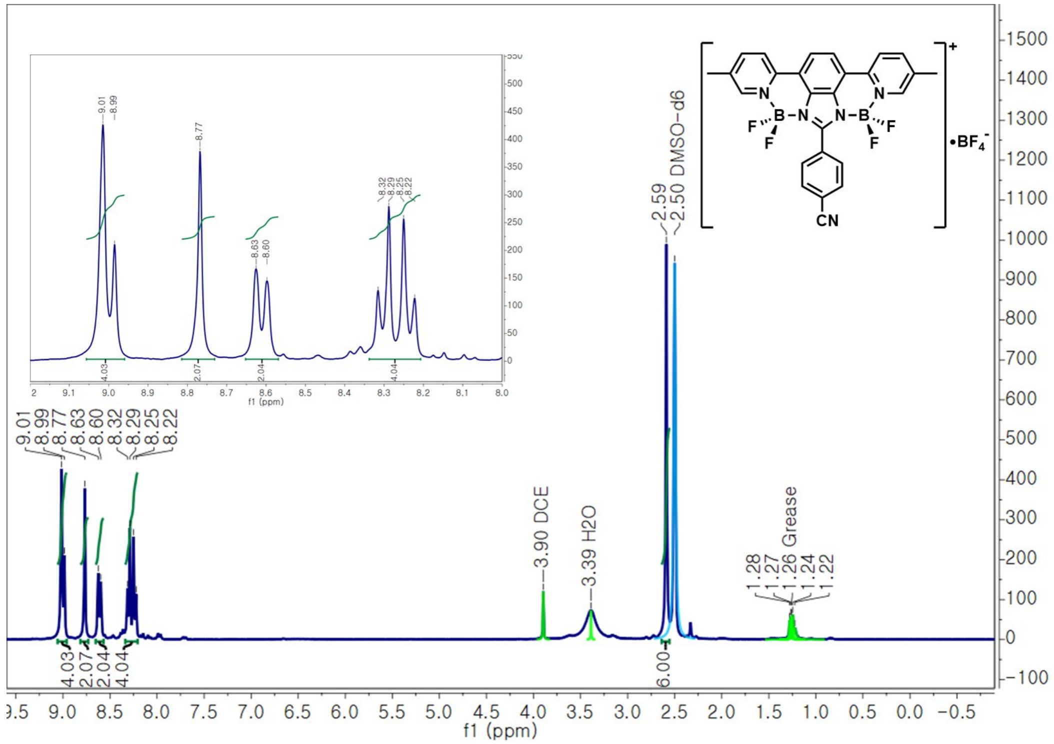

Supplementary Fig. 54. ^{1}H NMR (300 MHz) spectrum of **EliF-3b** in DMSO-d_6 (T = 298 K).

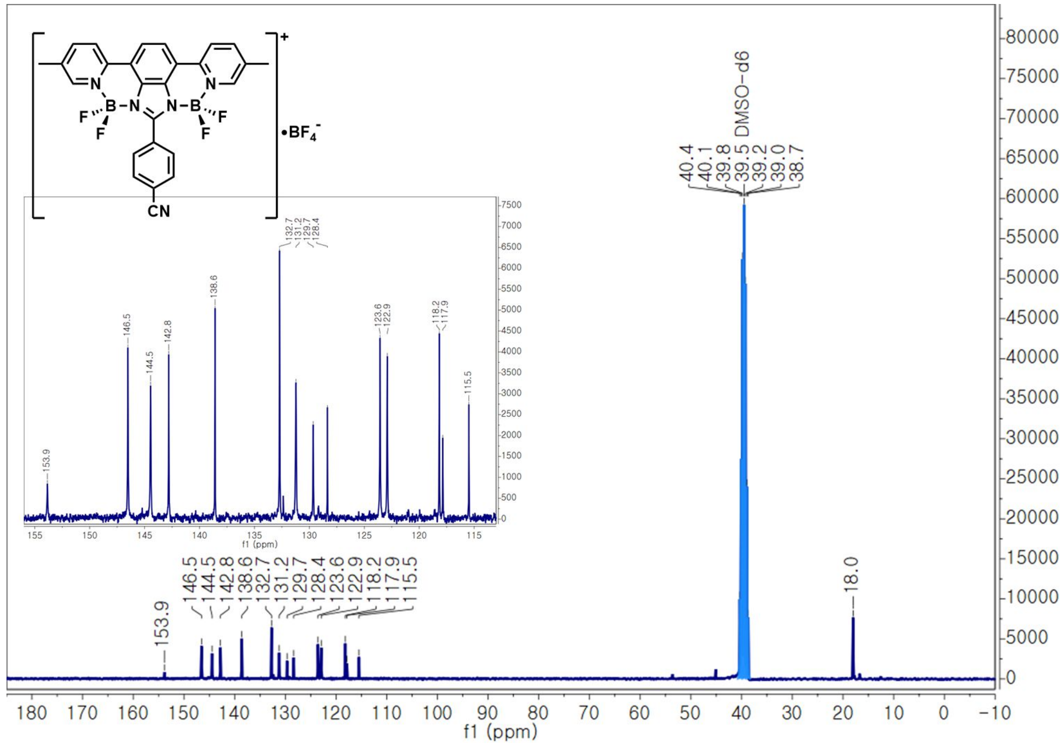

Supplementary Fig. 55. ^{13}C NMR (75 MHz) spectrum of **EliF-3b** in DMSO-d_6 (T = 298 K).

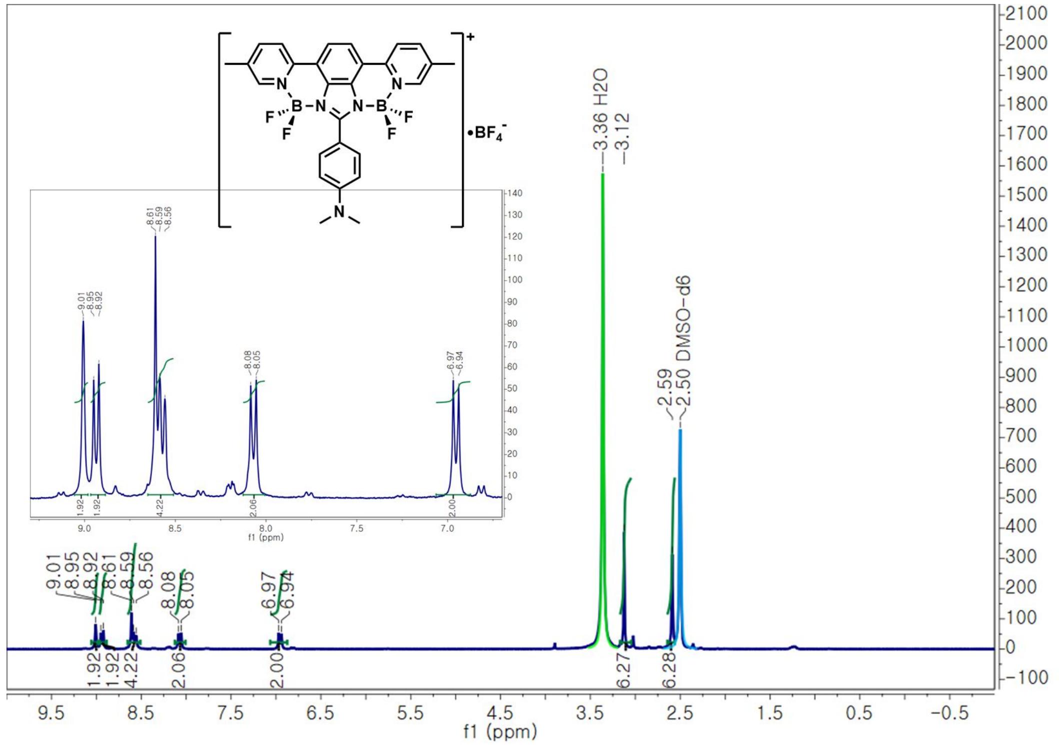

Supplementary Fig. 56. ^{1}H NMR (300 MHz) spectrum of **EliF-3c** in DMSO-d_6 (T = 298 K).

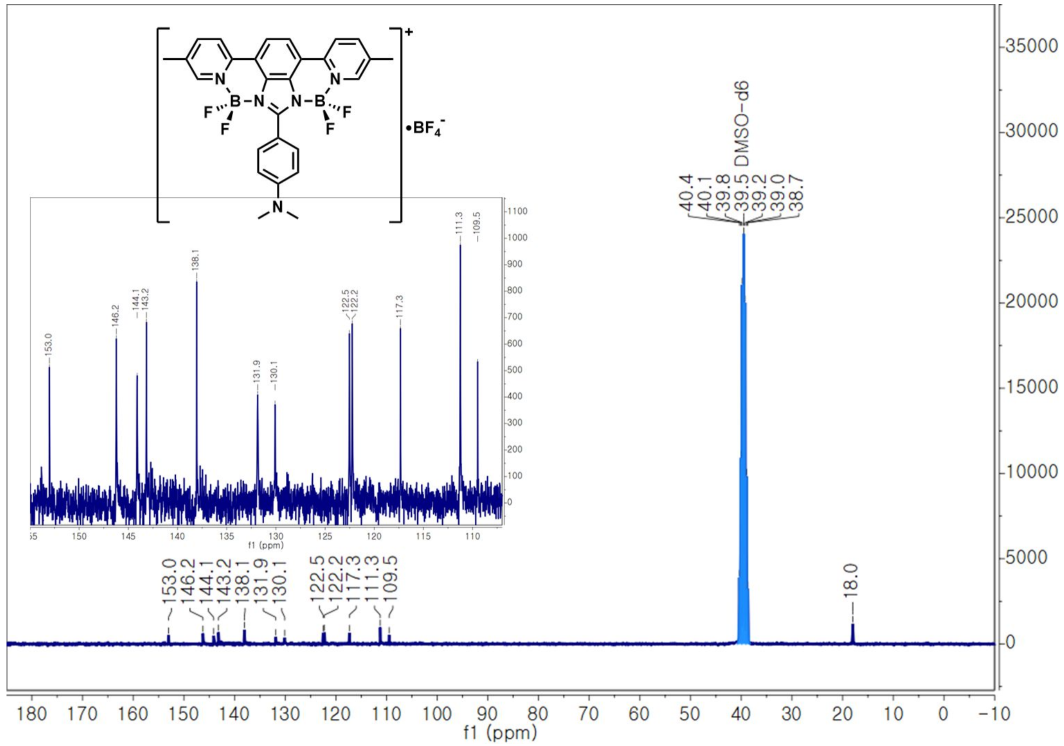

Supplementary Fig. 57. ^{13}C NMR (75 MHz) spectrum of **EliF-3c** in DMSO-d_6 (T = 298 K).

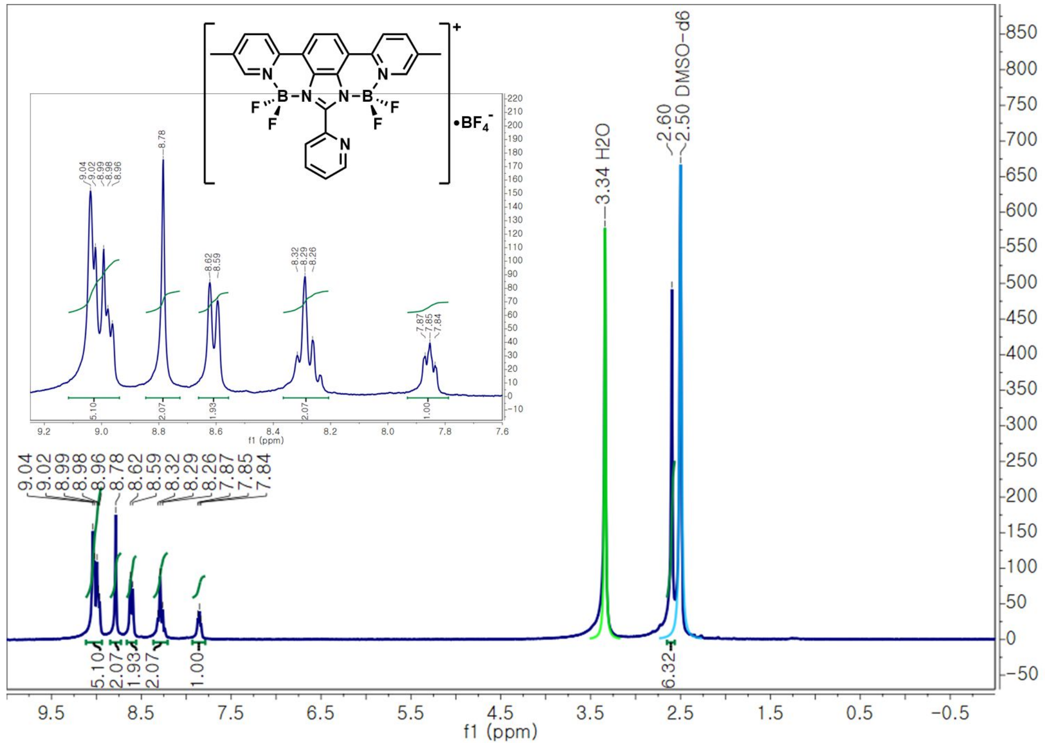

Supplementary Fig. 58. ^{1}H NMR (300 MHz) spectrum of **EliF-3d** in DMSO-d_6 (T = 298 K).

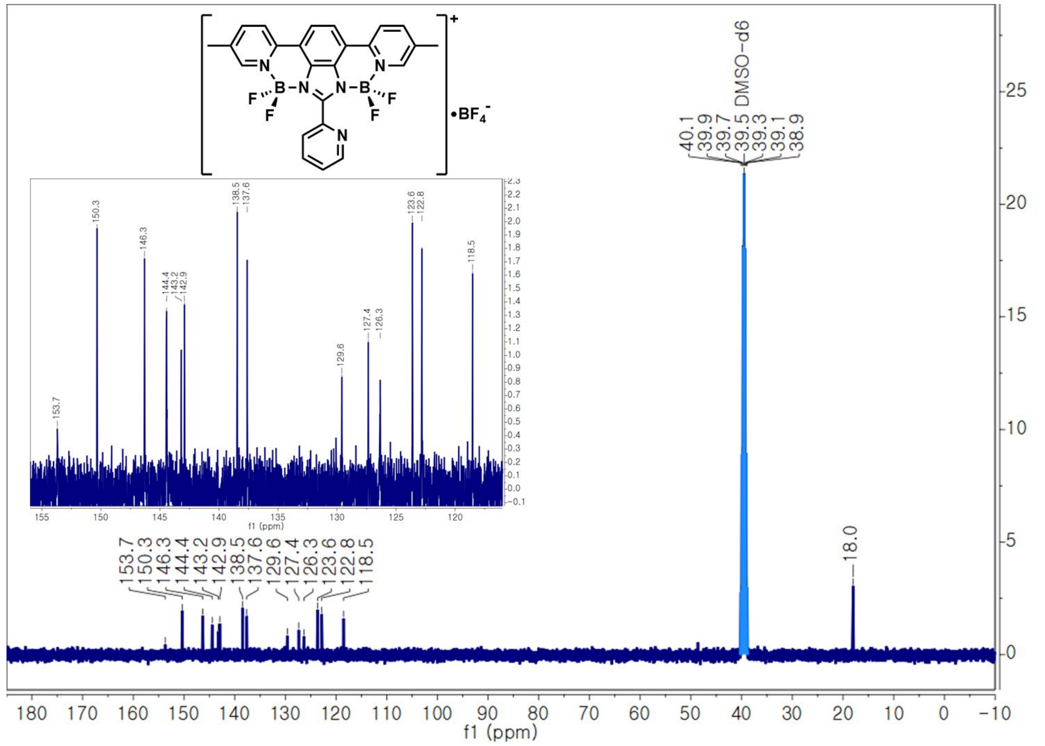

Supplementary Fig. 59. ^{13}C NMR (100 MHz) spectrum of **EliF-3d** in DMSO-d_6 (T = 298 K).

Supplementary References

1. Akhtaruzzaman, M., Tomura, M., Nishida, J.-i. & Yamashita, Y. Synthesis and Characterization of Novel Dipyridylbenzothiadiazole and Bisbenzothiadiazole Derivatives. *J. Org. Chem.* **69**, 2953–2958 (2004).

2. Schubert, U. S., Eschbaumer, C. & Heller, M. Stille-Type Cross-Coupling – An Efficient Way to Various Symmetrically and Unsymmetrically Substituted Methyl-Bipyridines: Toward New ATRP Catalysts. *Org. Lett.* **2**, 3373–3376 (2000).

3. Gu, C., Du, Z., Shen, W., Bao, X., Wen, S., Zhu, D., Wang, T., Wang, N. & Yang, R. Optical, Electrochemical, and Photovoltaic Properties of Conjugated Polymers with Dithiafulvalene as Side Chains. *J. Appl. Polym. Sci.* **132**, 41508 (2015).

4. Sheldrick, G. M. *SHELXT* - Integrated space-group and crystal-structure determination. *Acta Cryst.* **A71**, 3–8 (2015).

5. Sheldrick, G. M. Crystal structure refinement with *SHELXL*. *Acta Cryst.* **C71**, 3–8 (2015).

6. Dolomanov, O. V., Bourhis, L. J., Gildea, R. J., Howard, J. A. K. & Puschmann, H. *OLEX2*: a complete structure solution, refinement and analysis program. *J. Appl. Cryst.* **42**, 339–341 (2009).

7. Li, J., Ni, M., Lee, B., Barron, E., Hinton, D. R. & Lee, A. S. The unfolded protein response regulator GRP78/BiP is required for endoplasmic reticulum integrity and stress-induced autophagy in mammalian cells. *Cell Death Differ.* **15**, 1460–1471 (2008).

8. Merksamer, P. I., Trusina, A. & Papa, F. R. Real-time redox measurements during endoplasmic reticulum stress reveal interlinked protein folding functions. *Cell* **135**, 933–947 (2008).

9. Kohler, B., Anguissola, S., Concannon, C. G., Rehm, M., Kogel, D. & Prehn, J. H. Bid participates in genotoxic drug-induced apoptosis of HeLa cells and is essential for death receptor ligands' apoptotic and synergistic effects. *PLOS ONE* **3**, e2844 (2008).

10. Haze, K., Yoshida, H., Yanagi, H., Yura, T. & Mori, K. Mammalian transcription factor ATF6 is synthesized as a transmembrane protein and activated by proteolysis in response to endoplasmic reticulum stress. *Mol. Biol. Cell* **10**, 3787–3799 (1999).

11. Hansch, C., Leo, A. & Taft, R. W. A Survey of Hammett Substituent Constants and Resonance and Field Parameters. *Chem. Rev.* **91**, 165–195 (1991).

12. Hoge, B. & Bader, J. A Qualitative Scale for the Electron Withdrawing Effect of Substituted Phenyl Groups and Heterocycles. *J. Fluor. Chem.* **128**, 857–861 (2007).

13. The C–F bond distances were obtained from DFT geometry optimized structures of the Ar–F model computed at B3LYP/6-311+G** level.
