# Supplementary material for: Orthogonally-tunable and ER-targeting fluorophores detect avian influenza virus early infection
Source: Nat Commun. 2022 Oct 4;13:5841. doi: 10.1038/s41467-022-33586-1 (PMC9529605; doi:10.1038/s41467-022-33586-1)
Supplement: Supplementary file 3 — Description of Additional Supplementary Files [file 41467_2022_33586_MOESM3_ESM.pdf]

Title: Supplementary Data 1

Description: Results of thermal proteome profiling experiments, list of the primary hit proteins and their Tm shifts, and results of gene ontology enrichment analysis.
