# Supplementary material for: Orthogonally-tunable and ER-targeting fluorophores detect avian influenza virus early infection
Source: Nat Commun. 2022 Oct 4;13:5841. doi: 10.1038/s41467-022-33586-1 (PMC9529605; doi:10.1038/s41467-022-33586-1)
Supplement: Supplementary file 5 — Reporting Summary [file 41467_2022_33586_MOESM5_ESM.pdf]

nature portfolio

Corresponding author(s): Dongwhan Lee; Jun-Seok Lee

Last updated by author(s): Sep 14, 2022

Reporting Summary

Nature Portfolio wishes to improve the reproducibility of the work that we publish. This form provides structure for consistency and transparency in reporting. For further information on Nature Portfolio policies, see our Editorial Policies and the Editorial Policy Checklist.

Statistics

For all statistical analyses, confirm that the following items are present in the figure legend, table legend, main text, or Methods section.

n/a	Confirmed	
☐	☒	The exact sample size (*n*) for each experimental group/condition, given as a discrete number and unit of measurement
☐	☒	A statement on whether measurements were taken from distinct samples or whether the same sample was measured repeatedly
☐	☒	The statistical test(s) used AND whether they are one- or two-sided<br>*Only common tests should be described solely by name; describe more complex techniques in the Methods section.*
☒	☐	A description of all covariates tested
☒	☐	A description of any assumptions or corrections, such as tests of normality and adjustment for multiple comparisons
☐	☒	A full description of the statistical parameters including central tendency (e.g. means) or other basic estimates (e.g. regression coefficient) AND variation (e.g. standard deviation) or associated estimates of uncertainty (e.g. confidence intervals)
☐	☒	For null hypothesis testing, the test statistic (e.g. *F*, *t*, *r*) with confidence intervals, effect sizes, degrees of freedom and *P* value noted<br>*Give P values as exact values whenever suitable.*
☒	☐	For Bayesian analysis, information on the choice of priors and Markov chain Monte Carlo settings
☒	☐	For hierarchical and complex designs, identification of the appropriate level for tests and full reporting of outcomes
☒	☐	Estimates of effect sizes (e.g. Cohen's *d*, Pearson's *r*), indicating how they were calculated

Our web collection on statistics for biologists contains articles on many of the points above.

Software and code

Policy information about availability of computer code

Data collection	Standard commercial softwares were used for data collection.<br>Leica DMi8 microscope: Leica Application Suite X (LAS X Version: 3.7.0.20979); Nikon Eclipse Ti microscope: NIS-Elements BR (version 4.50); Gallios Flow Cytometer: Kaluza software (version 2.1); Molecular Devices SpectraMax: SoftMax Pro (version 5.4); Operetta: Harmony 3.1 software; LC-MS/MS: Xcalibur (version 4.1)<br>Density functional theory (DFT) calculation data were obtained by using Gaussian 16, Revision B.01 software.
Data analysis	Data analysis, including statistical analysis, was performed using OriginPro 2019 (version 9.6.0.172). FlowJo V10 and Kaluza software (version 2.1) were used to analyze flow cytometry data collected using the Gallios Flow Cytometer. Harmony V3.1 software was used to analyze immunocytochemistry (ICC) data collected using the Operetta High Contents Image System. LC-MS/MS data was analyzed with Proteome Discoverer V2.4 and R (version 4.1.1). The colocalization analysis was performed by using Fiji/ImageJ (version 1.46r)

For manuscripts utilizing custom algorithms or software that are central to the research but not yet described in published literature, software must be made available to editors and reviewers. We strongly encourage code deposition in a community repository (e.g. GitHub). See the Nature Portfolio guidelines for submitting code & software for further information.

Data

Policy information about availability of data

All manuscripts must include a data availability statement. This statement should provide the following information, where applicable:

- Accession codes, unique identifiers, or web links for publicly available datasets
- A description of any restrictions on data availability
- For clinical datasets or third party data, please ensure that the statement adheres to our policy

The X-ray crystallographic coordinates for structures reported in this study have been deposited at the Cambridge Crystallographic Data Centre (CCDC), under deposition numbers 2003264 [https://www.ccdc.cam.ac.uk/structures/Search?ccdc=2003264] (EliF-1a), 2003269 [https://www.ccdc.cam.ac.uk/structures/Search?ccdc=2003269] (EliF-1c), 2003265 [https://www.ccdc.cam.ac.uk/structures/Search?ccdc=2003265] (EliF-1d), 2003263 [https://www.ccdc.cam.ac.uk/structures/Search?ccdc=2003263] (EliF-2a), 2003267 [https://www.ccdc.cam.ac.uk/structures/Search?ccdc=2003267] (EliF-2b), 2003268 [https://www.ccdc.cam.ac.uk/structures/Search?ccdc=2003268] (EliF-2d), 2003266 [https://www.ccdc.cam.ac.uk/structures/Search?ccdc=2003266] (EliF-3d). These data can be obtained free of charge from The Cambridge Crystallographic Data Centre via www.ccdc.cam.ac.uk/data_request/cif. The MS/MS/MS data obtained in this study were searched using Uniprot (Taxonomy: Homo sapiens) [https://www.uniprot.org/uniprotkb?facets=reviewed:true&query=(taxonomy_id: 9606)]. The proteomics dataset generated in this study is provided in the Supplementary Data 1 and available in the MassIVE repository under accession code MSV000089586 [https://massive.ucsd.edu/ProteoSAFe/dataset.jsp?accession=MSV000089586]. All data that support findings of this study are available from the corresponding authors upon request. Supplementary methods, NMR spectra, supplementary table, and supplementary figures are available in the Supplementary Information. Supplementary Data 1 includes the results of thermal proteome profiling experiments, list of the primary hit proteins and their Tm shifts, and results of gene ontology enrichment analysis. Source data for Fig. 7e, Supplementary Figs. 12, 14, 15, 16, and 31a are provided as a Source Data file.

Human research participants

Policy information about studies involving human research participants and Sex and Gender in Research.

Reporting on sex and gender	NA
Population characteristics	NA
Recruitment	NA
Ethics oversight	NA

Note that full information on the approval of the study protocol must also be provided in the manuscript.

Field-specific reporting

Please select the one below that is the best fit for your research. If you are not sure, read the appropriate sections before making your selection.

☒ Life sciences ☐ Behavioural & social sciences ☐ Ecological, evolutionary & environmental sciences

For a reference copy of the document with all sections, see nature.com/documents/nr-reporting-summary-flat.pdf

Life sciences study design

All studies must disclose on these points even when the disclosure is negative.

Sample size	Sample sizes were not predetermined based on statistical methods, but were chosen according to the standards of the field. Specifically, cellular toxicity and photo-toxicity measurement for the EliF compounds, fluorescence imaging for EliF compounds were measured at least three independent biological replicates. For FACS analysis, two independent biological replicates were tested, which generated a sufficient number of single cell trajectories and gave sufficient statistics for the effect of interest.
Data exclusions	No data were excluded.
Replication	For key findings, at least 2 independent experiments were performed in order to check the reproducibility of results. These experiments include fluorescence colocalization, target protein identification, flow cytometry, qRT-PCR. All attempts of replication were successful. Reported results were consistently replicated across multiple experiments with all replicates generating similar results.
Randomization	HeLa, SK-OV3, SH-SY5Y cells were chosen based on previously published and unpublished data for their different infection profile. Human tracheal epithelial cells (HTEpC), RPMI 2650 as nasal septum originated respiratory-related cancer cell and HUVEC as normal human cells were chosen to provide infection trends in respiratory tract originated cells.<br>No randomization was necessary for this study because investigators were comparing fluorescence response under well controlled conditions (e.g. identical gain condition of FACS). No human or animal subjects were used in the study. Randomization is not generally used in this field.
Blinding	Investigators were not blinded for fluorescence live cell imaging because it is the first feasibility examination of EliF for bioimaging. Blinding during collection was not needed because conditions were well controlled. Blinding during analysis was not feasible as the differences

between samples under different conditions were visually apparent. Blinding is also not necessary because the results are quantitative and did not require subjective judgment or interpretation. For the initial examination of new bioimaging compound, blinding is not typically used in the field. For FACS analysis, researchers were blinded for the quantification of FACS signals upon influenza virus infection.

Reporting for specific materials, systems and methods

We require information from authors about some types of materials, experimental systems and methods used in many studies. Here, indicate whether each material, system or method listed is relevant to your study. If you are not sure if a list item applies to your research, read the appropriate section before selecting a response.

Materials & experimental systems

n/a	Involved in the study
☐	☒ Antibodies
☐	☒ Eukaryotic cell lines
☒	☐ Palaeontology and archaeology
☒	☐ Animals and other organisms
☒	☐ Clinical data
☒	☐ Dual use research of concern

Methods

n/a	Involved in the study
☒	☐ ChIP-seq
☐	☒ Flow cytometry
☒	☐ MRI-based neuroimaging

Antibodies

Antibodies used

For the staining of avian influenza infected cells, a 1:100 dilution of anti-matrix protein 1 (M1) antibody (mouse; abcam, cat. no. ab22396; Clone number: GA2B) was used. Unbound antibodies were washed out with PBS-T (PBS containing 0.1% Tween-20) for three times and incubated 1:3000 dilution of anti-mouse Alexa488 (rabbit; Thermo Fisher Scientific, cat. no. A-11011; RRID: AB_143157) for 1 h.

Validation

Anti-matrix protein 1 (M1) antibody is validated by the manufacturer (abcam), noting "our Abpromise guarantee covers the use of ab22396 in the following tested applications (Flow Cyt, IHC-P, WB, ICC/IF)" in their website.

Eukaryotic cell lines

Policy information about cell lines and Sex and Gender in Research

Cell line source(s)

HeLa (Korean Cell Line Bank, KCLB No. 10002), SK-OV3 (Korean Cell Line Bank, KCLB No. 30077), SH-SY5Y (Korean Cell Line Bank, KCLB No. 22266) and RPMI 2650 (Korean Cell Line Bank, KCLB No. 10030) were procured from the Korean Cell Line Bank(Seoul, South Korea). Human Tracheal Epithelial Cells (HTEpC) (PromoCell, cat. no. C-12644) and Human Umbilical Vein Endothelial Cells (HUVEC) (Cell Engineering For Origin, cat. no. CEFOgro-HUVEC) were procured from PromoCell (Heidelberg, Germany), and Cell Engineering For Origin (Seoul, South Korea), respectively.

Authentication

Korean Cell Line Bank provide Short tandem repeat (STR) result for human cell line authentication. Promocell noted: Cells are ethically collected along with donor documentation and validated for uniqueness using robust phenotype markers.

Mycoplasma contamination

All cell lines tested negative for mycoplasma contamination.

Commonly misidentified lines
(See ICLAC register)

No commonly misidentified cells were used in this study.

Flow Cytometry

Plots

Confirm that:

☒ The axis labels state the marker and fluorochrome used (e.g. CD4-FITC).

☒ The axis scales are clearly visible. Include numbers along axes only for bottom left plot of group (a 'group' is an analysis of identical markers).

☒ All plots are contour plots with outliers or pseudocolor plots.

☒ A numerical value for number of cells or percentage (with statistics) is provided.

Methodology

Sample preparation

The cells were infected with the H1N1 virus for 12 and 24 h, and treated subsequently with EliF-2c (10 µM) for 30 min. The cells were trypsinized, harvested, and centrifuged for 5 min at 1,200 rpm (= 136 g). The supernatant was discarded, and the remaining cell pellets were resuspended in PBS.

Instrument	Gallios Flow Cytometer (Beckman Coulter, USA)
Software	Data collection was performed with Kaluza software (Beckman Coulter, USA). The data was processed with FlowJo Single Cell Analysis Software v10(FlowJo, LLC, Ashland, OR, USA).
Cell population abundance	The resulting samples were subjected to flow cytometry analysis by counting 10,000 events for each sample.
Gating strategy	Firstly, singlet cells were gated in FS-A and FL10-A ($\lambda exc = 405$ nm and $\lambda em = 530–570$ nm) plots to exclude debris and non-singlet events. Secondly, gates were constructed based on laser scatter properties (FSC-A and SSC-A)

☐ Tick this box to confirm that a figure exemplifying the gating strategy is provided in the Supplementary Information.
